# Supplementary material for: Understanding catalytic synergy in dinuclear polymerization catalysts for sustainable polymers
Source: Nat Commun. 2023 Aug 8;14:4783. doi: 10.1038/s41467-023-40284-z (PMC10409799; doi:10.1038/s41467-023-40284-z)
Supplement: Supplementary file 1 — Supplementary Information [file 41467_2023_40284_MOESM1_ESM.pdf]

Supplementary Information
Understanding Catalytic Synergy in Dinuclear Polymerization Catalysts for Sustainable Polymers

Francesca Fiorentini[1], Wilfred T. Diment[1], Arron C. Deacy[1], Ryan W. F. Kerr[1], Stephen Faulkner[1], Charlotte K. Williams[1] *

[1] Department of Chemistry, University of Oxford, OX1 3TA, United Kingdom

*charlotte.williams@chem.ox.ac.uk

Supplementary Fig 1. Dinuclear metallate mechanism for PO/CO_2 ROCOP catalysed by Co(III)K(I) catalyst.... 8
Supplementary Fig 2. ^{1}H NMR spectrum of Co(III)Ca(II)... 8
Supplementary Fig 3. ^{13}C{^{1}H} NMR spectrum of Co(III)Ca(II)... 9
Supplementary Fig 4. COSY NMR spectrum of Co(III)Ca(II). 9
Supplementary Fig 5. HSQC NMR spectrum of Co(III)Ca(II). 10
Supplementary Fig 6. HMBC NMR spectrum of Co(III)Ca(II). 10
Supplementary Fig 7. ^{1}H NMR spectrum of Co(III)Sr(II)... 11
Supplementary Fig 8. ^{13}C{^{1}H} NMR spectrum of Co(III)Sr(II)... 11
Supplementary Fig 9. COSY NMR spectrum of Co(III)Sr(II). 12
Supplementary Fig 10. HSQC NMR spectrum of Co(III)Sr(II). 12
Supplementary Fig 11. HMBC NMR spectrum of Co(III)Sr(II). 13
Supplementary Fig 12. ^{1}H NMR spectrum of Co(III)Ba(II).. 13
Supplementary Fig 13. ^{13}C{^{1}H} NMR spectrum of Co(III)Ba(II).. 14
Supplementary Fig 14. COSY NMR spectrum of Co(III)Ba(II). 14
Supplementary Fig 15. HSQC NMR spectrum of Co(III)Ba(II). 15
Supplementary Fig 16. HMBC NMR spectrum of Co(III)Ba(II). 15
Supplementary Fig 17. IR spectrum of Co(III)Ca(II). 16
Supplementary Fig 18. IR spectrum of Co(III)Sr(II). 16
Supplementary Fig 19. IR spectrum of Co(III)Ba(II). 17
Supplementary Fig 20. Variable temperature (VT) spectrum of Co(III)Ca(II) showing the development of a new acetate peak (j) at 230 K and the splitting of the original acetate peak into two at 190 K (i', i''). 18
Supplementary Fig 21. Molecular structure of Co(III)Ca(II), determined by X-ray diffraction, with hydrogen atoms and solvent omitted for clarity. Thermal ellipsoids are represented at 40% probability. Selected bond lengths and angles are listed in Table S10 and S11 respectively. 19
Supplementary Fig 22: Molecular structure of Co(III)Sr(II), determined by X-ray diffraction, with hydrogen atoms and solvent omitted for clarity. Thermal ellipsoids are represented at 40% probability. Selected bond lengths and angles are listed in Table S10 and S11 respectively. 19
Supplementary Fig 23. Molecular structure of Co(III)Ba(II), determined by X-ray diffraction, with hydrogen atoms and solvent omitted for clarity. Thermal ellipsoids are represented at 40% probability. Selected bond lengths and angles are listed in Table S10 and S11 respectively. 20
Supplementary Fig 24: Molecular structure of Co(III)Na(I), determined by X-ray diffraction, with hydrogen atoms and solvent omitted for clarity. Thermal ellipsoids are represented at 40% probability.[2] 20
Supplementary Fig 25 Molecular structure of Co(III)K(I), determined by X-ray diffraction, with hydrogen atoms and solvent omitted for clarity. Thermal ellipsoids are represented at 40% probability.[2] 21
Supplementary Fig 26: A. Molecular structure of Co(III)Rb(I), determined by X-ray diffraction, with selected hydrogen atoms and outer sphere solvent omitted for clarity. Thermal ellipsoids are represented at 20% probability; B. Illustrated dimeric structure of Co(III)Rb(I) crystal structure for clarity.[2] 21
Supplementary Fig 27. Molecular structure of Co(III)Rb(I) half-dimer, determined by X-ray diffraction, with selected hydrogen atoms and outer sphere solvent omitted for clarity. Thermal ellipsoids are represented at 40% probability.[2] 22
Supplementary Fig 28: Plot of $\ln([PO]_t/[PO]_0)$ against time for PO/CO_2 ROCOP catalysed by Co(III)Ca(II) to calculate k_{obs}. (Every 52nd point shown). 22
Supplementary Fig 29: Plot of $\ln([PO]_t/[PO]_0)$ against time for PO/CO_2 ROCOP catalysed by Co(III)Sr(II) to calculate k_{obs}. (Every 75th point shown)... 23
Supplementary Fig 30: Plot of $\ln([PO]_t/[PO]_0)$ against time for PO/CO_2 ROCOP catalysed by Co(III)Ba(II) to calculate k_{obs}. (Every 75th point shown)... 23
Supplementary Fig 31. GPC trace for PO/CO_2 polymerisation with Co(III)Ca(II) in THF, using narrow dispersity polystyrene standards. 25
Supplementary Fig 32. GPC trace for PO/CO_2 polymerisation with Co(III)Sr(II) in THF, using narrow dispersity polystyrene standards. 25
Supplementary Fig 33. GPC trace for PO/CO_2 polymerisation with Co(III)Ba(II) in THF, using narrow dispersity polystyrene standards. 26

Supplementary Fig 34. Plot of k_{obs} and TOF against log(K) (where K is the equilibrium constant for M(I/II) coordination within 18-crown-6 for a 1:1 reaction in aqueous solution at 25 °C) for PO/CO_2 ROCOP catalysed by Co(III)M(I/II).[3] 26
Supplementary Fig 35. Plot of selectivity for PPC against log(K) (where K is the equilibrium constant for M(I/II) coordination within 18-crown-6 for a 1:1 reaction in aqueous solution at 25 °C) for PO/CO_2 ROCOP catalysed by Co(III)M(I/II).[3] 27
Supplementary Fig 36. Representative conversion vs. time plots for PO/PA ROCOP catalysed by Co(III)M(I/II) (M(I/II) = Na(I), K(I), Ca(II), Sr(II), Ba(II)). 29
Supplementary Fig 37. GPC trace for PO/PA ROCOP catalysed by Co(III)K(I) in THF, using narrow dispersity polystyrene standards. 29
Supplementary Fig 38. GPC trace for PO/PA ROCOP catalysed by Co(III)Na(I) in THF, using narrow dispersity polystyrene standards. 30
Supplementary Fig 39. GPC trace for PO/PA ROCOP catalysed by Co(III)Ca(II) in THF, using narrow dispersity polystyrene standards. 30
Supplementary Fig 40. GPC trace for PO/PA ROCOP catalysed by Co(III)Sr(II) in THF, using narrow dispersity polystyrene standards. 31
Supplementary Fig 41. GPC trace for PO/PA ROCOP catalysed by Co(III)Ba(II) in THF, using narrow dispersity polystyrene standards. 31
Supplementary Fig 42. Plots of molar mass (blue squares and line) and dispersity (orange triangles) of polyester against conversion for PO/PA ROCOP catalysed by Co(III)Na(I). 32
Supplementary Fig 43. Plots of molar mass (blue squares and line) and dispersity (orange triangles) of polyester against conversion for PO/PA ROCOP catalysed by Co(III)K(I). 32
Supplementary Fig 44. Plots of molar mass (blue squares and line) and dispersity (orange triangles) of polyester against conversion for PO/PA ROCOP catalysed by Co(III)Ca(II). 33
Supplementary Fig 45. Plots of molar mass (blue squares and line) and dispersity (orange triangles) of polyester against conversion for PO/PA ROCOP catalysed by Co(III)Sr(II). 33
Supplementary Fig 46. Plots of molar mass (blue squares and line) and dispersity (orange triangles) of polyester against conversion for PO/PA ROCOP catalysed by Co(III)Ba(II). 34
Supplementary Fig 47. TOFs for anhydride (pink; LHS) and epoxide (blue; RHS) scope using Co(III)K(I) (0.1 mol% catalyst, 10 mol% anhydride in neat epoxide, 60 °C). The activity for PO/PA (blue/pink gradient; middle) is included as a reference. 35
Supplementary Fig 48. GPC trace for PO/PA ring-opening copolymerisation with Co(III)K(I) in THF, using narrow dispersity polystyrene standards. 36
Supplementary Fig 49. GPC trace for PO/CA (camphoric anhydride) ring-opening copolymerisation with Co(III)K(I) in THF, using narrow dispersity polystyrene standards. 37
Supplementary Fig 50. GPC trace for PO/NBA (norbornene anhydride) ring-opening copolymerisation with Co(III)K(I) in THF, using narrow dispersity polystyrene standards. 37
Supplementary Fig 51. GPC trace for PO/CA (tricyclic anhydride) ring-opening copolymerisation with Co(III)K(I) in THF, using narrow dispersity polystyrene standards. 38
Supplementary Fig 52. GPC trace for PO/THPA (tetrahydrophthalic anhydride) ring-opening copolymerisation with Co(III)K(I) in THF, using narrow dispersity polystyrene standards. 38
Supplementary Fig 53. GPC trace for BO/PA (butylene oxide) ring-opening copolymerisation with Co(III)K(I) in THF, using narrow dispersity polystyrene standards. 39
Supplementary Fig 54. GPC trace for CHO/PA (cyclohexene oxide) ring-opening copolymerisation with Co(III)K(I) in THF, using narrow dispersity polystyrene standards. 39
Supplementary Fig 55. GPC trace for SO/PA (styrene oxide) ring-opening copolymerisation with Co(III)K(I) in THF, using narrow dispersity polystyrene standards. 40
Supplementary Fig 56. Representative semi-logarithmic plot for *rac*-LA ROP using Co(III)Na(I), k_{obs} calculated from the gradient of the fit. 42
Supplementary Fig 57. Representative semi-logarithmic plot for *rac*-LA ROP using Co(III)Na(I), k_{obs} calculated from the gradient of the fit. 42

Supplementary Fig 58. Representative plot of [PLA] against time for *rac*-LA ROP using Co(III)Ca(II), conversion determined by ^{1}H NMR spectroscopy. k_{obs} is determined by fitting a sigmoidal function of the form [PLA] = A(1-exp(-k_{obs}t))^{s}, where s is the "sigmoidal factor". [4] 43
Supplementary Fig 59. Representative plot of [PLA] against time for *rac*-LA ROP using Co(III)Sr(II), conversion determined by ^{1}H NMR spectroscopy. k_{obs} is determined by fitting a sigmoidal function of the form [PLA] = A(1-exp(-k_{obs}t))^{s}, where s is the "sigmoidal factor". [4] 43
Supplementary Fig 60. Representative plot of [PLA] against time for *rac*-LA ROP using Co(III)Ba(II), conversion determined by ^{1}H NMR spectroscopy. k_{obs} is determined by fitting a sigmoidal function of the form [PLA] = A(1-exp(-k_{obs}t))^{s}, where s is the "sigmoidal factor". [4] 44
Supplementary Fig 61. GPC trace for *rac*-LA ROP catalysed by Co(III)Na(I) in THF, using narrow dispersity polystyrene standards. 44
Supplementary Fig 62. GPC trace for *rac*-LA ROP catalysed by Co(III)K(I) in THF, using narrow dispersity polystyrene standards. 45
Supplementary Fig 63. GPC trace for *rac*-LA ROP catalysed by Co(III)Ca(II) in THF, using narrow dispersity polystyrene standards. 45
Supplementary Fig 64. GPC trace for *rac*-LA ROP catalysed by Co(III)Sr(II) in THF, using narrow dispersity polystyrene standards. 46
Supplementary Fig 65: GPC trace for *rac*-LA ROP catalysed by Co(III)Ba(II) in THF, using narrow dispersity polystyrene standards. 46
Supplementary Fig 66. Plots of molar mass (blue squares and line) and dispersity (orange triangles) of PLA against conversion for *rac*-LA ROP catalysed by Co(III)Na(I). 47
Supplementary Fig 67. Plots of molar mass (blue squares and line) and dispersity (orange triangles) of PLA against conversion for *rac*-LA ROP catalysed by Co(III)K(I). 47
Supplementary Fig 69. Plots of molar mass (blue squares and line) and dispersity (orange triangles) of PLA against conversion for *rac*-LA ROP catalysed by Co(III)Sr(II). 48
Supplementary Fig 70. Plots of molar mass (blue squares and line) and dispersity (orange triangles) of PLA against conversion for *rac*-LA ROP catalysed by Co(III)Ba(II). 49
Supplementary Fig 71. Plot of k_{obs} against ionic radius of the s-block metal for *rac*-LA ROP catalysed by Co(III)M(I/II).[5] 49
Supplementary Fig 72. Representative ^{1}H{^{1}H} NMR spectrum of PLA produced from *rac*-LA ROP by Co(III)K(I) for the determination of P_i (= 0.72). 50
Supplementary Fig 73. Plot of ln(k_{obs}) vs. ln(pK_a) for PO/CO_2 ROCOP catalysed by Co(III)M(I/II) (M(I/II) = Na(I), K(I), Ca(II), Sr(II), Ba(II)). The linear fit shows that an exponential fit of k_{obs} vs. pK_a is appropriate. 51
Supplementary Fig 74. Plot of ln(TOF) vs. ln(pK_a) for PO/CO_2 ROCOP catalysed by Co(III)M(I/II) (M(I/II) = Na(I), K(I), Ca(II), Sr(II), Ba(II)). The linear fit shows that an exponential fit of TOF vs. pK_a is appropriate. 51
Supplementary Fig 75. Plot of TOF and k_{obs} against pK_a of the s-block metal for Co(III)M(I/II)-catalysed PO/CO_2 ROCOP, including values for Co(III)Cs(I) and Co(III)Rb(I). 52
Supplementary Fig 76. Plot of selectivity against pK_a of the s-block metal for Co(III)M(I/II)-catalysed PO/CO_2 ROCOP, including values for Co(III)Cs(I) and Co(III)Rb(I). 52
Supplementary Fig 77. Plot of ln(k_{obs}) vs. ln(pK_a) for PO/PA ROCOP catalysed by Co(III)M(I/II) (M(I/II) = Na(I), K(I), Ca(II), Sr(II), Ba(II)). The linear fit shows that an exponential fit of k_{obs} vs. pK_a is appropriate. 53
Supplementary Fig 78. Plot of ln(TOF) vs. ln(pK_a) for PO/PA ROCOP catalysed by Co(III)M(I/II) (M(I/II) = Na(I), K(I), Ca(II), Sr(II), Ba(II)). The linear fit shows that an exponential fit of TOF vs. pK_a is appropriate. 53
Supplementary Fig 79. Plot of [PA] against time for PO/PA ROCOP catalysed by Co(III)K(I) using 1:100:150 $[Cat]_0$:$[PA]_0$:$[PO]_0$ in THF. 54
Supplementary Fig 80. Plot of ln($[PA]_t$/$[PA]_0$) against time for PO/PA ROCOP catalysed by Co(III)K(I) using 1:100:150 $[Cat]_0$:$[PA]_0$:$[PO]_0$ in THF. 54
Supplementary Fig 81: Plot of P_i of PLA vs. the pK_a of the s-block metal for *rac*-LA ROP catalysed by Co(III)M(I/II) (M(I/II) = Na(I), K(I), Ca(II), Sr(II), Ba(II)). 55

Experimental Section

General Methods

All manipulations were performed using either a dual manifold nitrogen-vacuum Schlenk line or a nitrogen-filled glovebox. Solvents were collected from a solvent purification system, degassed using three freeze-pump-thaw cycles and stored over 3 Å molecular sieves in a nitrogen-filled glovebox. All reagents were obtained from commercial sources and used as received. The dialdehyde proligand was sourced from Enamine Ltd and Manchester Organics Ltd, and used as received. Propene oxide monomer was bought from Sigma Aldrich and dried overnight over calcium hydride and purified by fractional distillation, followed by degassing with nitrogen and stored under nitrogen. Phthalic anhydride was bought from Sigma Aldrich and purified by stirring in dry toluene overnight. The supernatant was removed by filtration and the toluene removed *in vacuo*. The resultant white solid was recrystallised from hot chloroform and sublimed under vacuum at 80 °C. Research-grade CO_2 (BOC, 99.99 %) was used and dried through a Drierlite column and two additional drying columns (Micro Torr, model number: MC1-804FV) in series before use.

NMR spectroscopic analysis was performed using a Bruker AV 400 MHz spectrometer at 298 K. Gel permeation chromatography (GPC) analysis was performed using a Shimadzu LC-20AD instrument, at 40 °C, with two mixed bed PSS SDV linear S columns in series, and with THF as eluent at a flow rate of 1 mL/min. Molar mass values were calibrated using narrow molar mass polystyrene standards.

General Synthesis of Co(III)M(I/II) (M(I/II) = Na(I), K(I), Rb(I), Cs(I), Ca(II), Sr(II), Ba(II))

Co(III)M(I) catalysts were synthesised following a literature procedure.[2] The Co(III)M(II) catalysts were synthesised following a modified literature procedure.[2] The dialdehyde pro-ligand (400 mg, 1.02 mmol), $Co(OAc)_2$ (181 mg, 1.02 mmol) and the respective metal acetate salt (1.02 mmol) were combined in dry acetonitrile (15 mL) to form a yellow-orange suspension and stirred at room temperature for 30 mins under a nitrogen atmosphere. To the suspension was added ethylene diamine (68 μL, 1.02 mmol), immediately giving a deep red-brown solution. The solution was stirred overnight at room temperature under a nitrogen atmosphere before adding acetic acid (117 μL, 2.04 mmol) and stirring for three days, with the reaction open to air. The solution was evaporated *in vacuo* to give the crude product. Azeotropic distillations were performed on the solid with toluene (3 x 50 mL) to remove residual acetic acid, and hexane (3 x 50 mL) to remove residual toluene. The solid was purified by precipitation from a chloroform solution using diethyl ether, and then dried *in vacuo* for 16 h. Single crystals suitable for X-ray diffraction experiments were grown by slow evaporation of hexane into a solution of the complex in chloroform at room temperature.

PO/CO_2 ROCOP

A solution of catalyst (0.018 mmol), *1,2*-cyclohexane diol (42 mg, 0.357 mmol), together with mesitylene (25 μL, 0.179 mmol, internal standard), in PO (5 mL, 71.5 mmol) was prepared in a N_2 glovebox. The solution was injected into a 100 mL Parr reactor, fitted with a DiComp sentinel probe attached to an ATR-IR spectrometer, under a stream of CO_2. The reactor was pressurized to 20 bar CO_2 pressure and heated to 50 °C. Conversion to polycarbonate (PPC) and cyclic carbonate (PC) were followed using *in situ* ATR-IR spectroscopy by observing the development of peaks at 1750 cm^{-1} and 1810 cm^{-1} for PPC and PC, respectively. The copolymerization was stirred for the reaction duration, allowed to cool upon completion, and quenched by addition of benzoic acid (2 mg, 0.018 mmol). Conversions to PPC, PC and polyether (PPO) were determined by ^1H NMR spectroscopy of the crude sample, using mesitylene as an internal standard, and molar masses and dispersities of resultant PPC determined by GPC in THF. The polymer product was purified by dissolving in chloroform and precipitating with methanol. Polymerizations were run in triplicate to allow determination of errors.

Epoxide/Anhydride ROCOP

A solution of catalyst (0.014 mmol) and anhydride (0.143 mmol) in epoxide (1 mL) was prepared in a dried vial, inside a N_2 glovebox. The vial was sealed, with a melamine cap containing a Teflon inlay, and further sealed with electrical insulation tape. The sealed vial was then heated to 50 °C for the time stated. Aliquoting was performed by cooling the polymerization vial in an ice/water bath, before removing ~ 10 μL of the mixture with a syringe in a N_2 glovebox. Aliquots were analysed by ^1H NMR spectroscopy ($CDCl_3$) to determine conversion, and by GPC in THF to determine the molar mass and dispersity of the resultant polyester. The polymerizations were quenched

by addition of benzoic acid (2 mg, 0.018 mmol), and the polyesters purified by precipitation from chloroform with methanol and dried under vacuum. Polymerizations were run in triplicate to allow determination of errors.

***rac*-Lactide ROP**

A solution of catalyst (0.014 mmol) and *rac*-LA (0.143 mmol) in PO (1 mL) was prepared in a dried vial inside a N_2 glovebox. The vial was sealed, with a melamine cap containing a Teflon inlay, and further sealed with electrical insulation tape. The sealed vial was then heated to 50 °C for the time stated. Aliquoting was performed by cooling the polymerization vial in an ice/water bath, before removing ~ 10 µL of the mixture with a syringe, in a N_2 glovebox. Aliquots were analysed by ^{1}H NMR spectroscopy ($CDCl_3$) to determine conversion, and by GPC in THF to determine the molar mass and dispersity of the resultant polyester. The polymerizations were quenched by addition of benzoic acid (2 mg, 0.018 mmol) and the PLA purified by precipitation from chloroform with methanol and dried under vacuum. Polymerizations were run in triplicate to allow determination of errors.

Co(III)Ca(II)

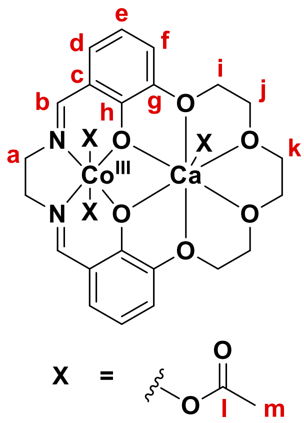

Synthesised with $Ca(OAc)_2.H_2O$. Brown solid, obtained in 0.39 g yield (0.57 mmol, 56%). ^{1}H NMR (400 MHz, 298 K, $CDCl_3$, 298 K) δ (ppm): 7.84 (s, 2H, N=C**H** (**b**)), 6.95 (t, 4H, $^3J_{H-H}$ = 8.5 Hz), *meta*-Ar**H** (**d**, **f**)), 6.60 (t, 2H, $^3J_{H-H}$ = 7.78 Hz, *para*-Ar**H** (**e**)), 3.54 – 4.87 (m, 12 H, C**H**$_2$, (**i – k**)), 4.05 (s, 4 H, NC**H**$_2$, (**a**)), 1.38 (s, 6 H, O=C-C**H**$_3$ (**m**)); ^{13}C {^{1}H} NMR (151 MHz, 298 K, $CDCl_3$) δ (ppm): 180.0 (s, O=**C** (**l**)), 165.2 (s, N=CH (**b**)), 154.6 (s, *ipso*-**C** (**h**)), 151.7 (s, *ortho*-**C** (**g**)), 127.0 (s, *meta*-**C**H (**d**)), 119.3 (s, *meta*-**C**-CH=N (**c**)), 117.7 (s, *para*-CH (**f**)), 114.9 (s, *meta*-CH (**f**)), 69.9 (O-**C**H$_2$ (**i** or **j** or **k**)), 69.7 (O-**C**H$_2$ (**i** or **j** or **k**)), 68.3 (O-**C**H$_2$ (**i** or **j** or **k**)), 59.0 (s, N-**C**H$_2$ (**a**)), 25.0 (s, O=C-**C**H$_3$ (**m**));)); ν_{max}/cm^{-1} 2926 (C-H stretch), 1647 (acetate asymmetric C-O stretch), 1580 (imine C=N stretch), 1455 (acetate symmetric C-O stretch), 1242 (aromatic C-O stretch), 1086 (aliphatic C-O stretch); Anal. Calc. for $C_{28}H_{33}CaCoN_2O_{12}$: C 48.84, H 4.83, N 4.07; Found: C 47.30, H 5.21, N 4.16 %.

Co(III)Sr(II)

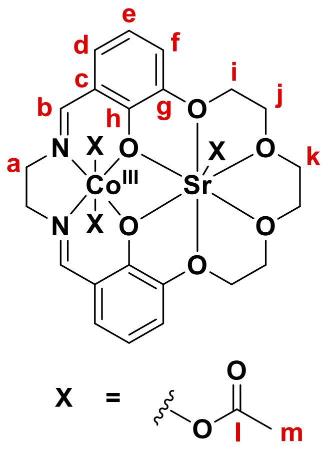

Synthesised with $Sr(OAc)_2$. Brown solid, obtained in 0.47 g yield (0.64 mmol, 63%). ^{1}H NMR (400 MHz, $CDCl_3$, 298 K) δ (ppm): 7.75 (s, 2H, N=C**H** (**b**)), 6.86 (t, 4H, $^3J_{H-H}$ = 7.9 Hz, *meta*-C**H** (**d**, **f**)), 6.53 (m, 2H, $^3J_{H-H}$ = 7.9

Hz, *para*-C**H** (**e**)), 3.52 – 4.83 (m, 12 H, C**H**$_2$ (**i – k**), 4.01 (s, 4 H, N-C**H**$_2$ (**a**)), 1.46 (s, 4H, O=C-C**H**$_3$ (**m**)); ^{13}C {^{1}H} NMR (151 MHz, 298 K, $CDCl_3$) δ (ppm): 179.6 (s, O=**C** (**l**)), 164.7 (s, N=**C**H (**b**)), 155.2 (s, *ipso*-**C** (**h**)), 152.1 (s, *ortho*-**C** (**g**)), 126.2 (s, *meta*-**C**H (**d**)), 118.9 (s, *meta*-**C**-CH=N (**c**)), 116.1 (s, *para*-**C**H (**f**)), 114.4 (s, *meta*-**C**H (**f**)), 70.6 (O-**C**H$_2$ (**i** or **j** or **k**)), 69.0 (O-**C**H$_2$ (**i** or **j** or **k**)), 68.4 (O-**C**H$_2$ (**i** or **j** or **k**)), 59.1 (s, N-**C**H$_2$ (**a**)), 25.2 (s, O=C-**C**H$_3$ (**m**)); ν_{max}/cm^{-1} 2929 (C-H stretch), 1648 (imine C=N stretch), 1585 (acetate asymmetric C-O stretch), 1455 (acetate symmetric C-O stretch), 1227 (aromatic C-O stretch), 1086 (aliphatic C-O stretch); Anal. Calc. for $C_{28}H_{33}CoN_2O_{12}Sr$: C 45.69, H 4.52, N 3.81; Found: C 45.38, H 5.04, N 3.94%.

Co(III)Ba(II)

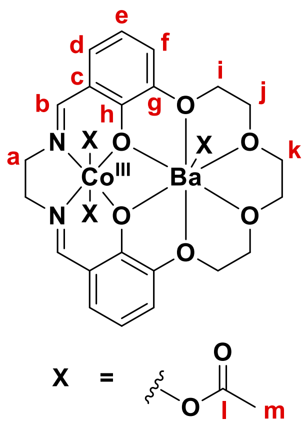

Synthesised with $Ba(OAc)_2$. Red-brown solid, obtained in 0.45 g yield (0.57 mmol, 56%). ^{1}H NMR (400 MHz, $CDCl_3$, 298 K) δ (ppm): 7.68 (s, 2H, N=C**H** (**b**)), 6.83 (d, 4H, $^3J_{H-H}$ = 7.6 Hz, *meta*-C**H** (**d**, **f**)), 6.50 (t, 2H, $^3J_{H-H}$ = 7.8 Hz, *para*-C**H** (**e**)),)), 3.75 – 4.61 (m, 12 H, C**H**$_2$ (**i – k**), 4.03 (s, 4 H, N-C**H**$_2$ (**a**)), 1.44 (s, 4H, O=C-C**H**$_3$ (**m**)); ^{13}C {^{1}H} NMR (151 MHz, 298 K, $CDCl_3$) δ (ppm): 179.0 (s, O=**C** (**l**)), 164.7 (s, N=**C**H (**b**)), 155.5 (s, *ipso*-**C** (**h**)), 152.2 (s, *ortho*-**C** (**g**)), 126.4 (s, *meta*-**C**H (**d**)), 118.9 (s, *meta*-**C**-CH=N (**c**)), 115.3 (s, *para*-**C**H (**f**)), 114.1 (s, *meta*-CH (**f**)), 70.6 (O-**C**H$_2$ (**i** or **j** or **k**)), 69.3 (O-**C**H$_2$ (**i** or **j** or **k**)), 67.9 (O-**C**H$_2$ (**i** or **j** or **k**)), 5921 (s, N-**C**H$_2$ (**a**)), 25.0 (s, O=C-**C**H$_3$ (**m**)); ν_{max}/cm^{-1} 3045 (C-H stretch), 2934 (C-H stretch), 1647 (imine C=N stretch), 1575 (acetate asymmetric C-O stretch), 1451 (acetate symmetric C-O stretch), 1225 (aromatic C-O stretch), 1084 (aliphatic C-O stretch); Anal. Calc. for $C_{28}H_{33}BaCoN_2O_{12}$: C 42.80, H 4.23, N 3.56; Found: C 42.18, H 4.15, N 3.34 %.

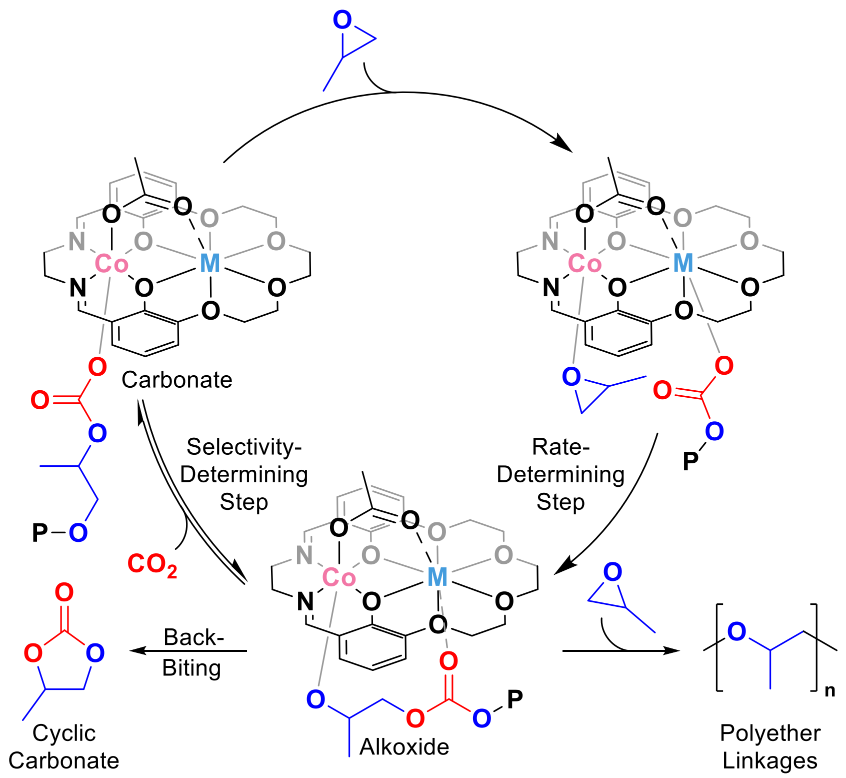

Supplementary Fig 1. Dinuclear metallate mechanism for PO/CO_2 ROCOP catalysed by Co(III)K(I) catalyst.

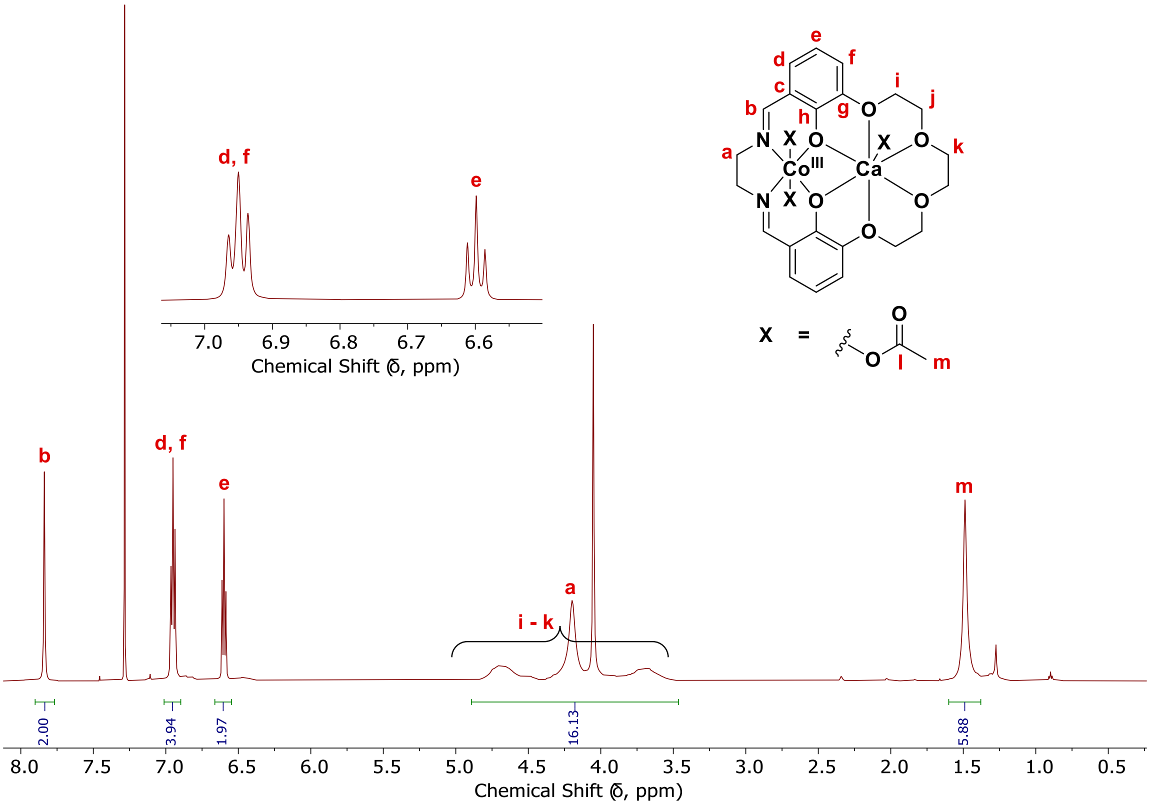

Supplementary Fig 2. ^{1}H NMR spectrum of Co(III)Ca(II).

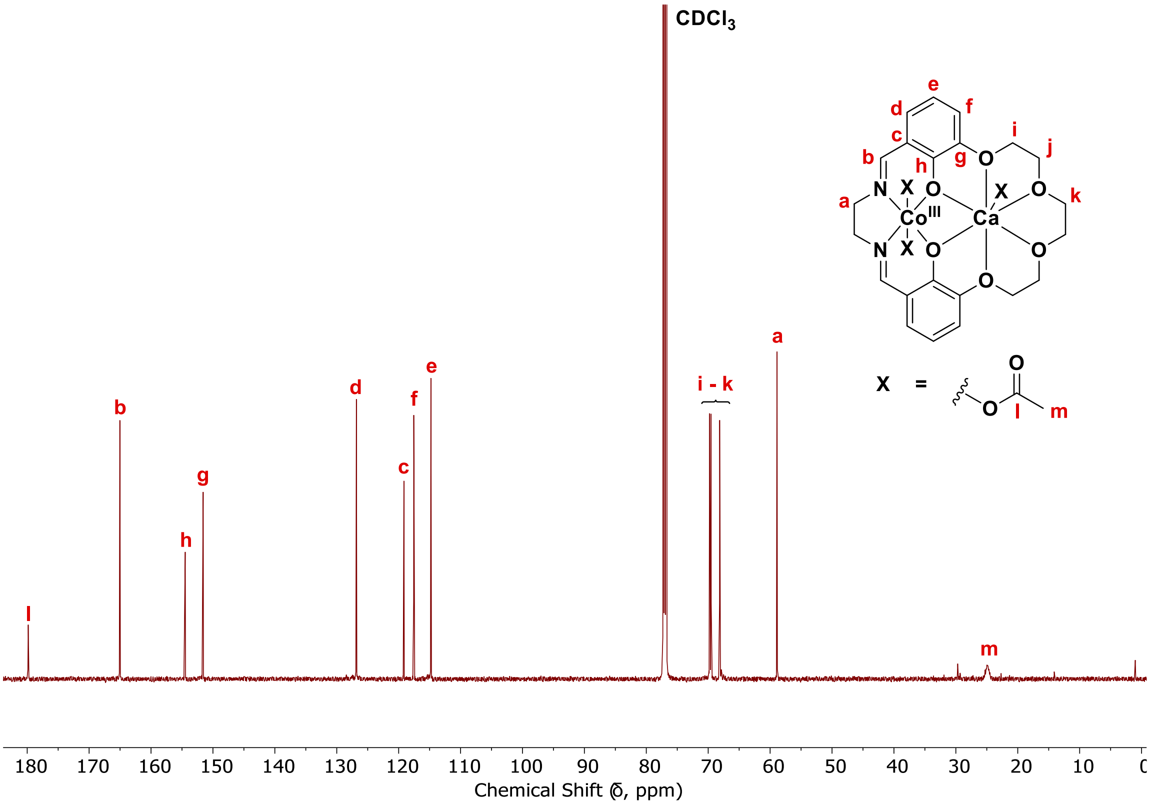

Supplementary Fig 3. $^{13}C\{^1H\}$ NMR spectrum of Co(III)Ca(II).

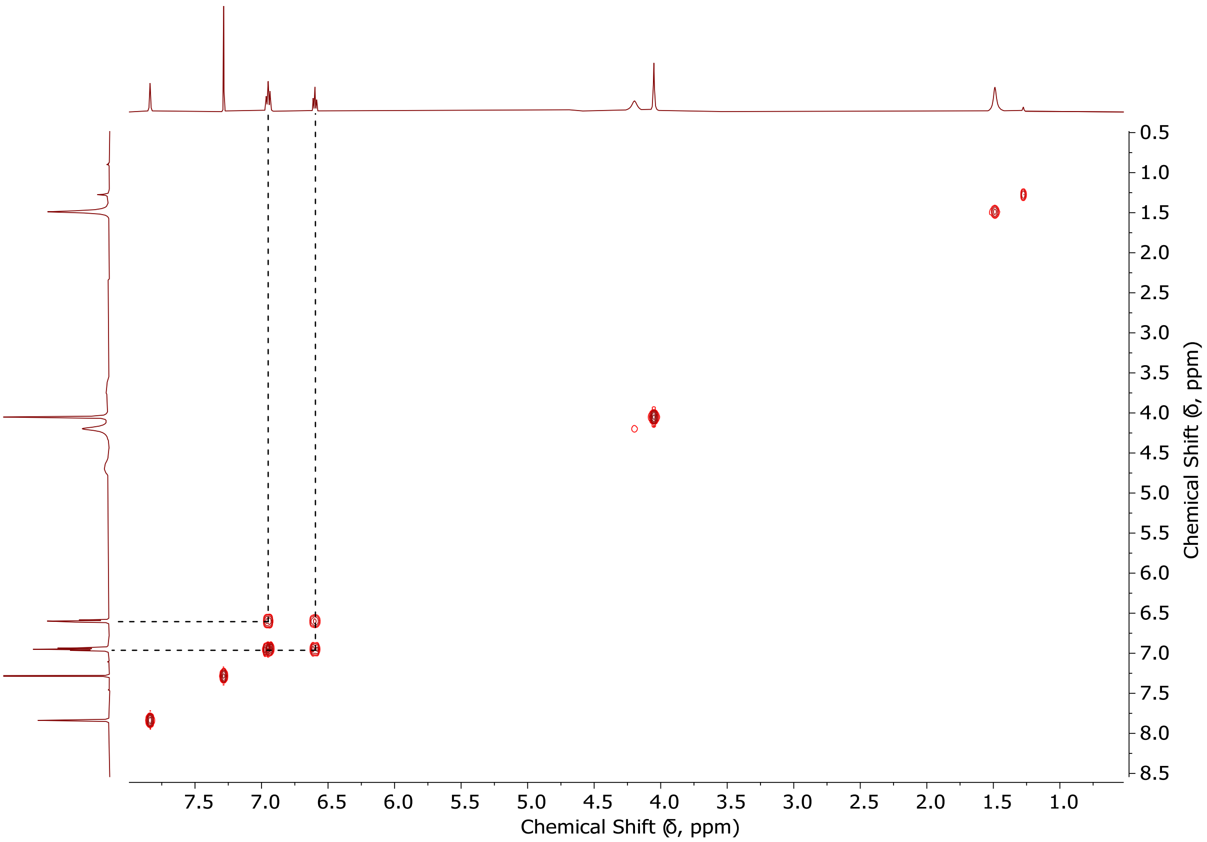

Supplementary Fig 4. COSY NMR spectrum of Co(III)Ca(II).

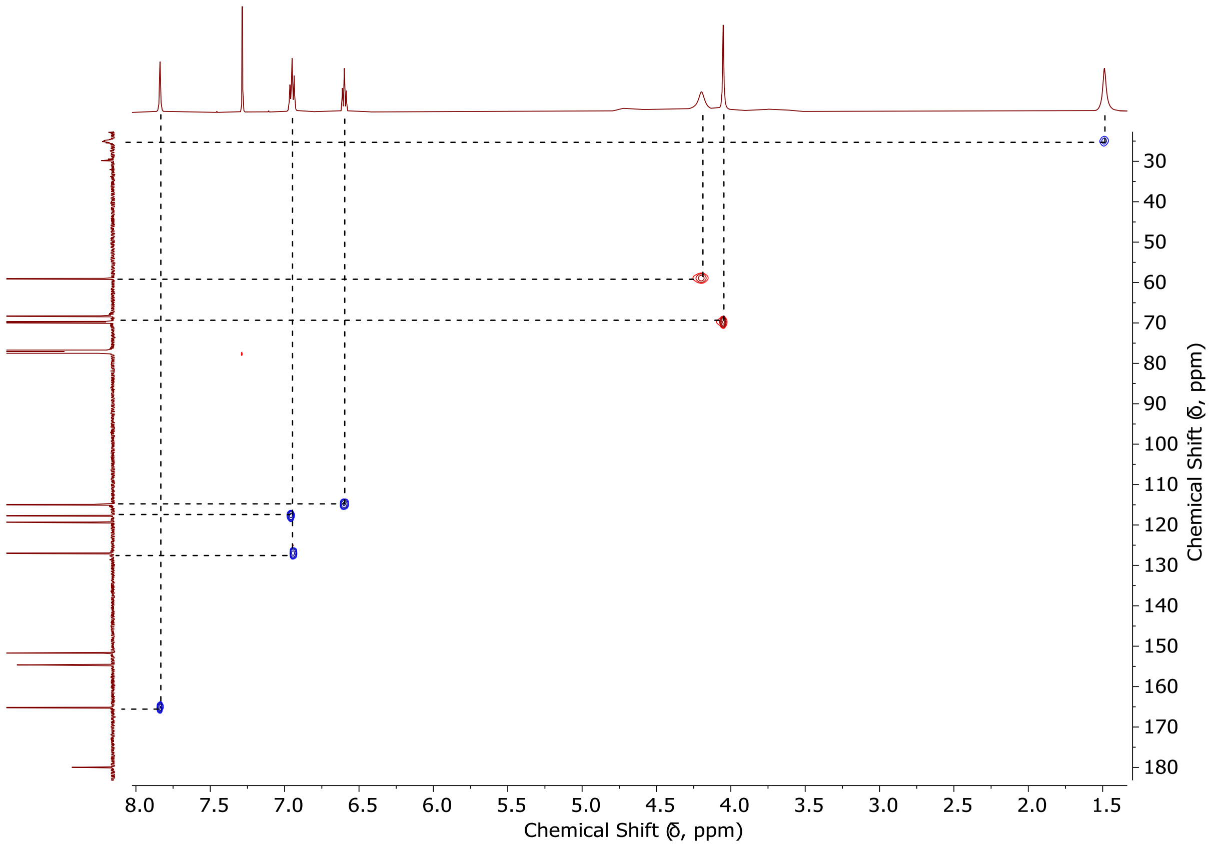

Supplementary Fig 5. HSQC NMR spectrum of Co(III)Ca(II).

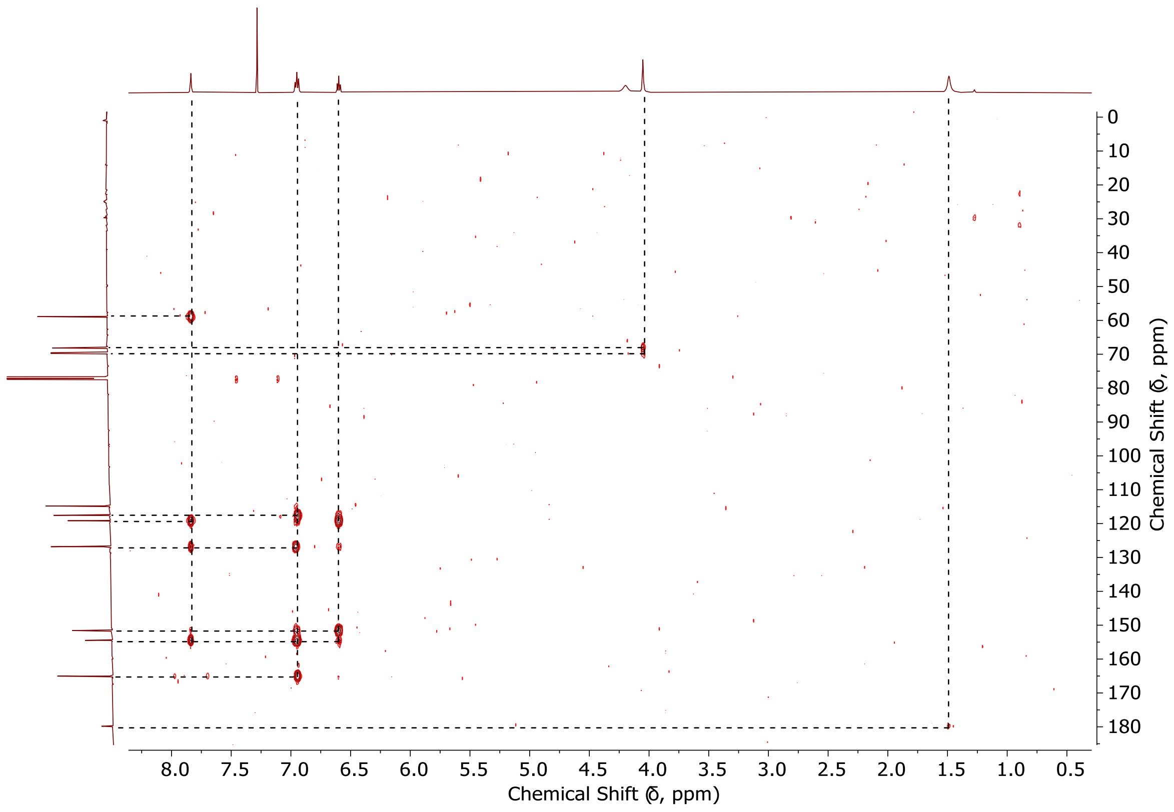

Supplementary Fig 6. HMBC NMR spectrum of Co(III)Ca(II).

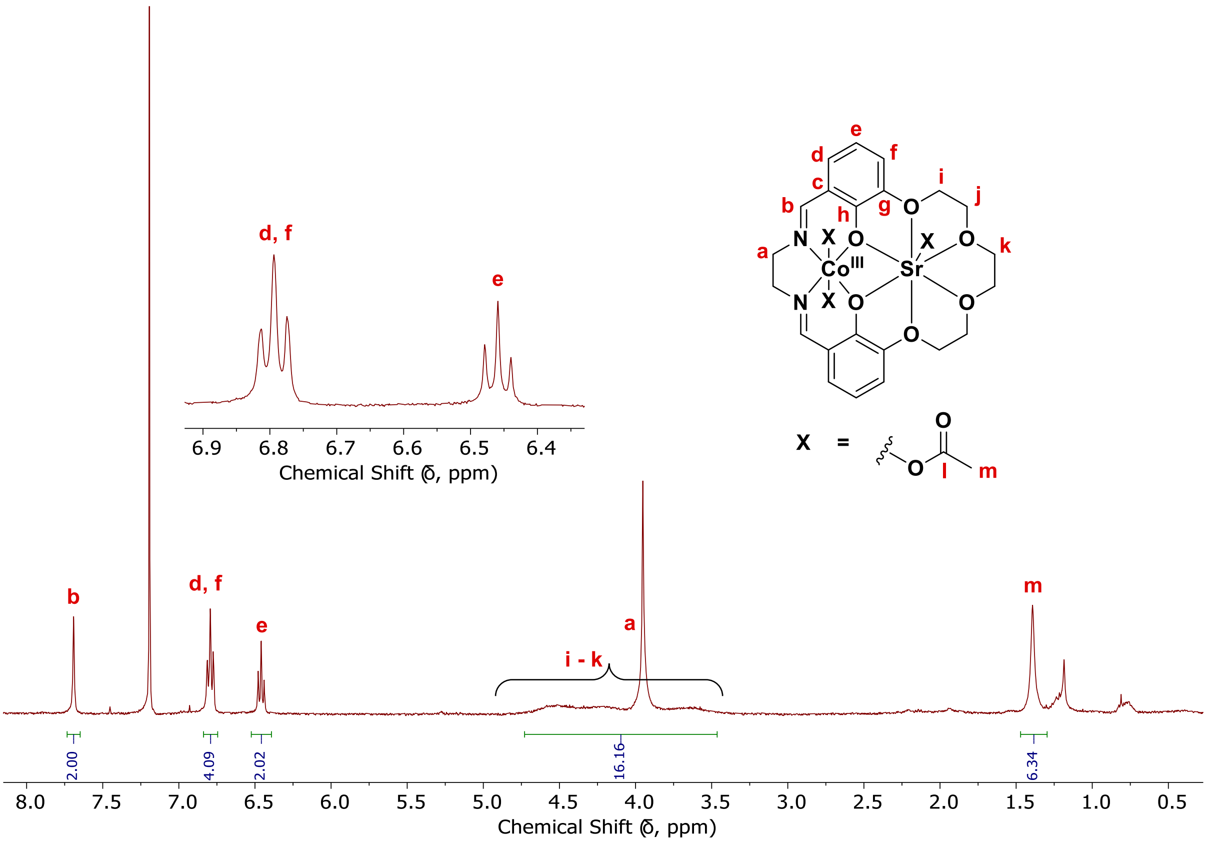

Supplementary Fig 7. ^{1}H NMR spectrum of Co(III)Sr(II).

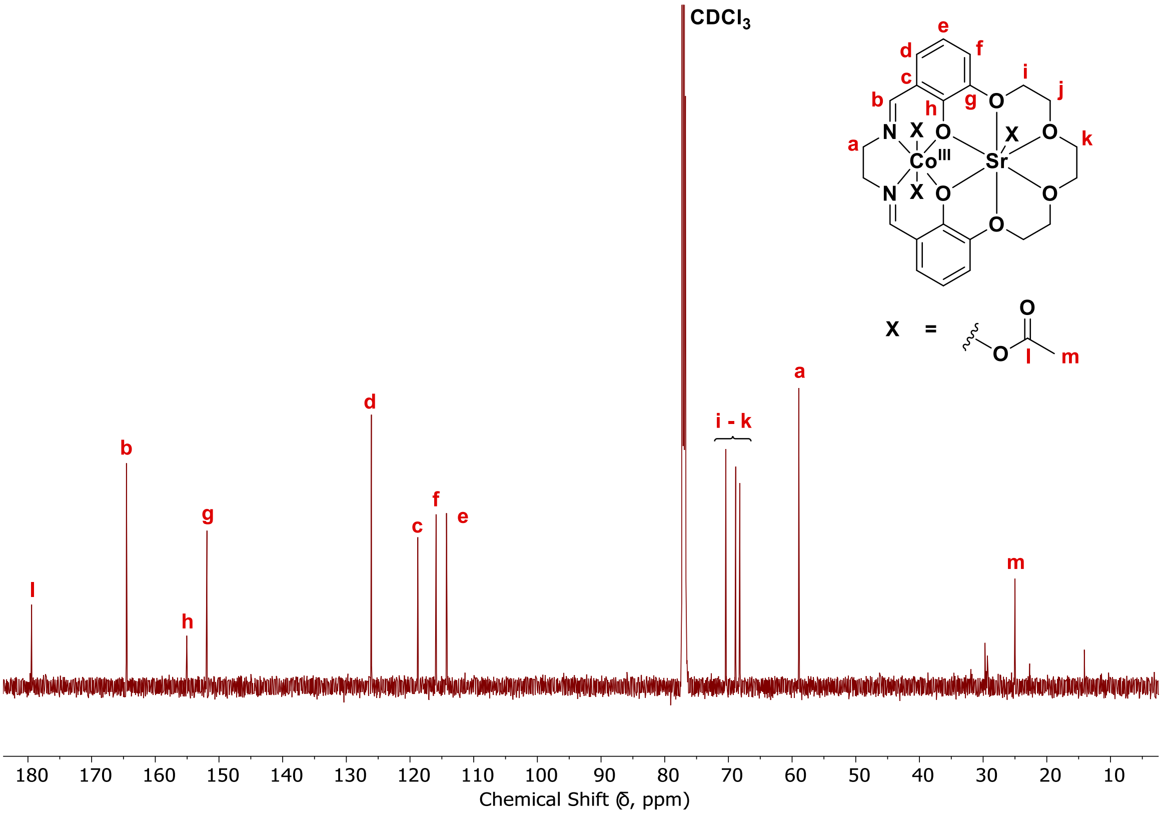

Supplementary Fig 8. ^{13}C{^{1}H} NMR spectrum of Co(III)Sr(II).

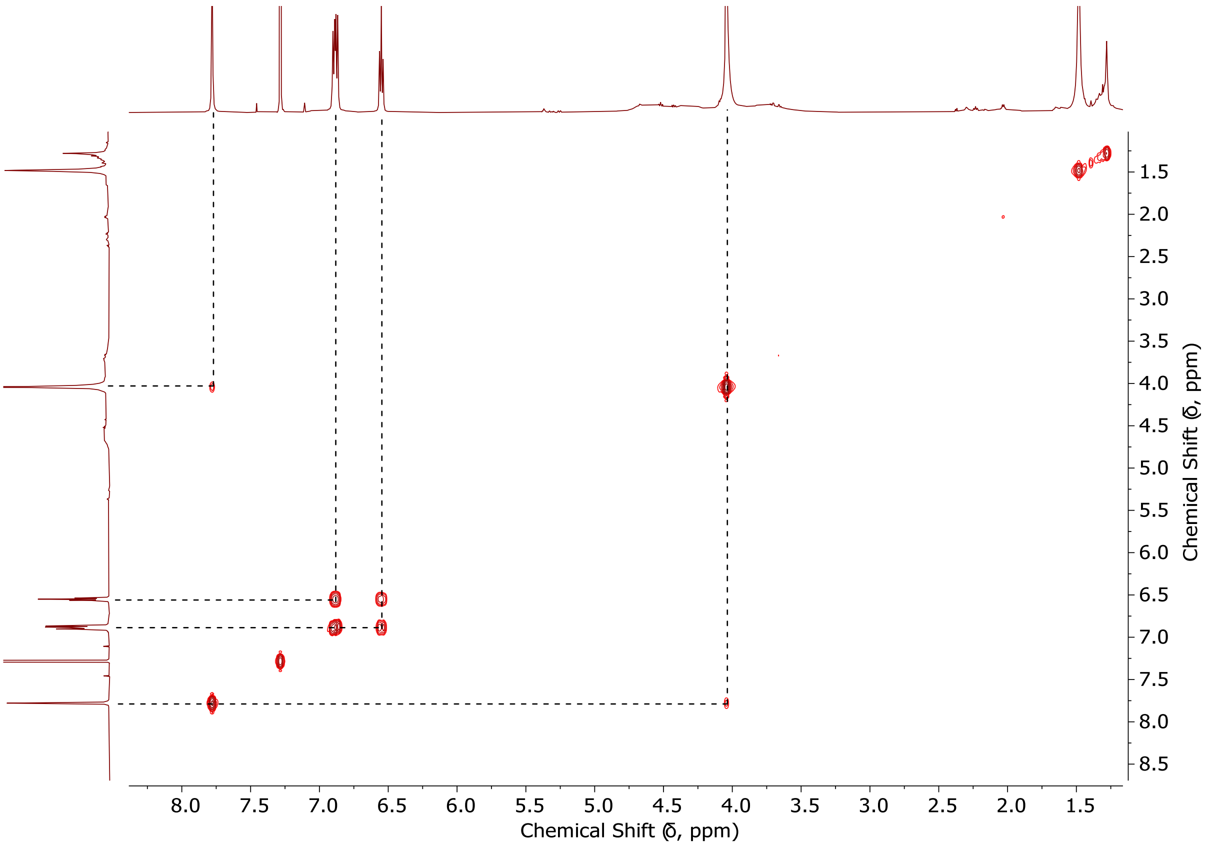

Supplementary Fig 9. COSY NMR spectrum of Co(III)Sr(II).

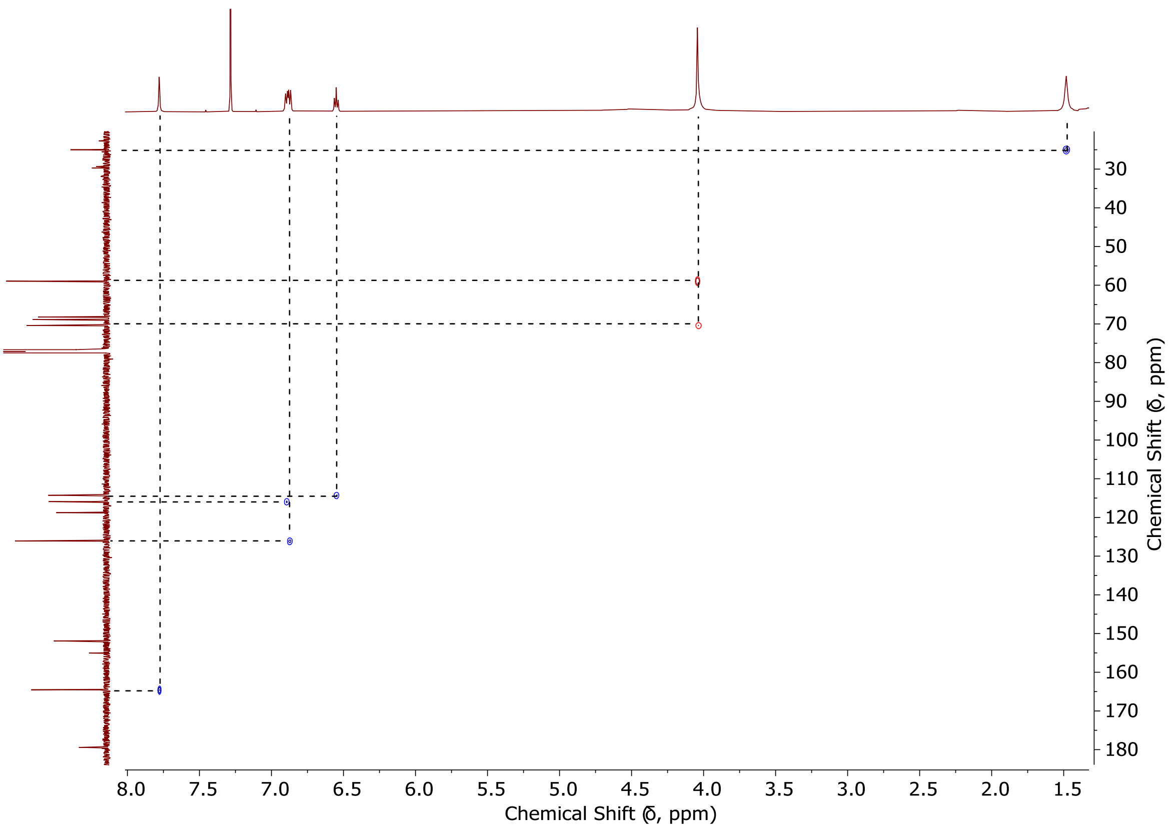

Supplementary Fig 10. HSQC NMR spectrum of Co(III)Sr(II).

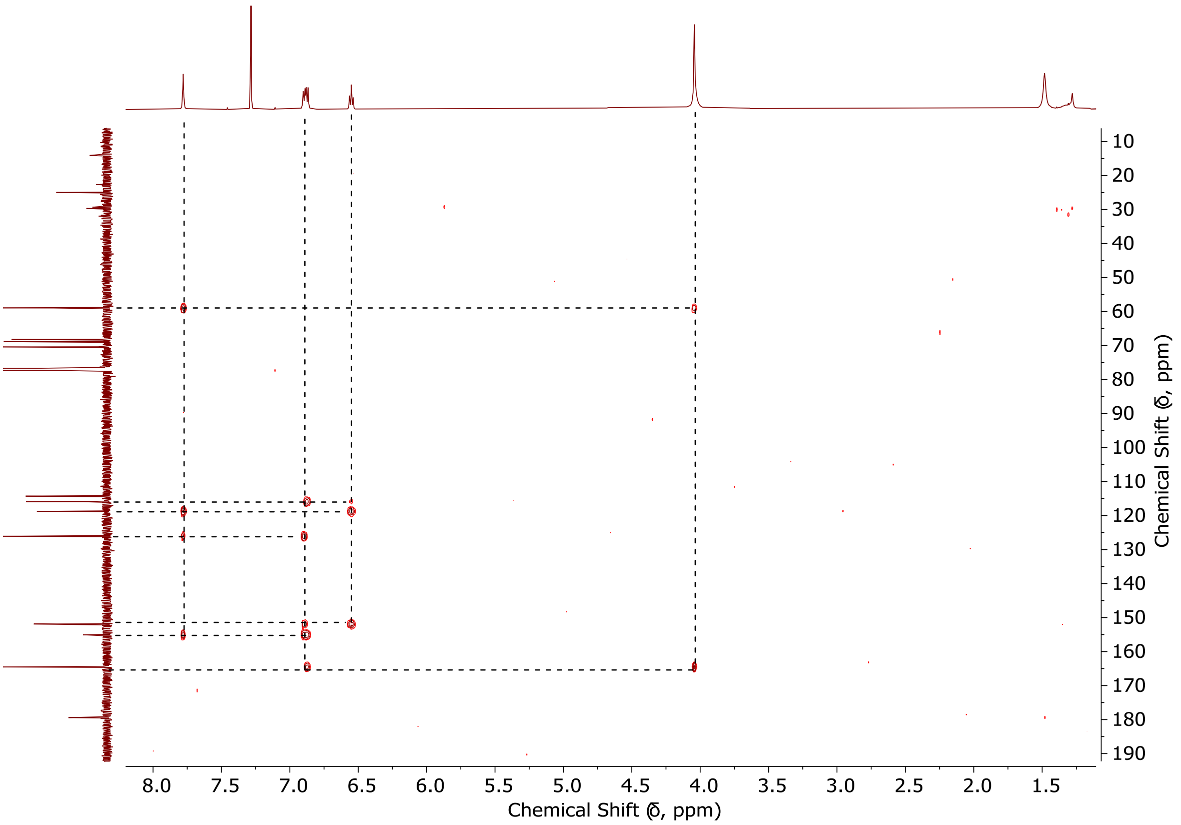

Supplementary Fig 11. HMBC NMR spectrum of Co(III)Sr(II).

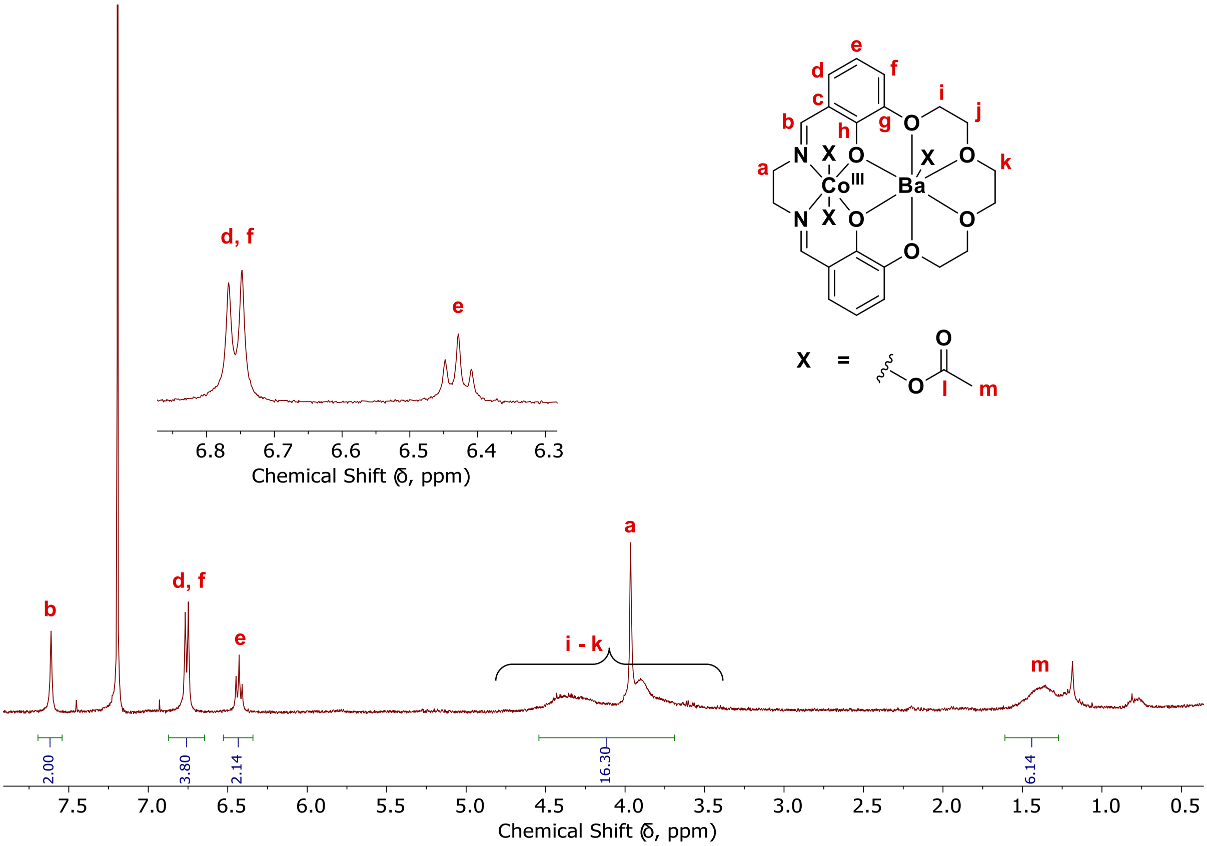

Supplementary Fig 12. ^{1}H NMR spectrum of Co(III)Ba(II).

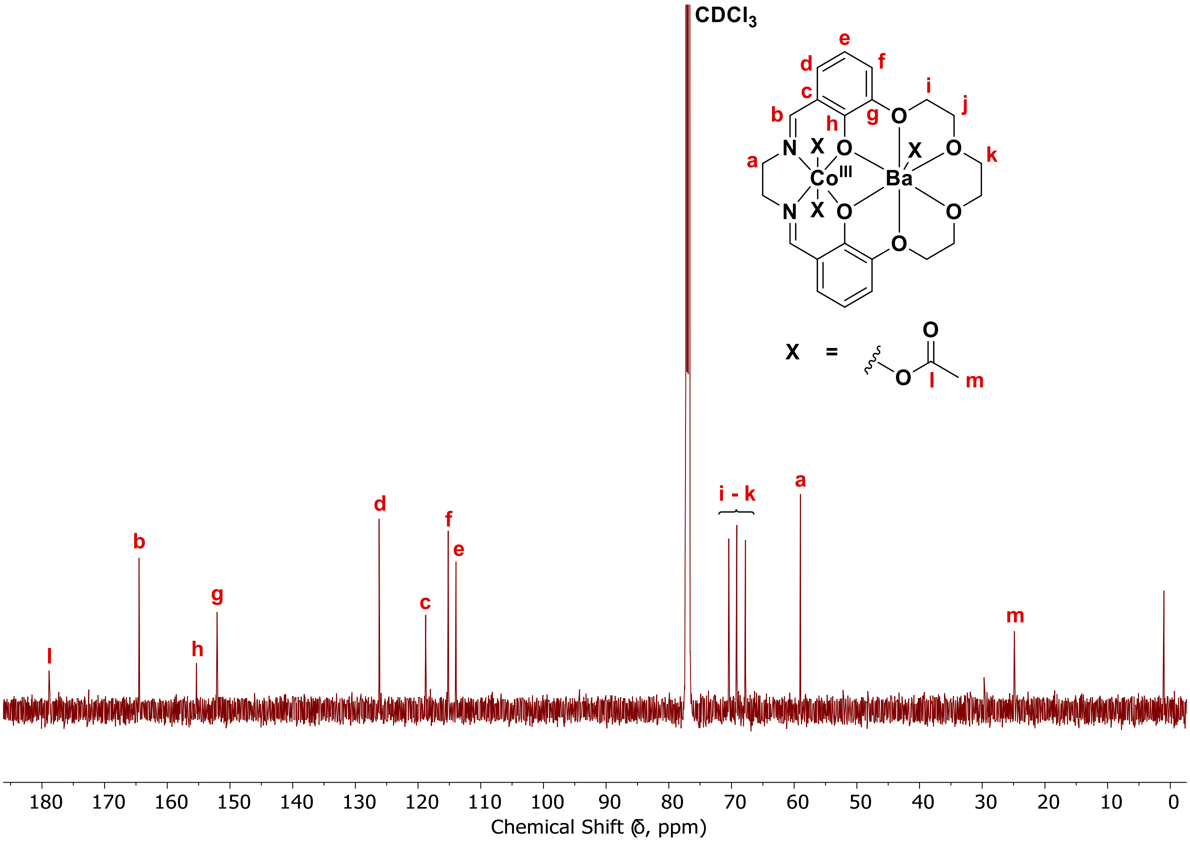

Supplementary Fig 13. $^{13}C\{^{1}H\}$ NMR spectrum of Co(III)Ba(II).

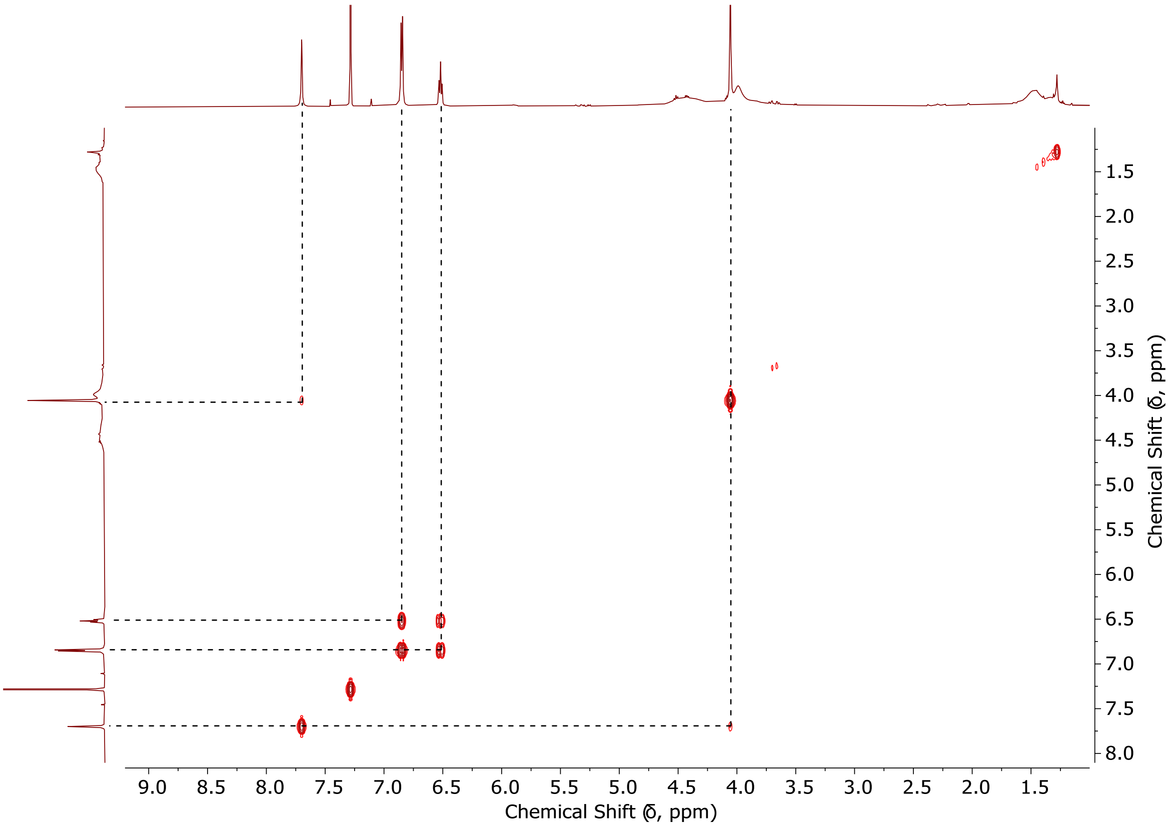

Supplementary Fig 14. COSY NMR spectrum of Co(III)Ba(II).

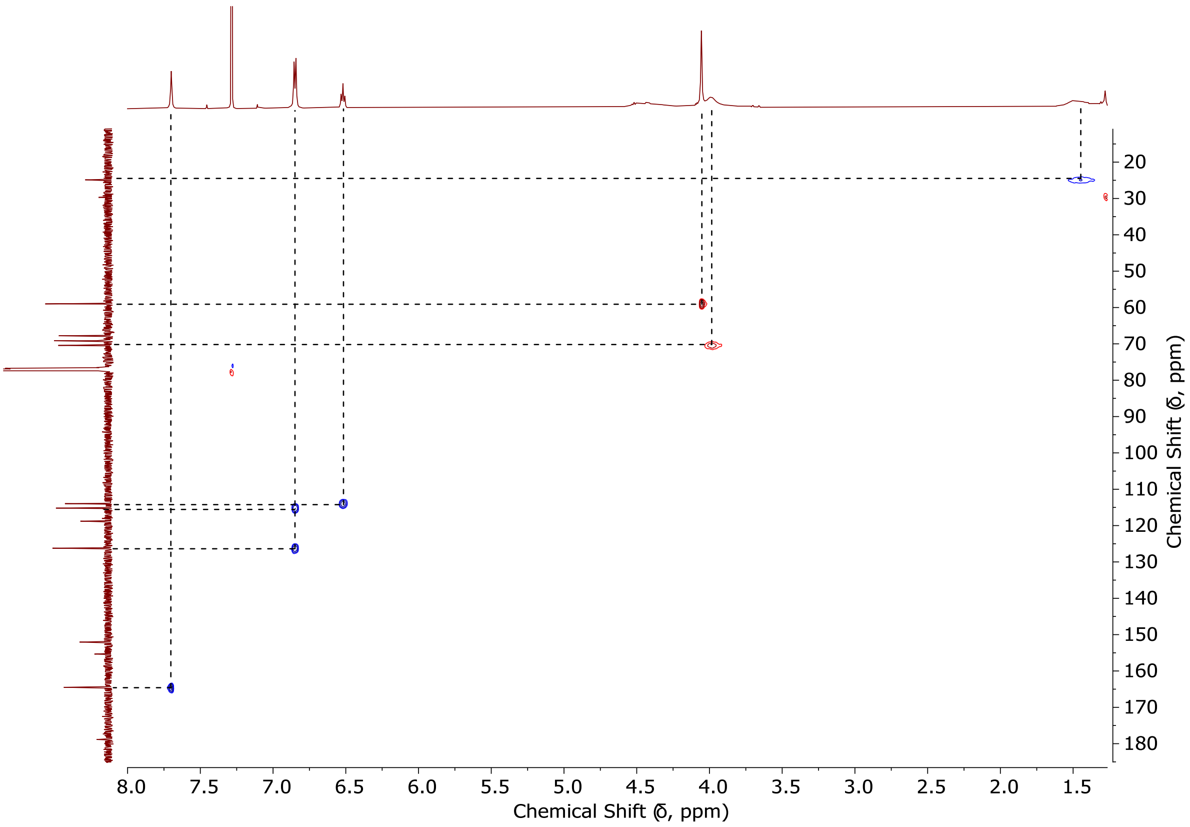

Supplementary Fig 15. HSQC NMR spectrum of Co(III)Ba(II).

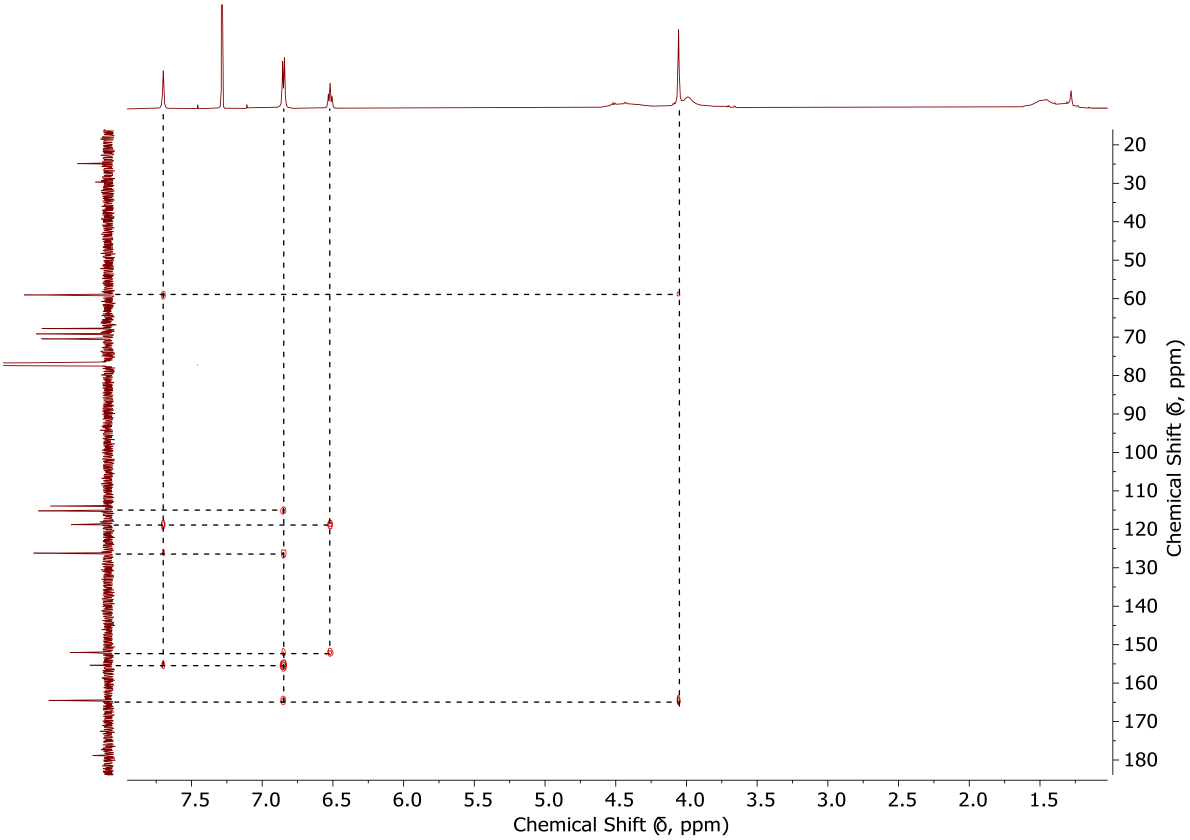

Supplementary Fig 16. HMBC NMR spectrum of Co(III)Ba(II).

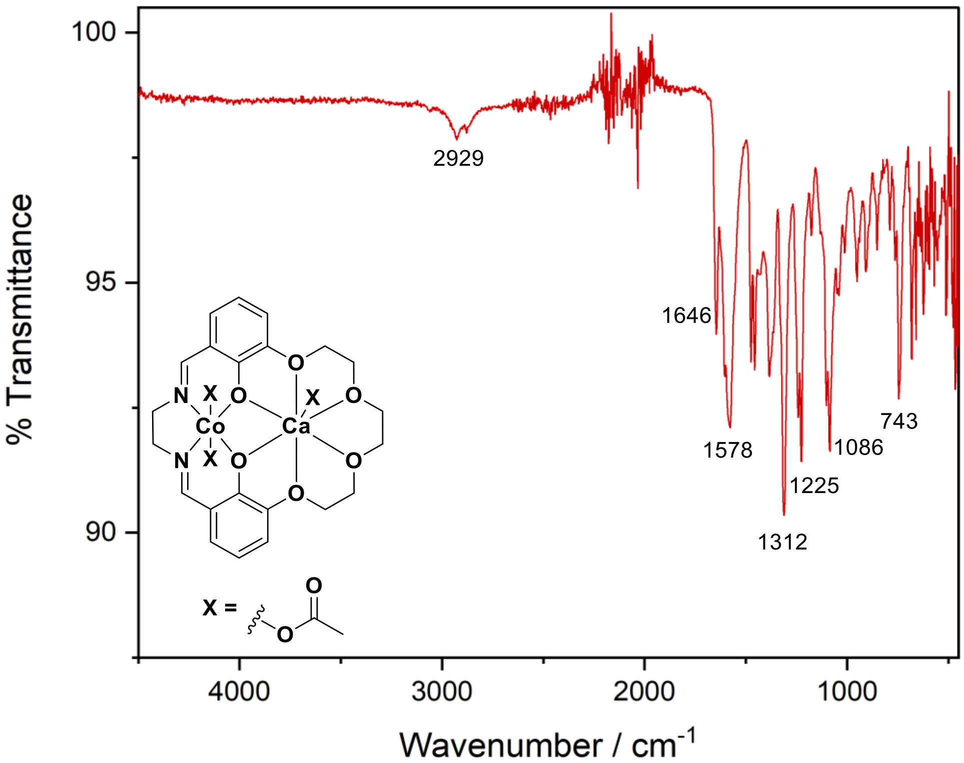

Supplementary Fig 17. IR spectrum of Co(III)Ca(II).

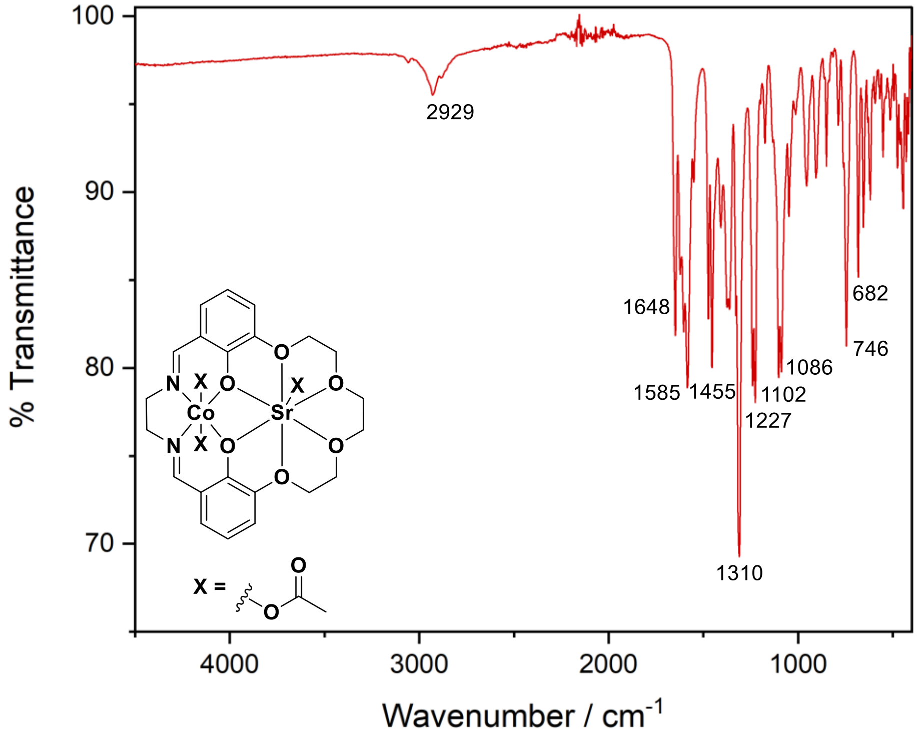

Supplementary Fig 18. IR spectrum of Co(III)Sr(II).

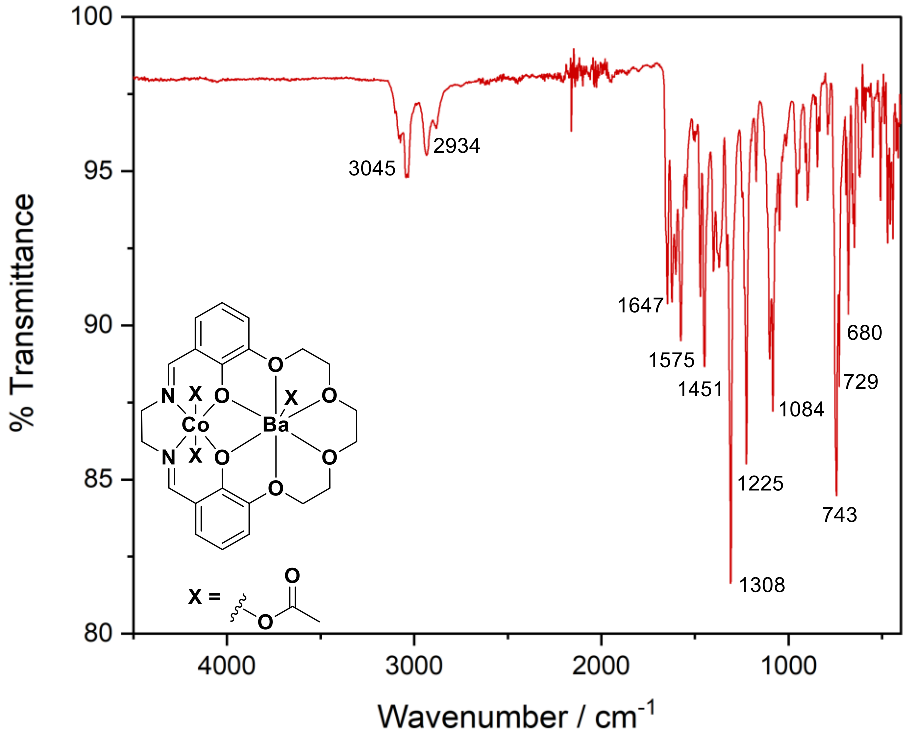

Supplementary Fig 19. IR spectrum of Co(III)Ba(II).

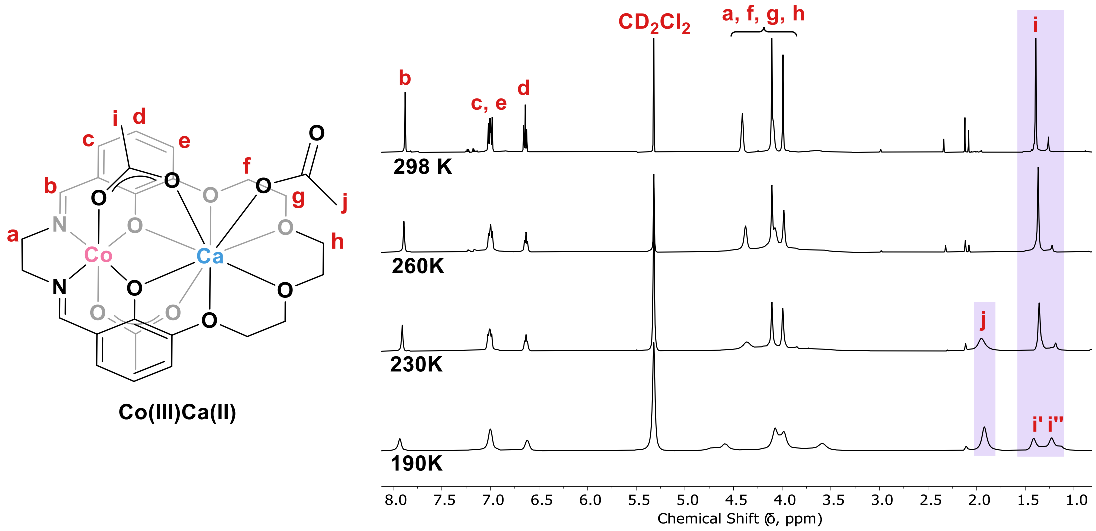

Supplementary Fig 20. Variable temperature (VT) spectrum of Co(III)Ca(II) showing the development of a new acetate peak (j) at 230 K and the splitting of the original acetate peak into two at 190 K (i', i'').

The Co(III)M(II) complexes show room temperature ^{1}H NMR spectra with relative integrals of six protons, rather than the expected nine, for the acetate ligands, but at low temperatures an additional acetate resonance, at 1.92 ppm (**j**; s, 3H) corresponding to a terminally coordinated acetate, is observed (Supplementary Fig 20). At lower temperatures the two bridging acetate ligands are inequivalent (**i'**; 1.41 ppm, s, 3H and **ii'**;1.23 ppm, s, 3H) which is fully consistent with the solid state structure determined by X-ray diffraction experiments for Co(III)Ca(II) (Supplementary Fig 21).

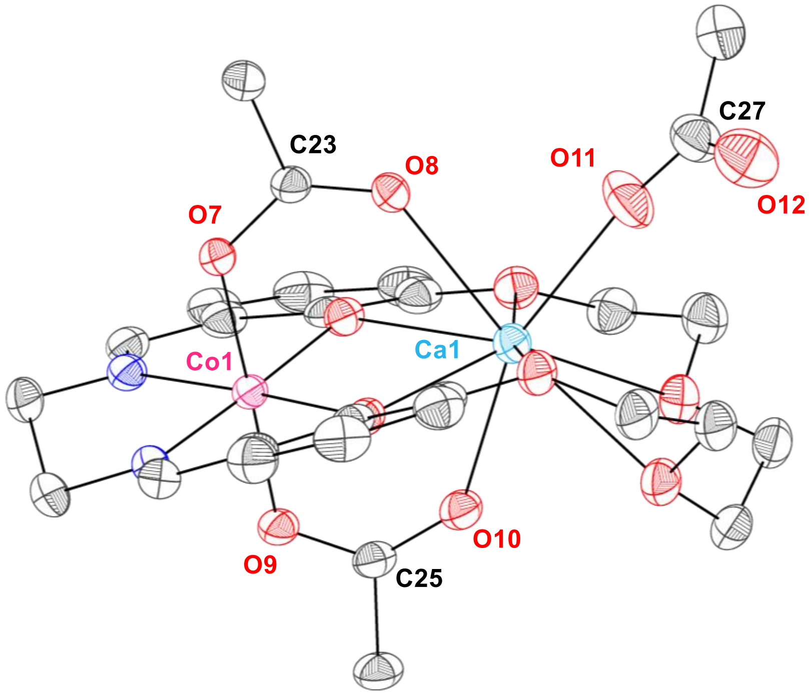

Supplementary Fig 21. Molecular structure of Co(III)Ca(II), determined by X-ray diffraction, with hydrogen atoms and solvent omitted for clarity. Thermal ellipsoids are represented at 40% probability. Selected bond lengths and angles are listed in Supplementary Tab 10 and 11 respectively.

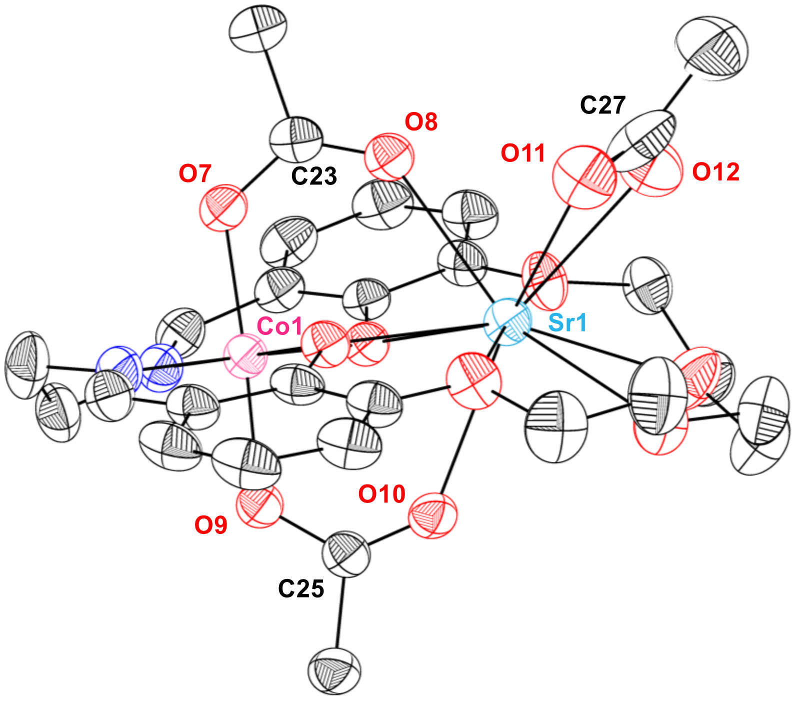

Supplementary Fig 22: Molecular structure of Co(III)Sr(II), determined by X-ray diffraction, with hydrogen atoms and solvent omitted for clarity. Thermal ellipsoids are represented at 40% probability. Selected bond lengths and angles are listed in Supplementary Tab 10 and 11 respectively.

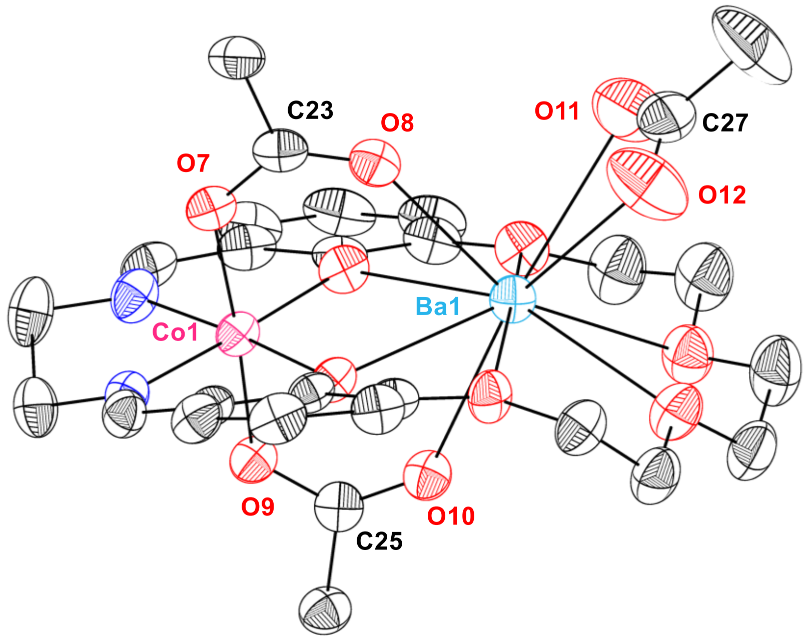

Supplementary Fig 23. Molecular structure of Co(III)Ba(II), determined by X-ray diffraction, with hydrogen atoms and solvent omitted for clarity. Thermal ellipsoids are represented at 40% probability. Selected bond lengths and angles are listed in Supplementary Tab 10 and 11 respectively.

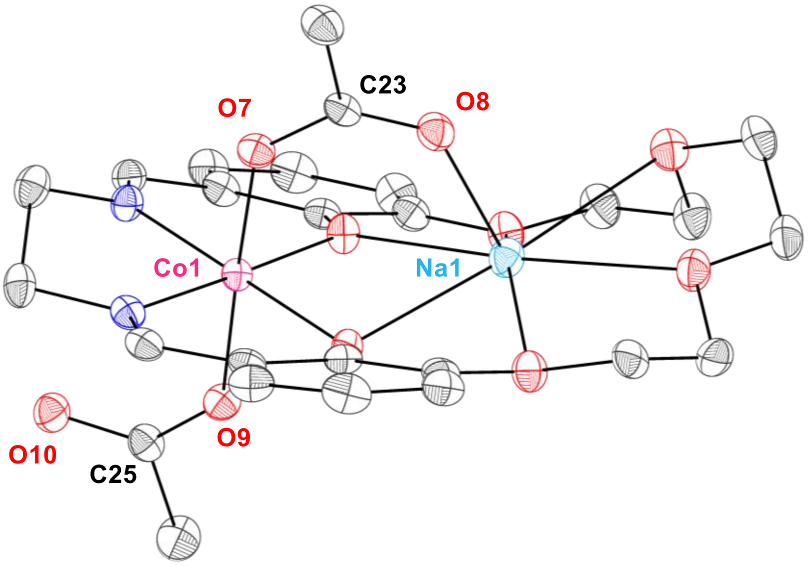

Supplementary Fig 24: Molecular structure of Co(III)Na(I), determined by X-ray diffraction, with hydrogen atoms and solvent omitted for clarity. Thermal ellipsoids are represented at 40% probability.[2]

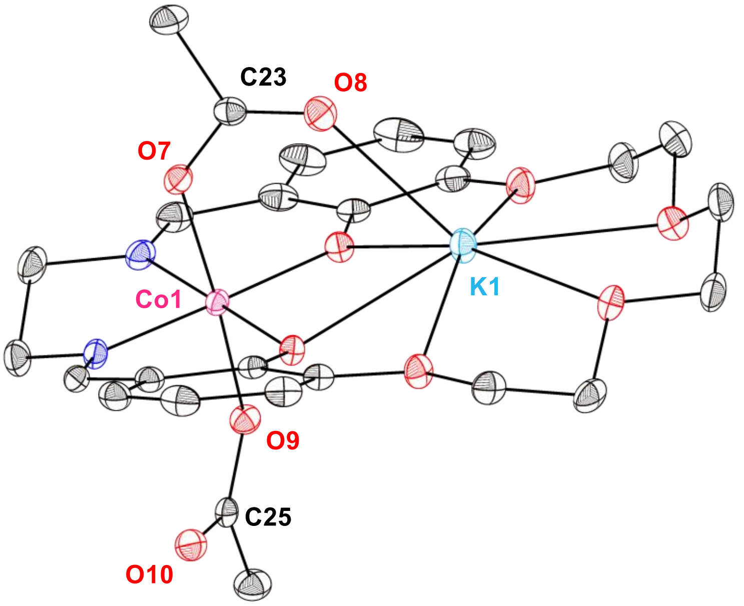

Supplementary Fig 25 Molecular structure of Co(III)K(I), determined by X-ray diffraction, with hydrogen atoms and solvent omitted for clarity. Thermal ellipsoids are represented at 40% probability.[2]

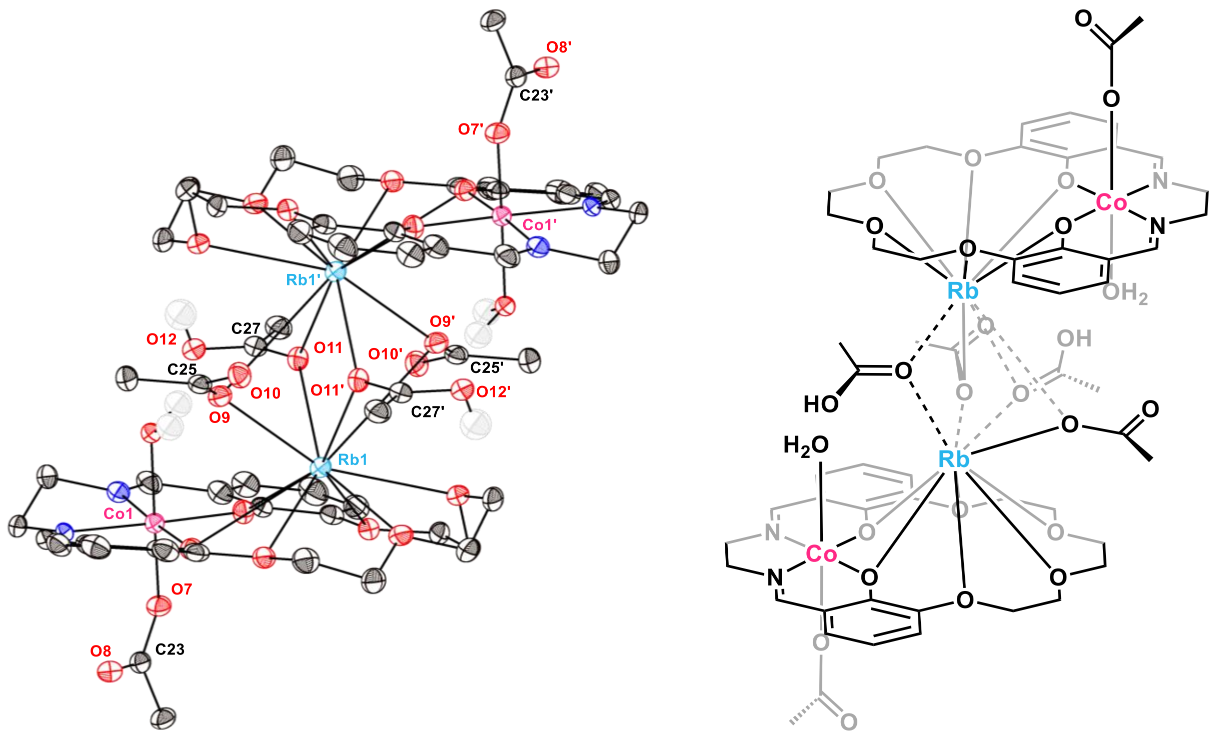

Supplementary Fig 26: A. Molecular structure of Co(III)Rb(I), determined by X-ray diffraction, with selected hydrogen atoms and outer sphere solvent omitted for clarity. Thermal ellipsoids are represented at 20% probability; B. Illustrated dimeric structure of Co(III)Rb(I) crystal structure for clarity.[2]

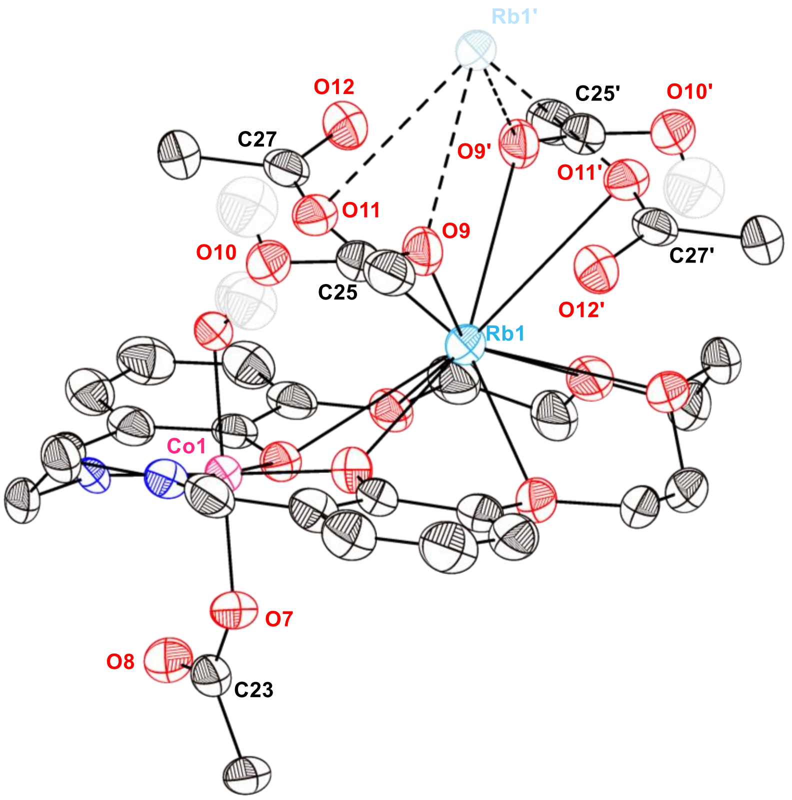

Supplementary Fig 27. Molecular structure of Co(III)Rb(I) half-dimer, determined by X-ray diffraction, with selected hydrogen atoms and outer sphere solvent omitted for clarity. Thermal ellipsoids are represented at 40% probability.[2]

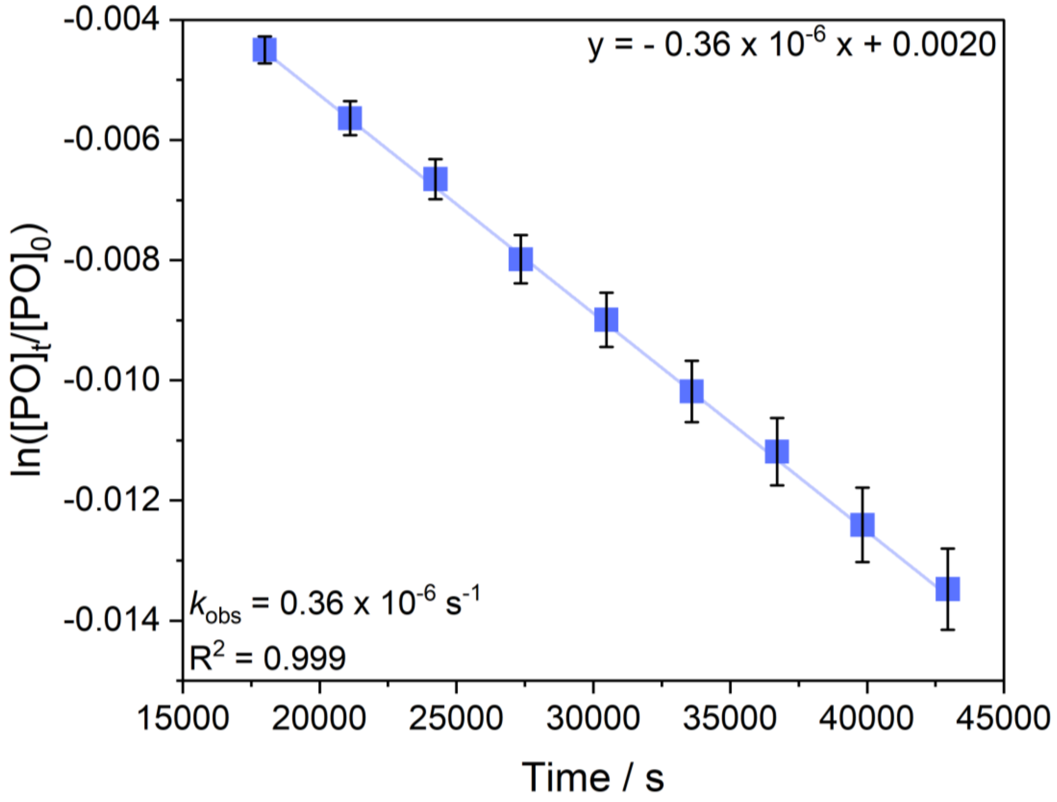

Supplementary Fig 28: Plot of ln($[PO]_t/[PO]_0$) against time for PO/CO_2 ROCOP catalysed by Co(III)Ca(II) to calculate k_{obs}. (Every 52nd point shown).

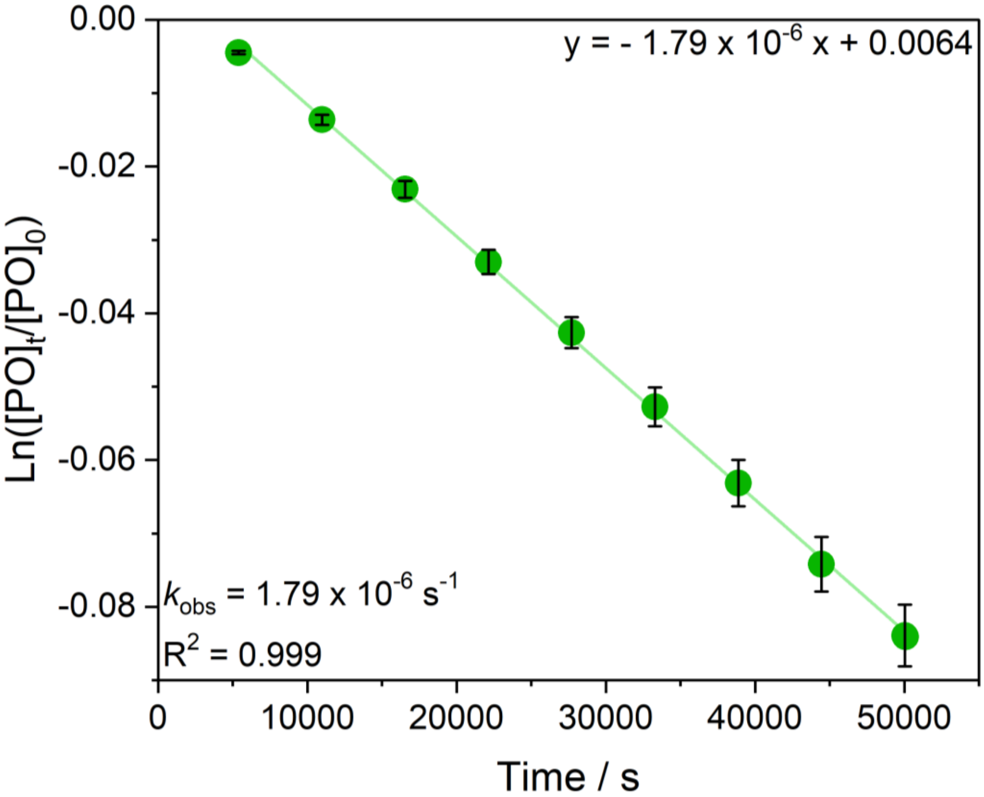

Supplementary Fig 29: Plot of ln($[PO]_t/[PO]_0$) against time for PO/CO_2 ROCOP catalysed by Co(III)Sr(II) to calculate k_{obs}. (Every 75th point shown)

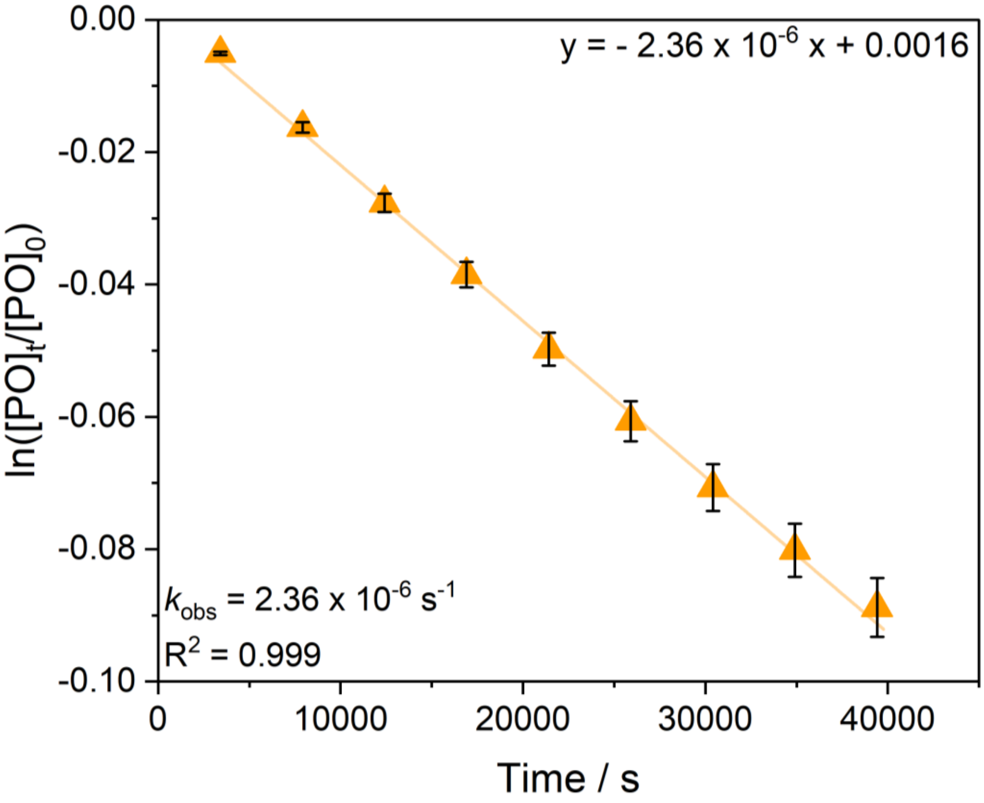

Supplementary Fig 30: Plot of ln($[PO]_t/[PO]_0$) against time for PO/CO_2 ROCOP catalysed by Co(III)Ba(II) to calculate k_{obs}. (Every 75th point shown).

Supplementary Tab 1: Values for derivation of supplementary equation 1.

Conversion / %	TON	$[PO]_t$ / M	$\ln([PO]_t/[PO]_0)$
5	200	13.59	- 0.0513
20	800	12.14	- 0.2231

Supplementary Tab 2: Description of parameters used for the derivation of supplementary equation 1.

Parameters	Description
$[PO]_t$	Concentration of PO at time *t*
$[PO]_0$	Starting concentration of PO (i.e. at time = 0 s)
k_{obs}	Observed rate coefficient
t	Time
t_i	Initiation time
$TOF_{5\text{-}20\%}$	Turnover frequency between 5 and 20% conversion
$TON_{20\%}$	Turnover number at 20% conversion
$TON_{5\%}$	Turnover number at 5% conversion
$t_{20\%}$	Time at 20% conversion
$t_{5\%}$	Time at 5% conversion

Derivation of Supplementary Equation 1:

$$\ln\frac{[\mathrm{PO}]_\mathrm{t}}{[\mathrm{PO}]_0} = -k_{\mathrm{obs}}t + t_i$$

$$\mathrm{TOF}_{5-20\%} = \frac{\mathrm{TON}_{20\%} - \mathrm{TON}_{5\%}}{t_{20\%} - t_{5\%}} = \frac{800 - 200}{t_{20\%} - t_{5\%}} = \frac{600}{t_{20\%} - t_{5\%}}$$

$$\mathrm{t} = \frac{\ln\frac{[\mathrm{PO}]_\mathrm{t}}{[\mathrm{PO}]_0} - t_i}{-k_{\mathrm{obs}}}$$

$$t_{5\%} = \frac{0.05 - t_i}{-k_{\mathrm{obs}}} \quad t_{20\%} = \frac{0.22 - t_i}{-k_{\mathrm{obs}}}$$

$$t_{20\%} - t_{5\%} = \frac{0.22 - t_i}{-k_{\mathrm{obs}}} - \frac{0.05 - t_i}{-k_{\mathrm{obs}}} = \frac{0.17}{k_{\mathrm{obs}}}$$

$$\mathrm{TOF}_{5-20\%} = \frac{600 \times k_{\mathrm{obs}} \times 3600\ \mathrm{h}^{-1}}{0.17} = \boldsymbol{k_{\mathrm{obs}} \times 12.71 \times 10^{6}\ \mathrm{h}^{-1}} \qquad \text{Eq. 1}$$

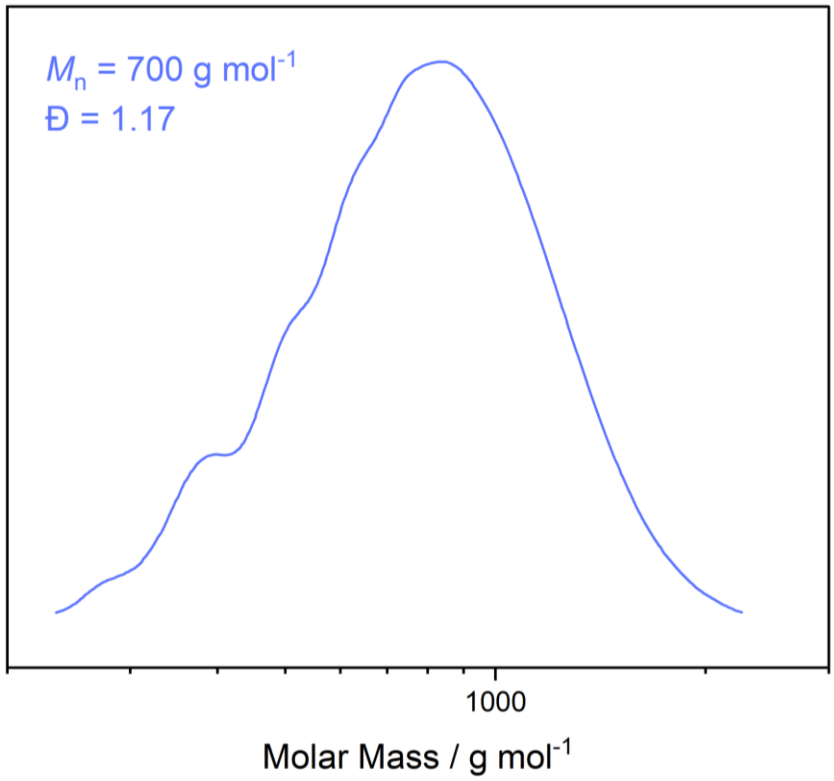

Supplementary Fig 31. GPC trace for PO/CO_2 polymerisation with Co(III)Ca(II) in THF, using narrow dispersity polystyrene standards.

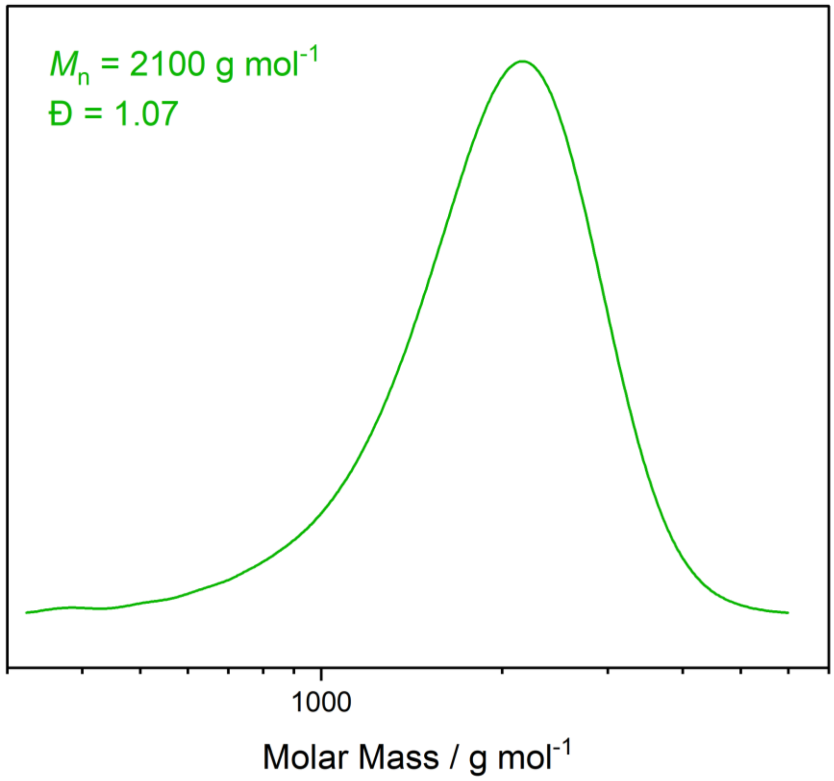

Supplementary Fig 32. GPC trace for PO/CO_2 polymerisation with Co(III)Sr(II) in THF, using narrow dispersity polystyrene standards.

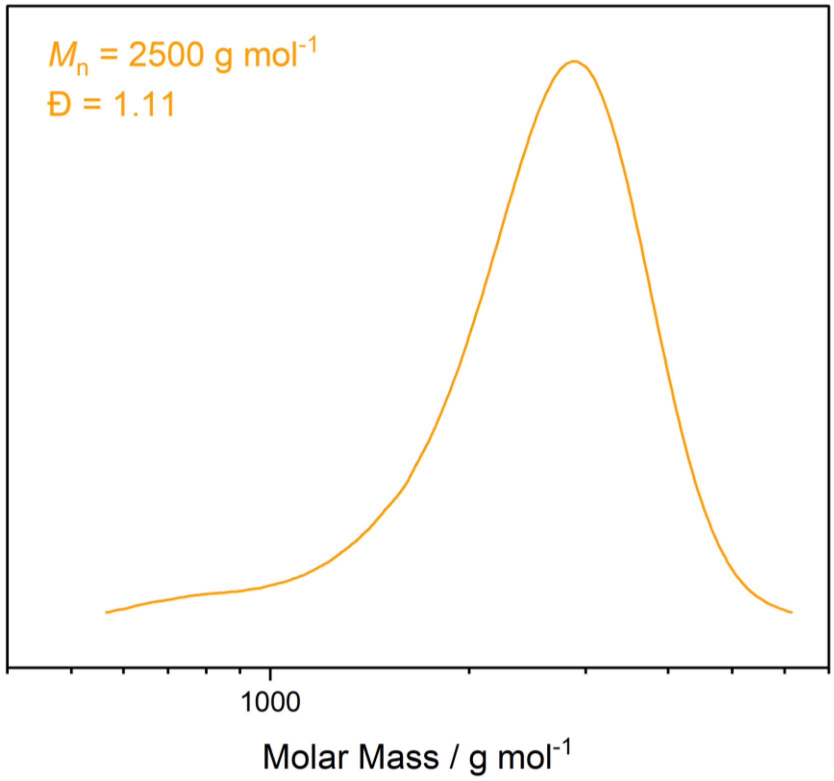

Supplementary Fig 33. GPC trace for PO/CO_2 polymerisation with Co(III)Ba(II) in THF, using narrow dispersity polystyrene standards.

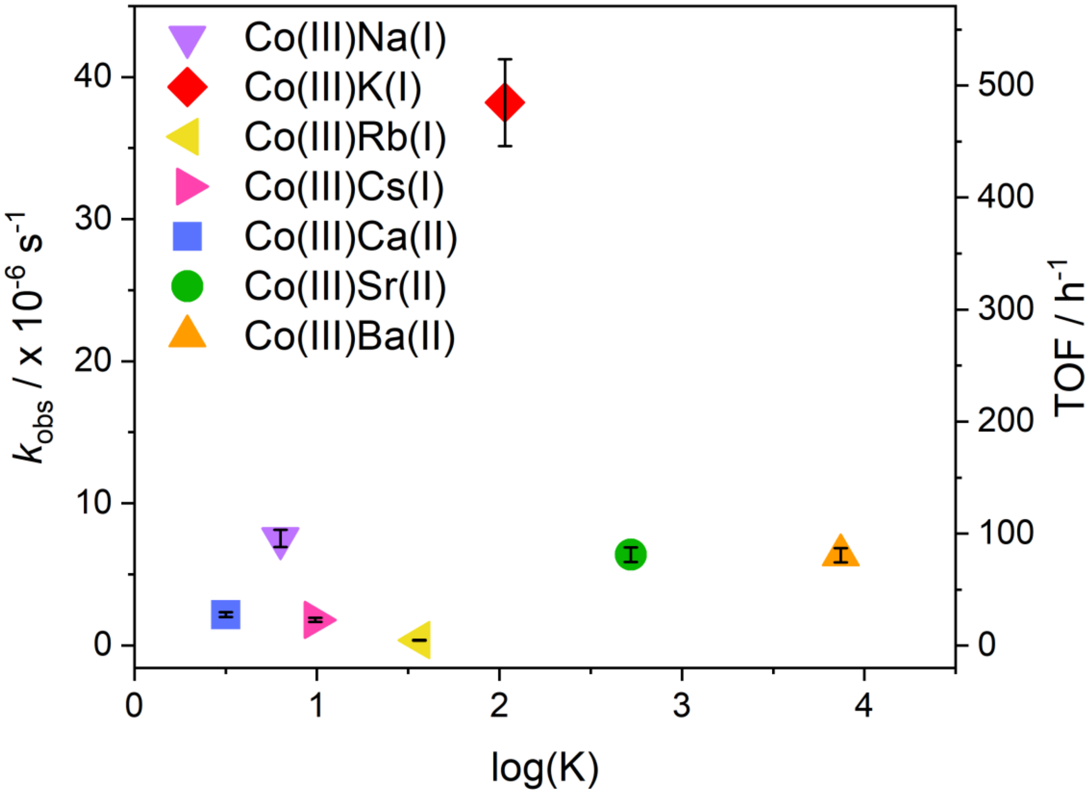

Supplementary Fig 34. Plot of k_{obs} and TOF against log(K) (where K is the equilibrium constant for M(I/II) coordination within 18-crown-6 for a 1:1 reaction in aqueous solution at 25 °C) for PO/CO_2 ROCOP catalysed by Co(III)M(I/II).[3]

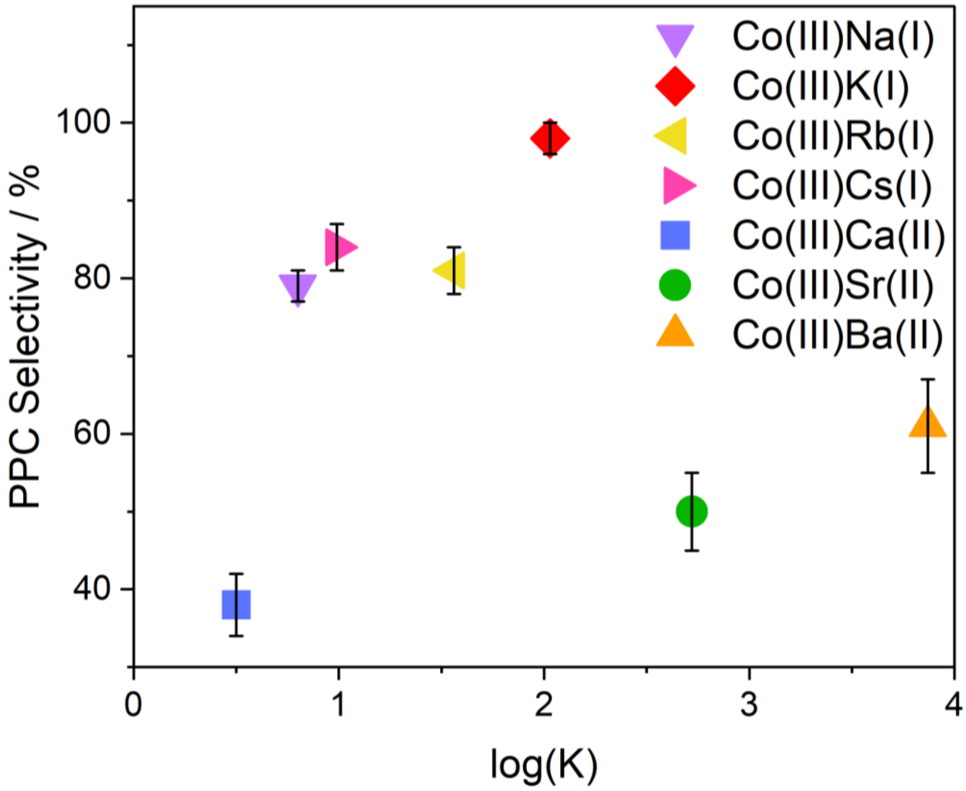

Supplementary Fig 35. Plot of selectivity for PPC against log(K) (where K is the equilibrium constant for M(I/II) coordination within 18-crown-6 for a 1:1 reaction in aqueous solution at 25 °C) for PO/CO_2 ROCOP catalysed by Co(III)M(I/II).[3]

Supplementary Tab 3: Values for derivation of supplementary equation 2.

Conversion / %	TON	$[PA]_t$ / M
5	5	1.36
70	70	0.43

Supplementary Tab 4: Description of parameters used for the derivation of supplementary equation 2.

Parameters	Description
$[PA]_t$	Concentration of PA at time *t*
$[PA]_0$	Starting concentration of PA (i.e. at time = 0 s)
k_{obs}	Observed rate coefficient
t	Time
t_i	Initiation time
$TOF_{5\text{-}70\%}$	Turnover frequency between 5 and 70% conversion
$TON_{70\%}$	Turnover number at 70% conversion
$TON_{5\%}$	Turnover number at 5% conversion
$t_{70\%}$	Time at 70% conversion
$t_{5\%}$	Time at 5% conversion

Derivation of Supplementary Equation 2:

$$[\mathrm{PA}]_\mathrm{t} = -k_\mathrm{obs}t + t_i$$

$$\mathrm{TOF}_{5-70\%} = \frac{\mathrm{TON}_{70\%} - \mathrm{TON}_{5\%}}{t_{70\%} - t_{5\%}} = \frac{70-5}{t_{70\%} - t_{5\%}} = \frac{65}{t_{70\%} - t_{5\%}}$$

$$t = -\frac{[\mathrm{PA}]_\mathrm{t} + c}{k_\mathrm{obs}}$$

$$t_{5\%} = \frac{1.36 - t_i}{-k_\mathrm{obs}} \quad \mathrm{t}_{70\%} = \frac{0.43 - t_i}{-k_\mathrm{obs}}$$

$$t_{70\%} - t_{5\%} = \frac{0.43 - t_i}{-k_\mathrm{obs}} - \frac{1.36 - t_i}{-k_\mathrm{obs}} = \frac{0.93}{k_\mathrm{obs}}$$

$$\mathrm{TOF}_{5-70\%} = \frac{65 \times k_\mathrm{obs} \times 3600\ \mathrm{h}^{-1}}{0.93} = \boldsymbol{k_\mathrm{obs} \times 251.61 \times 10^3\ \mathrm{h}^{-1}} \qquad \text{Eq. 2}$$

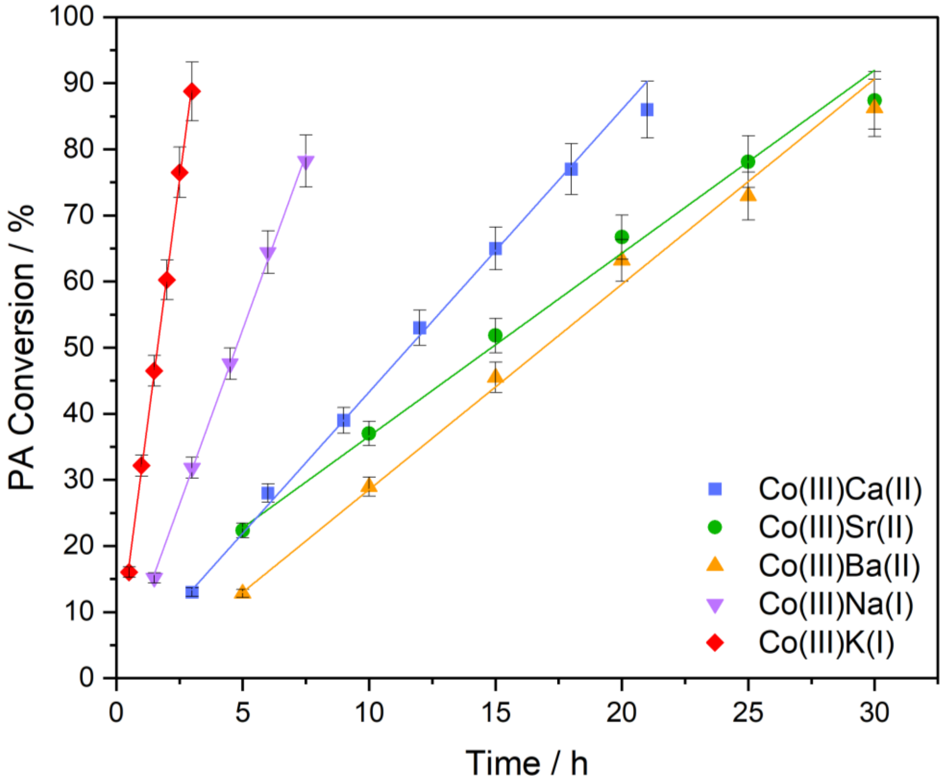

Supplementary Fig 36. Representative conversion vs. time plots for PO/PA ROCOP catalysed by Co(III)M(I/II) (M(I/II) = Na(I), K(I), Ca(II), Sr(II), Ba(II)).

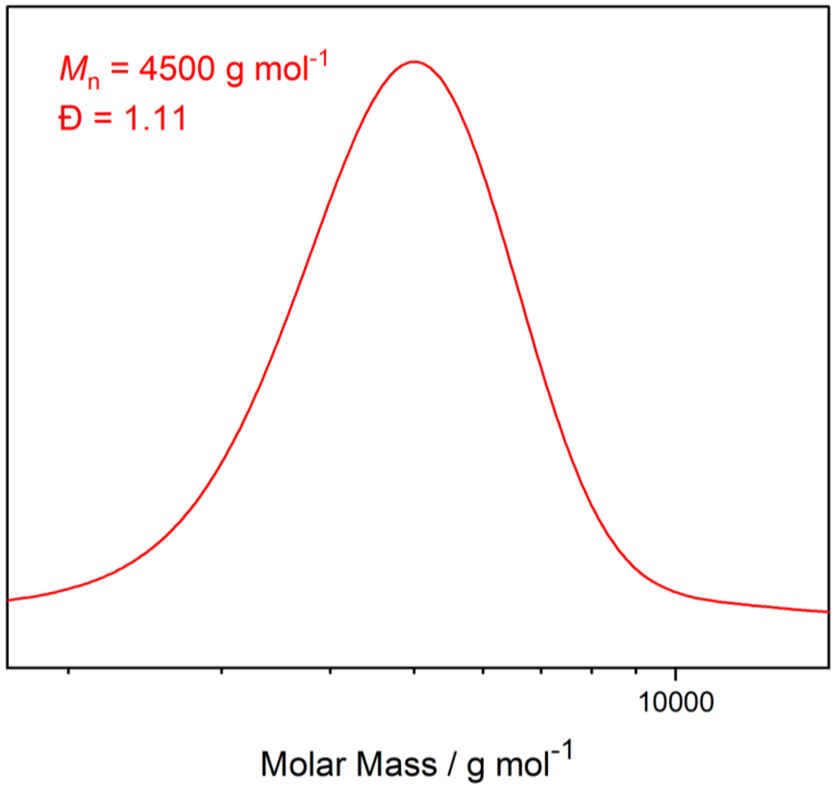

Supplementary Fig 37. GPC trace for PO/PA ROCOP catalysed by Co(III)K(I) in THF, using narrow dispersity polystyrene standards.

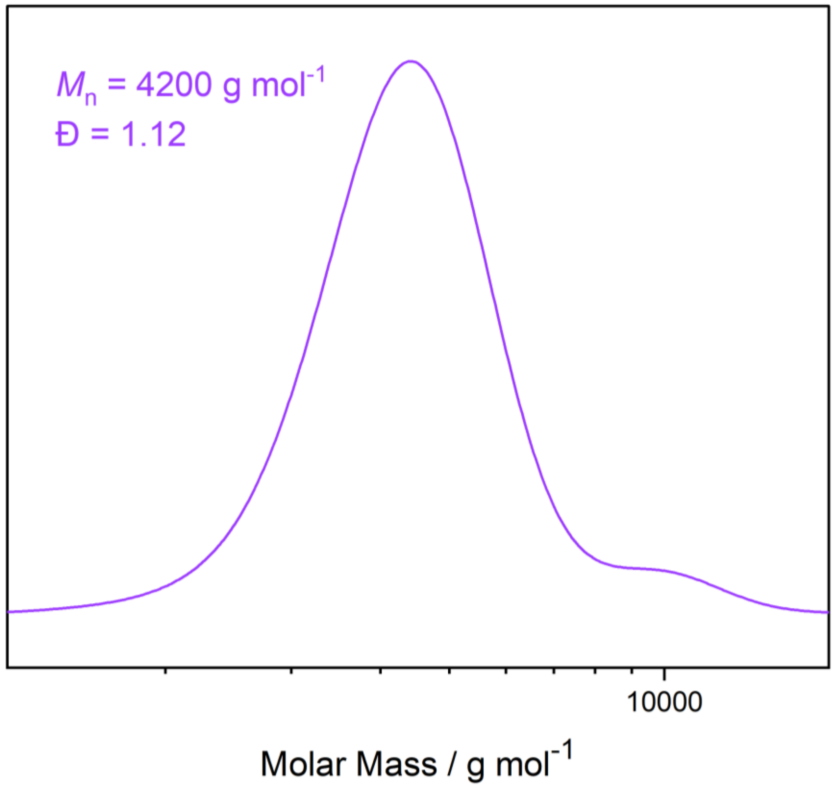

Supplementary Fig 38. GPC trace for PO/PA ROCOP catalysed by Co(III)Na(I) in THF, using narrow dispersity polystyrene standards.

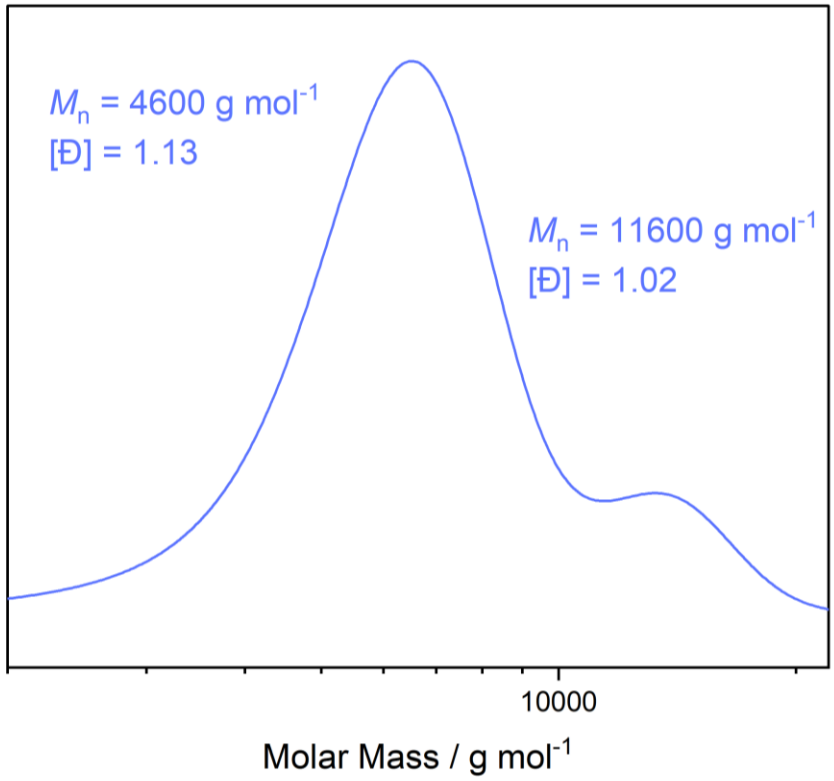

Supplementary Fig 39. GPC trace for PO/PA ROCOP catalysed by Co(III)Ca(II) in THF, using narrow dispersity polystyrene standards.

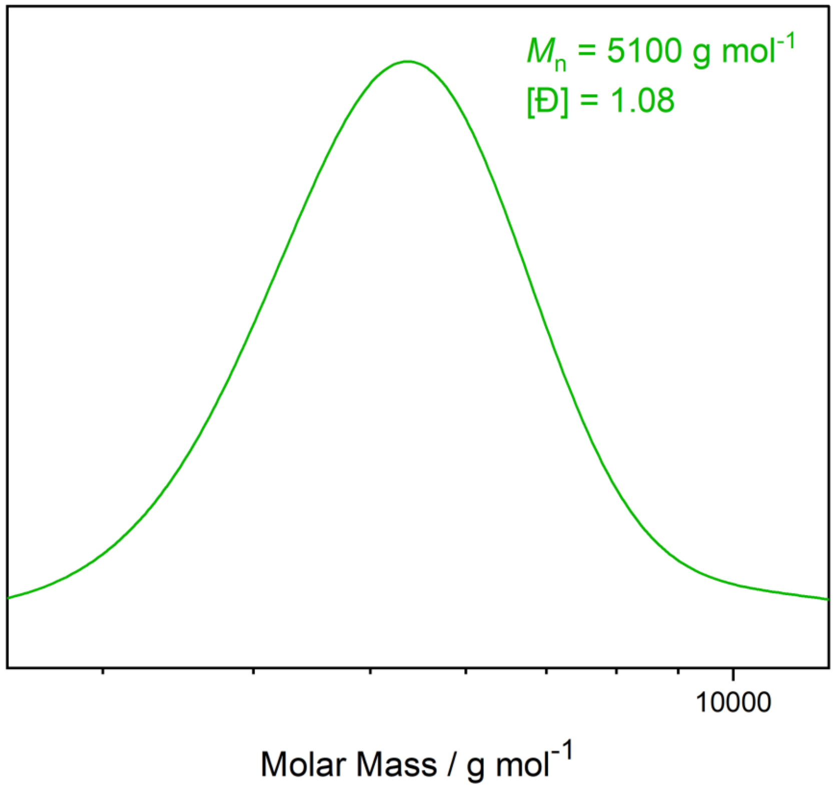

Supplementary Fig 40. GPC trace for PO/PA ROCOP catalysed by Co(III)Sr(II) in THF, using narrow dispersity polystyrene standards.

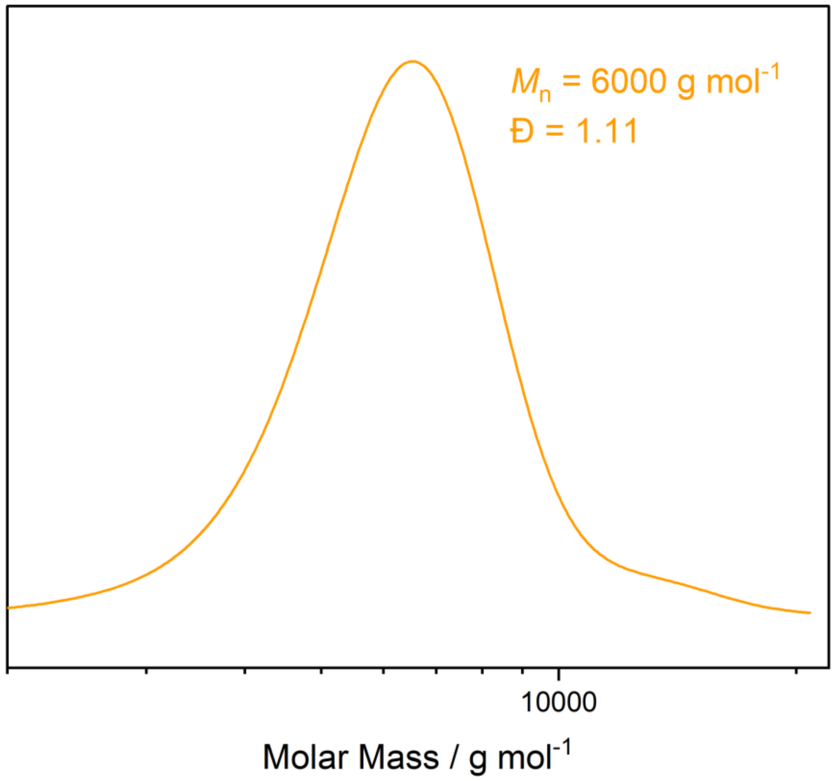

Supplementary Fig 41. GPC trace for PO/PA ROCOP catalysed by Co(III)Ba(II) in THF, using narrow dispersity polystyrene standards.

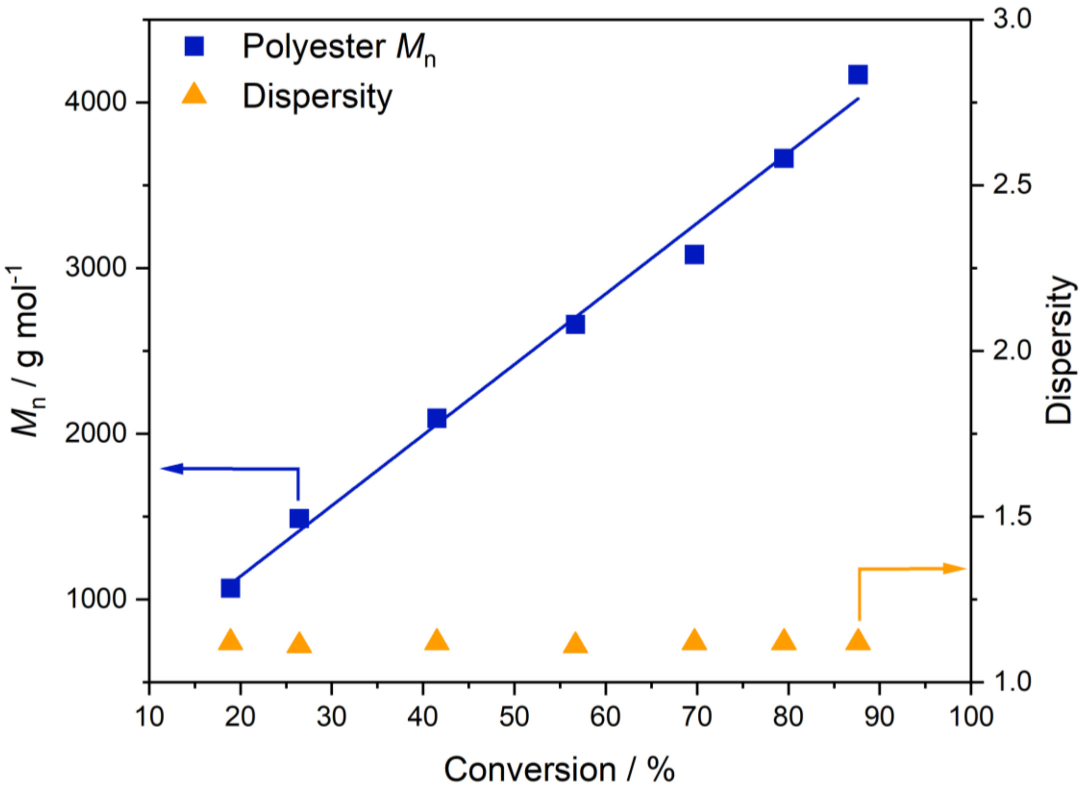

Supplementary Fig 42. Plots of molar mass (blue squares and line) and dispersity (orange triangles) of polyester against conversion for PO/PA ROCOP catalysed by Co(III)Na(I).

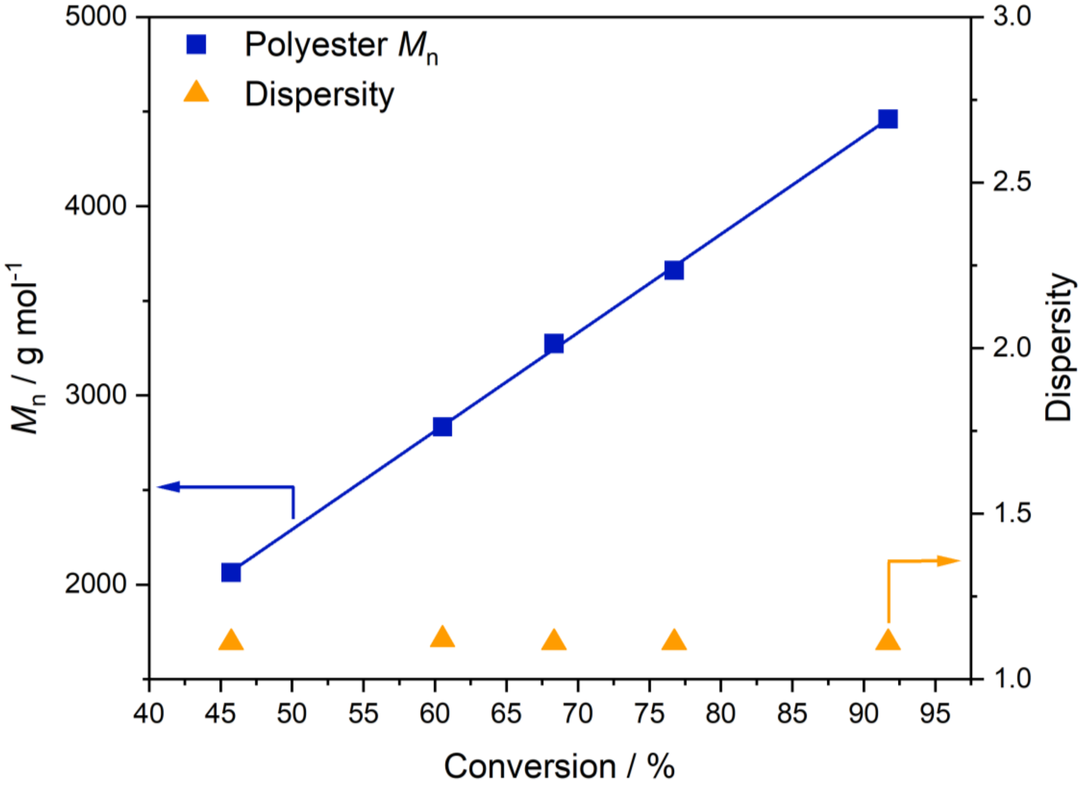

Supplementary Fig 43. Plots of molar mass (blue squares and line) and dispersity (orange triangles) of polyester against conversion for PO/PA ROCOP catalysed by Co(III)K(I).

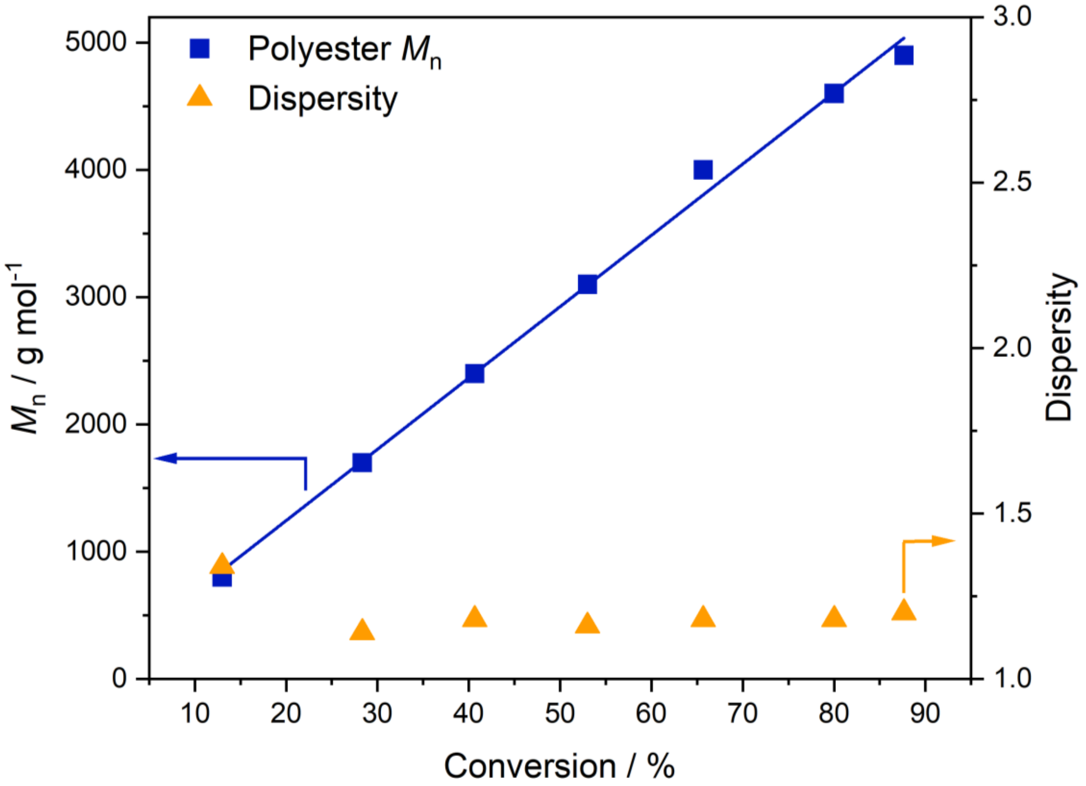

Supplementary Fig 44. Plots of molar mass (blue squares and line) and dispersity (orange triangles) of polyester against conversion for PO/PA ROCOP catalysed by Co(III)Ca(II).

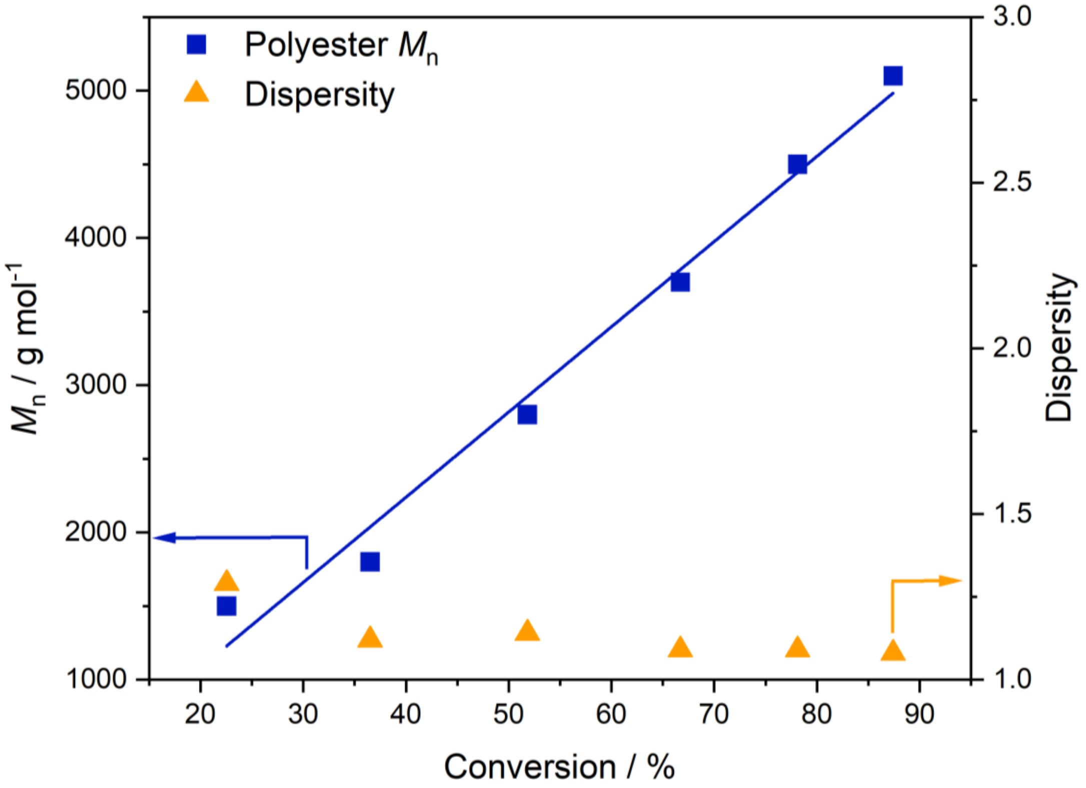

Supplementary Fig 45. Plots of molar mass (blue squares and line) and dispersity (orange triangles) of polyester against conversion for PO/PA ROCOP catalysed by Co(III)Sr(II).

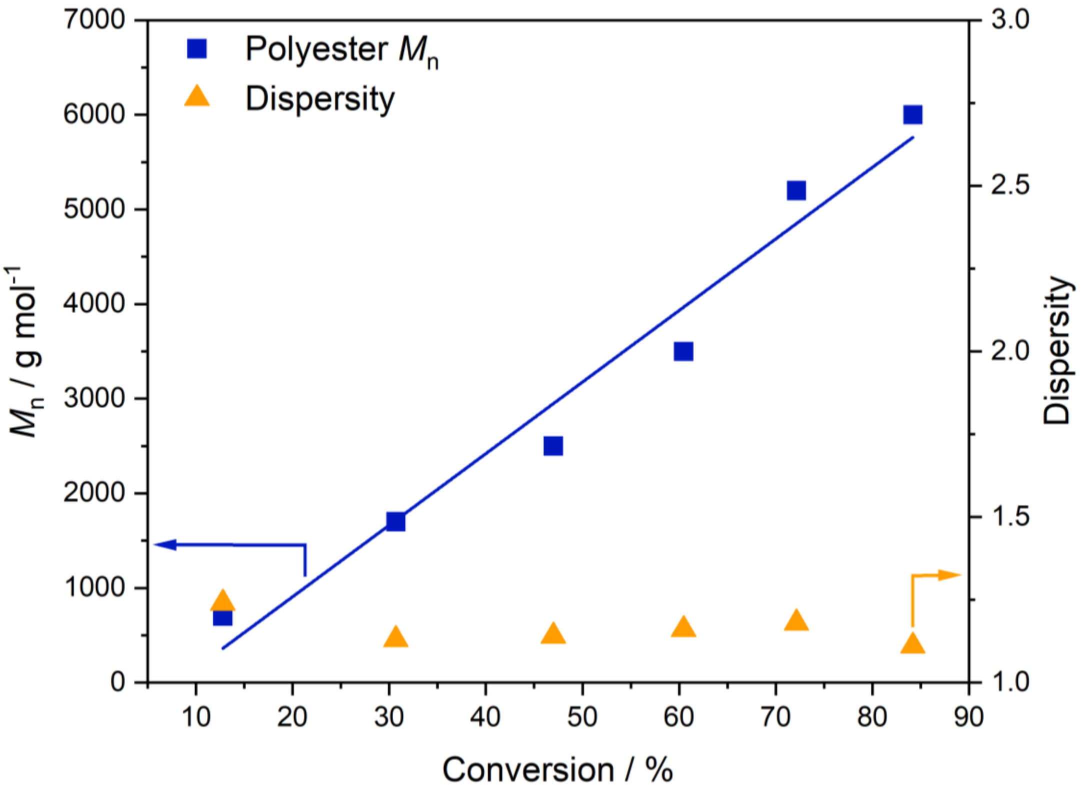

Supplementary Fig 46. Plots of molar mass (blue squares and line) and dispersity (orange triangles) of polyester against conversion for PO/PA ROCOP catalysed by Co(III)Ba(II).

Supplementary Tab 5. Epoxide/anhydride ROCOP data for a range of epoxide and anhydride combinations with Co(III)K(I).

Entry	Epoxide	Anhydride	Time / h	Conv. [b] / %	Polyester [c] / %	TOF [d] / h^{-1}	M_n [Đ] [e] / g mol^{-1}
1	PO	PA	1	93	> 99	93	9900 [1.12]
2	PO	THPA	1	96	> 99	96	1700 [1.23]
3	PO	TCA	4	96	> 99	24	14300 [1.07]
4	PO	NBA	1.5	76	> 99	51	7200 [1.13]
5	PO	CA	3	89	> 99	30	11700 [1.16]
6	BO	PA	0.5	37	> 99	74	4100 [1.33]
7	SO	PA	0.5	46	> 99	92	3300 [1.24]
8	CHO	PA	0.5	85	> 99	170	8800 [1.15]

[a] Reaction conditions: 1:100 $[catalyst]_0$:$[anhydride]_0$, where $[catalyst]_0$ = 14.3 mM) in 1 mL neat epoxide (for PO/PA ROCOP this corresponds to 1:100:1000 $[catalyst]_0$:$[anhydride]_0$:$[epoxide]_0$). [b] Conversion determined by ^{1}H NMR spectroscopy through comparison of resonances associated with anhydride and polyester. [c] Selectivity for polyester over polyether, determined by comparison of ^{1}H NMR spectroscopy corresponding to polyester and polyether resonances. [d] Turnover frequency for polyester formation, TOF = TON/time; turnover number (TON) for polyester formation; number of moles of anhydride consumed/number of moles of catalyst; since 1:100 $[catalyst]_0$:$[anhydride]_0$ was used, TON = conversion. [e] Determined by gel permeation chromatography (GPC) in THF at 30 °C, using narrow dispersity polystyrene standards.

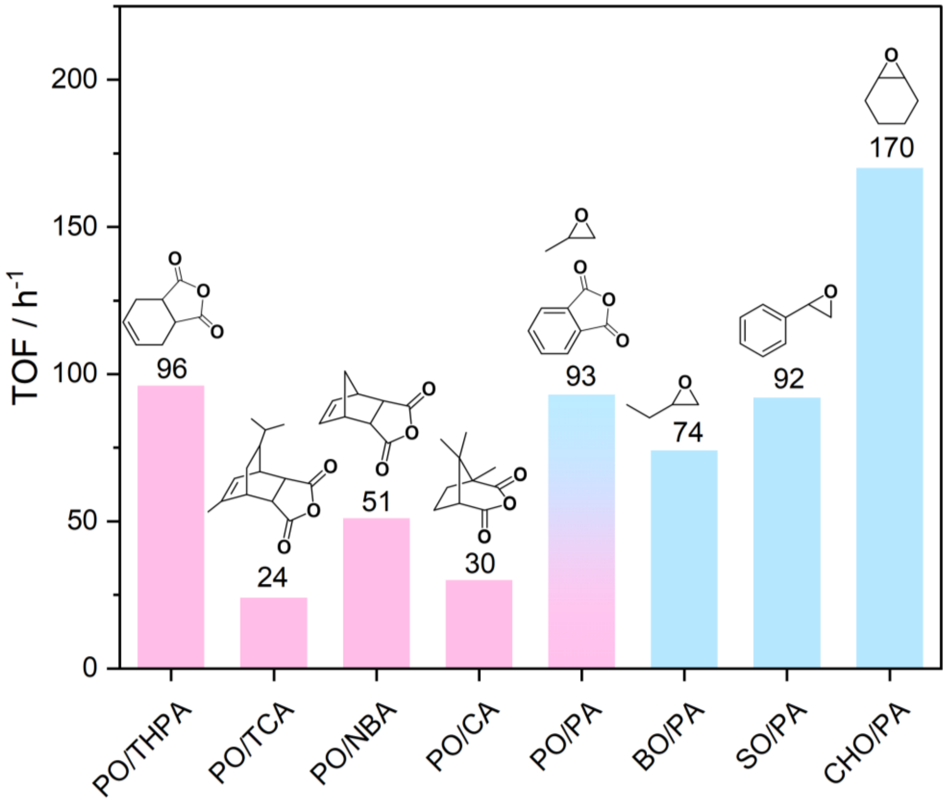

Supplementary Fig 47. TOFs for anhydride (pink; LHS) and epoxide (blue; RHS) scope using Co(III)K(I) (0.1 mol% catalyst, 10 mol% anhydride in neat epoxide, 60 °C). The activity for PO/PA (blue/pink gradient; middle) is included as a reference.

Epoxide/anhydride scope polymerisations were conducted at 60 °C, to maximise activities without deactivating the catalyst, using 1:100 $[catalyst]_0$:$[anhydride]_0$ ($[catalyst]_0$ = 14.3 mM) in 1 mL epoxide.

Increasing the temperature for PO/PA ROCOP from 50 °C to 60 °C results in an increase in activity from 50 h^{-1} to 93 h^{-1}. The activity for PO/tetrahydrophthalic anhydride (THPA) ROCOP is very similar (TOF = 96 h^{-1}), which would be expected due to the similarities in their structures. This result also indicates that the aromaticity of PA does not have any significant electronic effects for its copolymerisation with PO. The copolymerisation of PO with the bulky tricyclic anhydride (TCA) and camphoric anhydride (CA), have significantly reduced activities (TOF = 24 h^{-1} and 30 h^{-1} respectively), likely due to the methyl and isopropyl substituents on their backbones hindering anhydride ring-opening. The copolymerisation of PO with norbornene anhydride (NBA) has a similar activity to those of the other tricyclic anhydrides tested (TOF = 43 h^{-1}), but is slightly greater due to the absence of bulky substituents on its backbone.

The activity for the ROCOP of butylene oxide (BO) with PA is slightly reduced relative to that of PO/PA (TOF = 74 h^{-1}) due to the reduced intrinsic molarity of BO vs. PO (11.5 M vs. 14.3 M respectively). Although the rate

of styrene oxide (SO) ROCOP with PA is very similar to that of PO/PA (92 h^{-1}), it has a significantly lower intrinsic molarity (8.8 M). Therefore, there must be a rate-enhancement effect due to inductive electron-withdrawal by the phenyl substituent, leading to epoxide pre-activation. Cyclohexene oxide (CHO) has an increased ring strain relative to the acyclic epoxides mentioned previously. This strain increases the thermodynamic drive towards ring-opening of epoxide during its copolymerisation with PA, leading to an increase in rate (TOF = 170 h^{-1}). For every anhydride and epoxide tested, Co(III)K(I) maintained its perfect selectivity for polyester linkages.

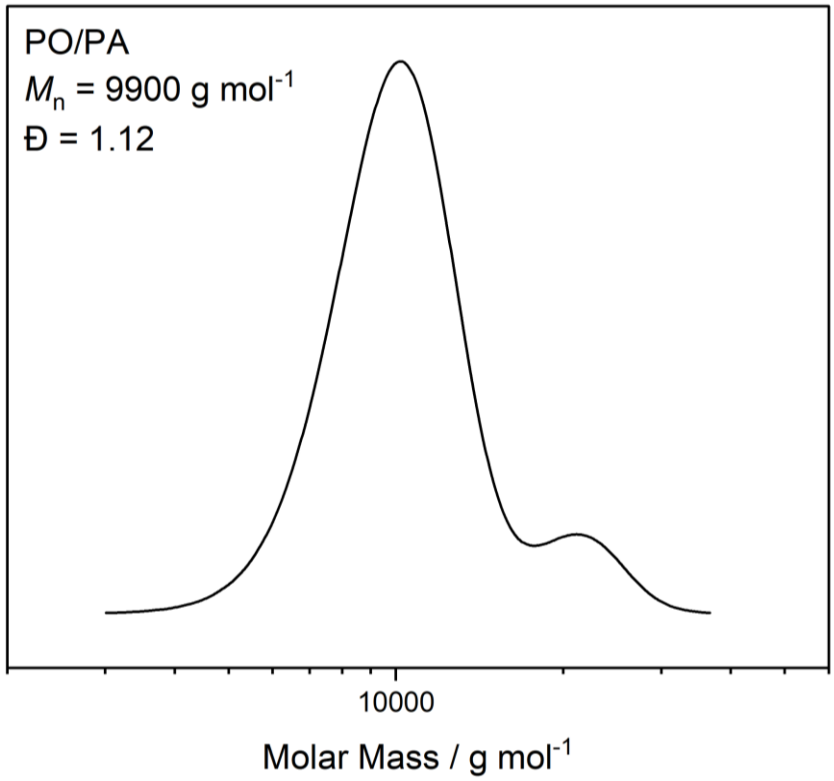

Supplementary Fig 48. GPC trace for PO/PA ring-opening copolymerisation with Co(III)K(I) in THF, using narrow dispersity polystyrene standards.

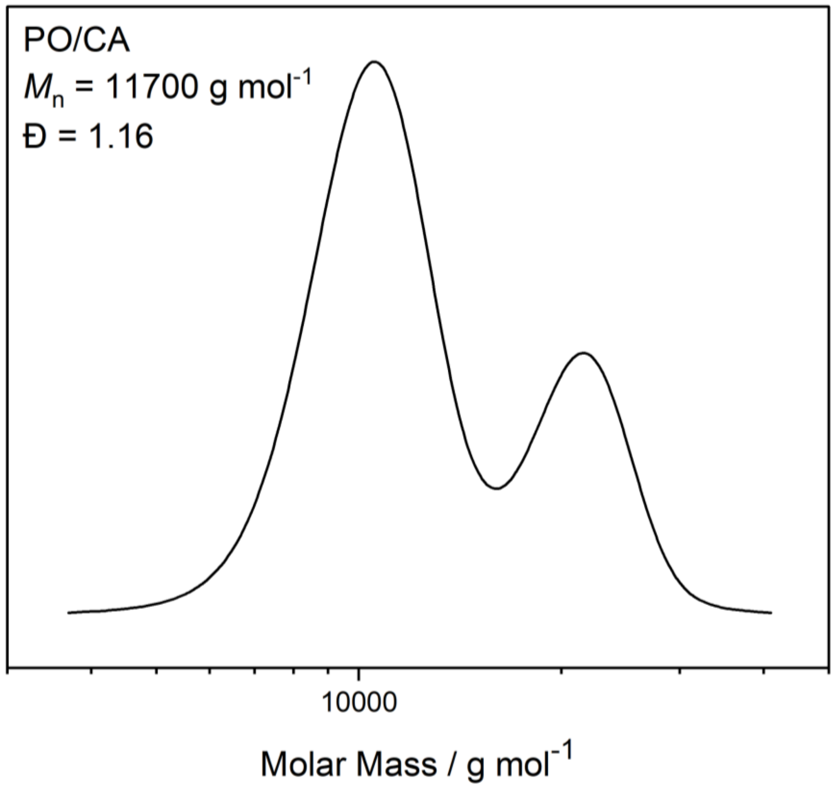

Supplementary Fig 49. GPC trace for PO/CA (camphoric anhydride) ring-opening copolymerisation with Co(III)K(I) in THF, using narrow dispersity polystyrene standards.

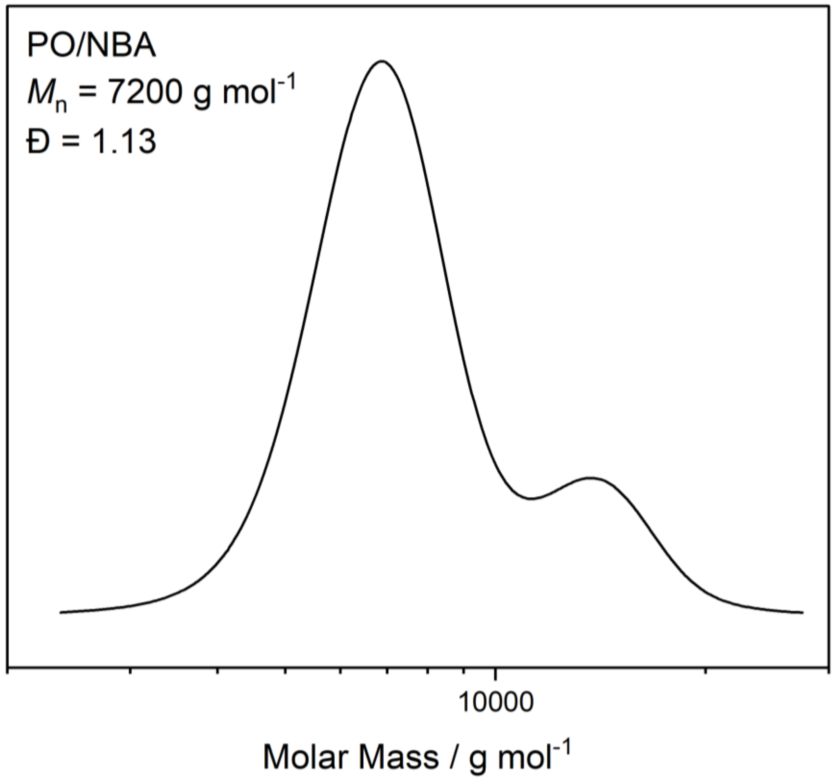

Supplementary Fig 50. GPC trace for PO/NBA (norbornene anhydride) ring-opening copolymerisation with Co(III)K(I) in THF, using narrow dispersity polystyrene standards.

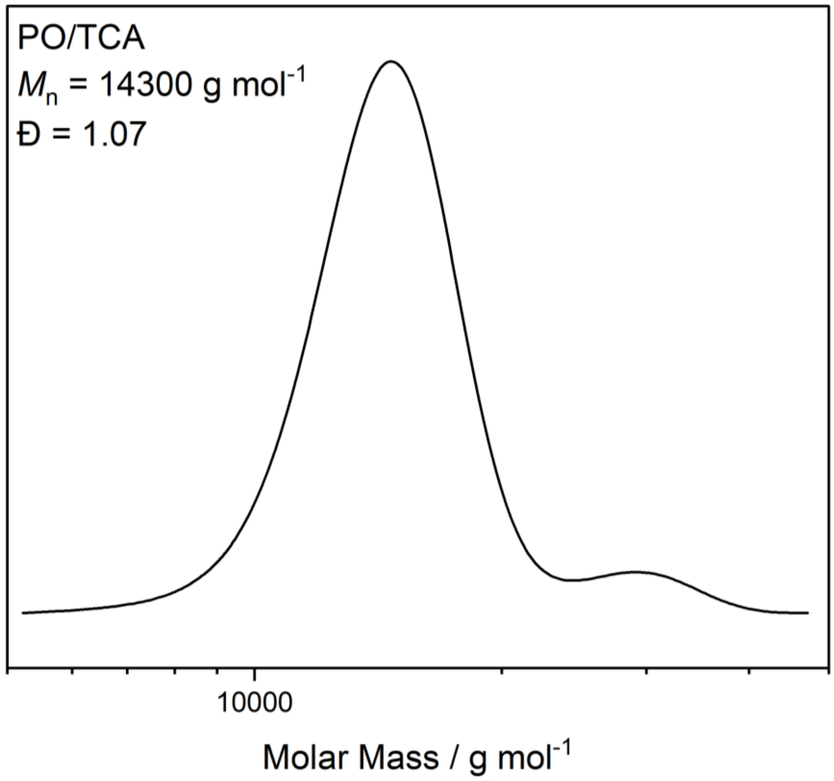

Supplementary Fig 51. GPC trace for PO/CA (tricyclic anhydride) ring-opening copolymerisation with Co(III)K(I) in THF, using narrow dispersity polystyrene standards.

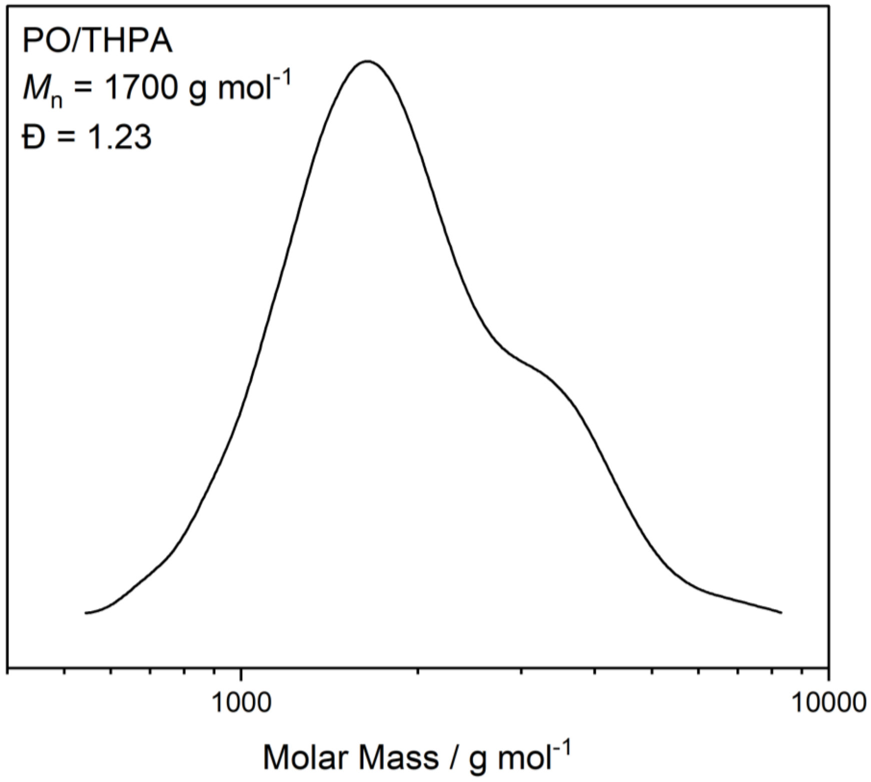

Supplementary Fig 52. GPC trace for PO/THPA (tetrahydrophthalic anhydride) ring-opening copolymerisation with Co(III)K(I) in THF, using narrow dispersity polystyrene standards.

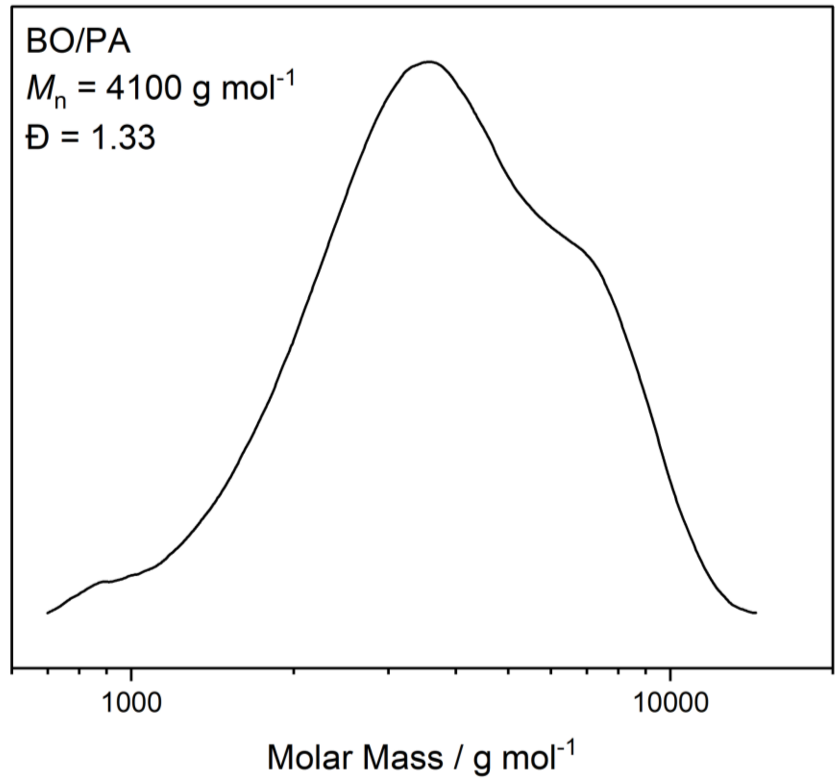

Supplementary Fig 53. GPC trace for BO/PA (butylene oxide) ring-opening copolymerisation with Co(III)K(I) in THF, using narrow dispersity polystyrene standards.

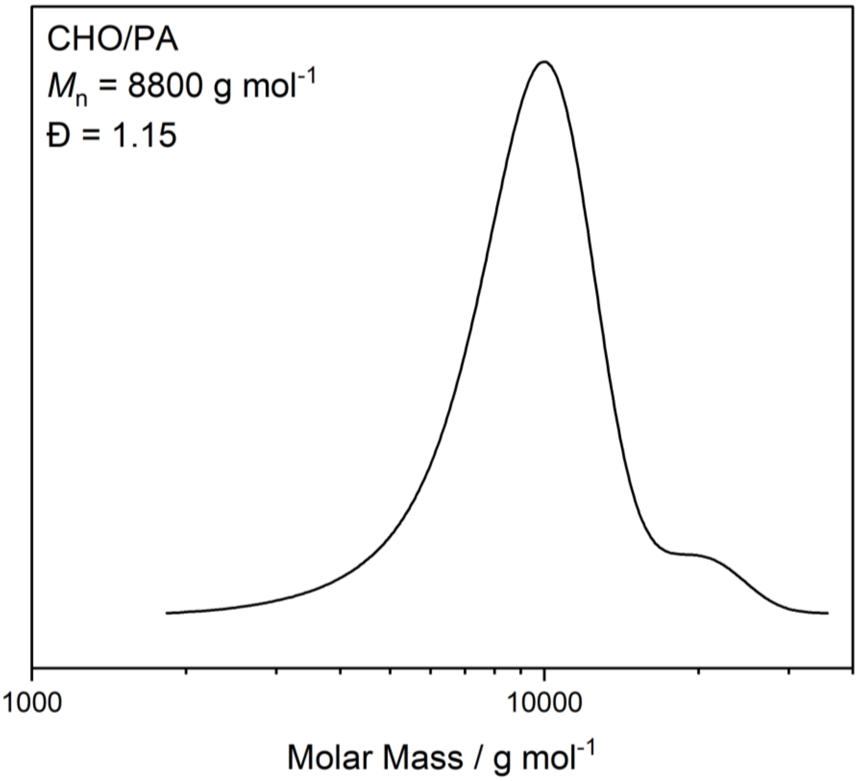

Supplementary Fig 54. GPC trace for CHO/PA (cyclohexene oxide) ring-opening copolymerisation with Co(III)K(I) in THF, using narrow dispersity polystyrene standards.

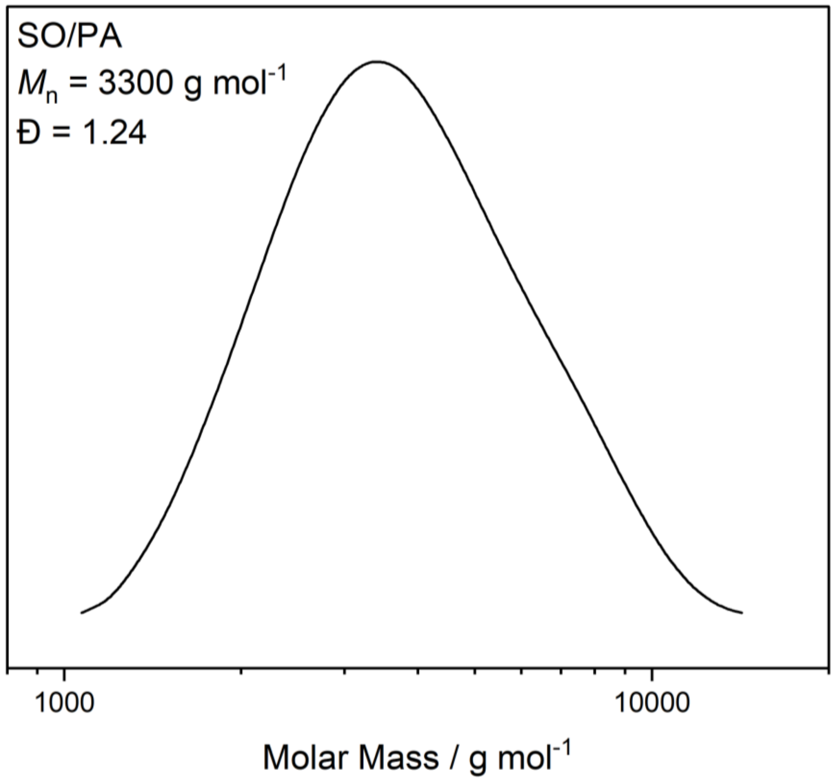

Supplementary Fig 55. GPC trace for SO/PA (styrene oxide) ring-opening copolymerisation with Co(III)K(I) in THF, using narrow dispersity polystyrene standards.

Supplementary Tab 6: Values for derivation of supplementary equation 3.

Conversion / %	TON	$[LA]_t$ / M	$\ln([LA]_t/[LA]_0)$
5	5	1.36	-0.05
70	70	0.43	-1.20

Supplementary Tab 7: Description of parameters used for the derivation of supplementary equation 3.

Parameters	Description
$[LA]_t$	Concentration of LA at time t
$[LA]_0$	Starting concentration of LA (i.e. at time = 0 s)
k_{obs}	Observed rate coefficient
t	Time
t_i	Initiation time
$TOF_{5\text{-}70\%}$	Turnover frequency between 5 and 70% conversion
$TON_{20\%}$	Turnover number at 20% conversion
$TON_{5\%}$	Turnover number at 5% conversion
$t_{20\%}$	Time at 20% conversion
$t_{5\%}$	Time at 5% conversion

Derivation of Supplementary Equation 3:

$$\ln\frac{[\mathrm{LA}]_\mathrm{t}}{[\mathrm{LA}]_0} = -k_{\mathrm{obs}}t + t_\mathrm{i}$$

$$\mathrm{TOF}_{5-70\%} = \frac{\mathrm{TON}_{70\%} - \mathrm{TON}_{5\%}}{t_{70\%} - t_{5\%}} = \frac{70 - 5}{t_{70\%} - t_{5\%}} = \frac{65}{t_{70\%} - t_{5\%}}$$

$$t = \frac{\ln\frac{[\mathrm{LA}]_\mathrm{t}}{[\mathrm{LA}]_0} - t_\mathrm{i}}{-k_{\mathrm{obs}}}$$

$$t_{5\%} = \frac{0.05 - t_\mathrm{i}}{-k_{\mathrm{obs}}} \qquad t_{70\%} = \frac{1.20 - t_\mathrm{i}}{-k_{\mathrm{obs}}}$$

$$t_{70\%} - t_{5\%} = \frac{1.20 - t_\mathrm{i}}{-k_{\mathrm{obs}}} - \frac{0.05 - t_\mathrm{i}}{-k_{\mathrm{obs}}} = \frac{1.15}{k_{\mathrm{obs}}}$$

$$\mathrm{TOF}_{5-70\%} = \frac{65 \times k_{\mathrm{obs}} \times 3600\ \mathrm{h}^{-1}}{1.15} = \boldsymbol{k_{\mathrm{obs}} \times 203.48 \times 10^{3}\ \mathrm{h}^{-1}} \qquad \text{Eq. 3}$$

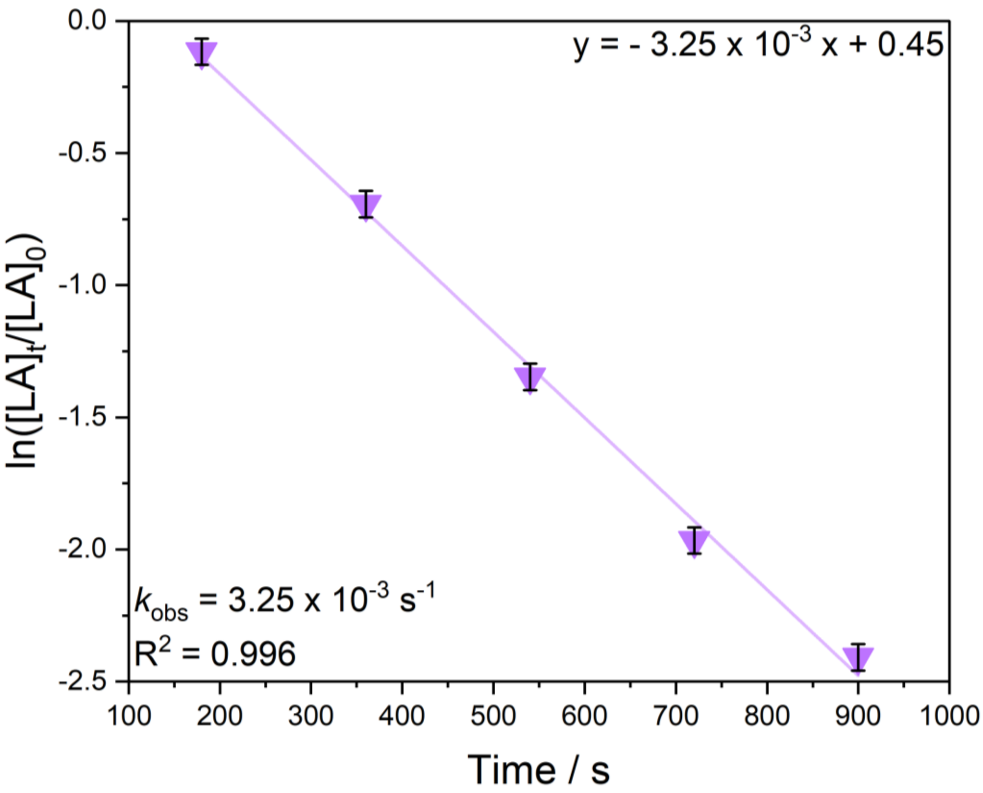

Supplementary Fig 56. Representative semi-logarithmic plot for *rac*-LA ROP using Co(III)Na(I), k_{obs} calculated from the gradient of the fit.

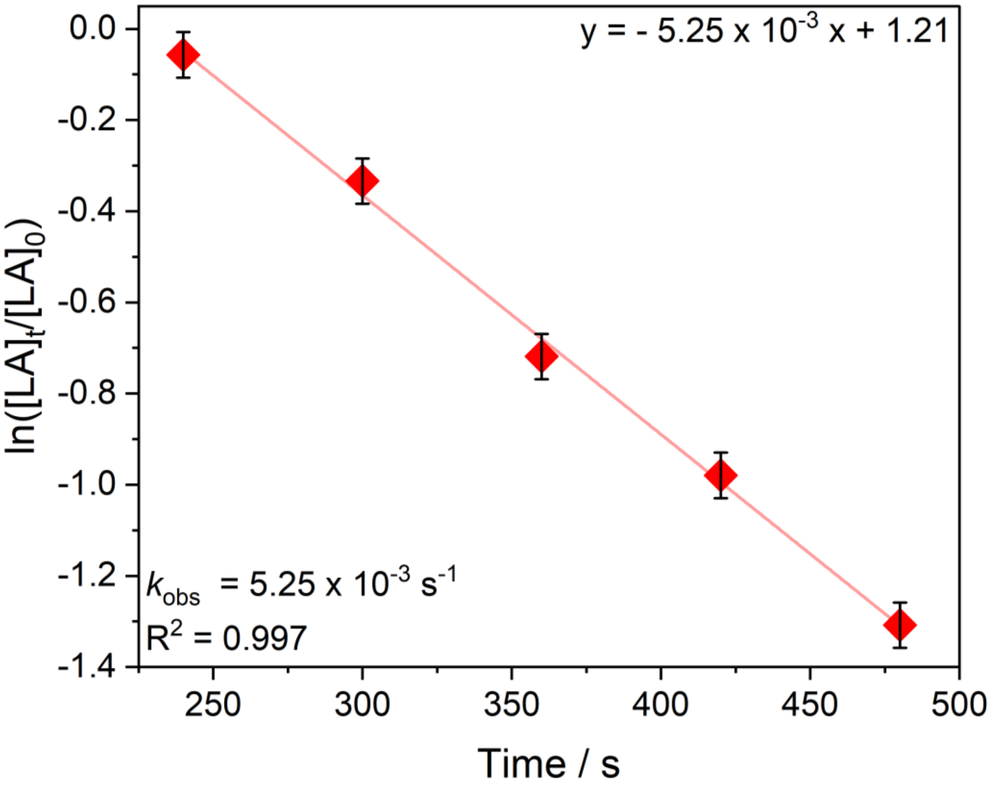

Supplementary Fig 57. Representative semi-logarithmic plot for *rac*-LA ROP using Co(III)Na(I), k_{obs} calculated from the gradient of the fit.

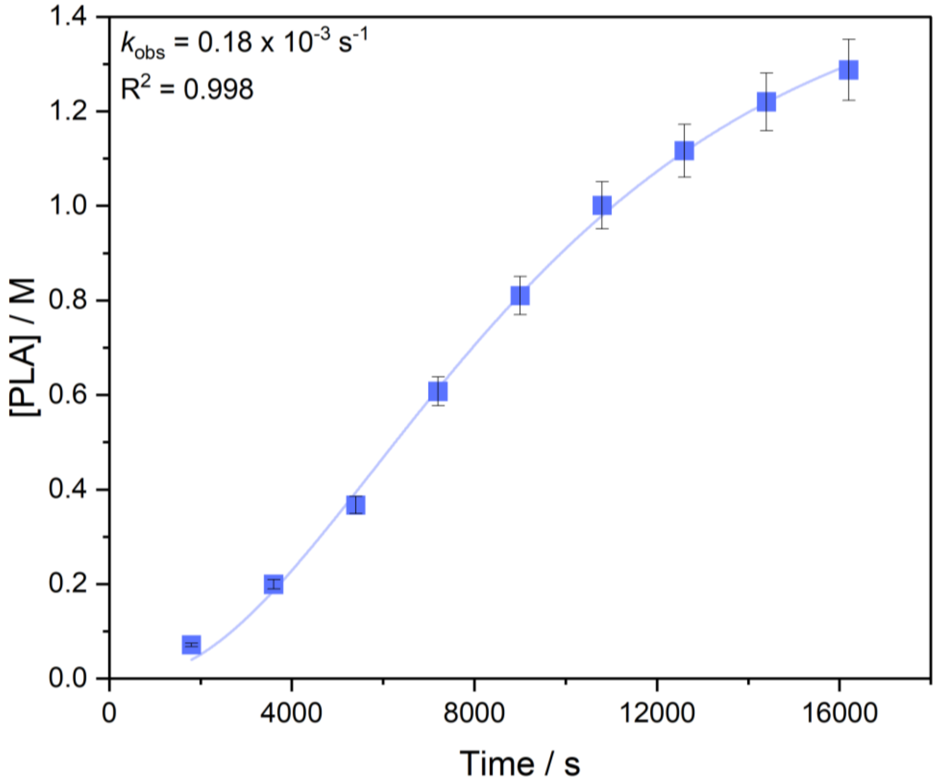

Supplementary Fig 58. Representative plot of [PLA] against time for *rac*-LA ROP using Co(III)Ca(II), conversion determined by ^{1}H NMR spectroscopy. k_{obs} is determined by fitting a sigmoidal function of the form [PLA] = A(1-exp(-k_{obs}t))^s, where s is the “sigmoidal factor”. [4]

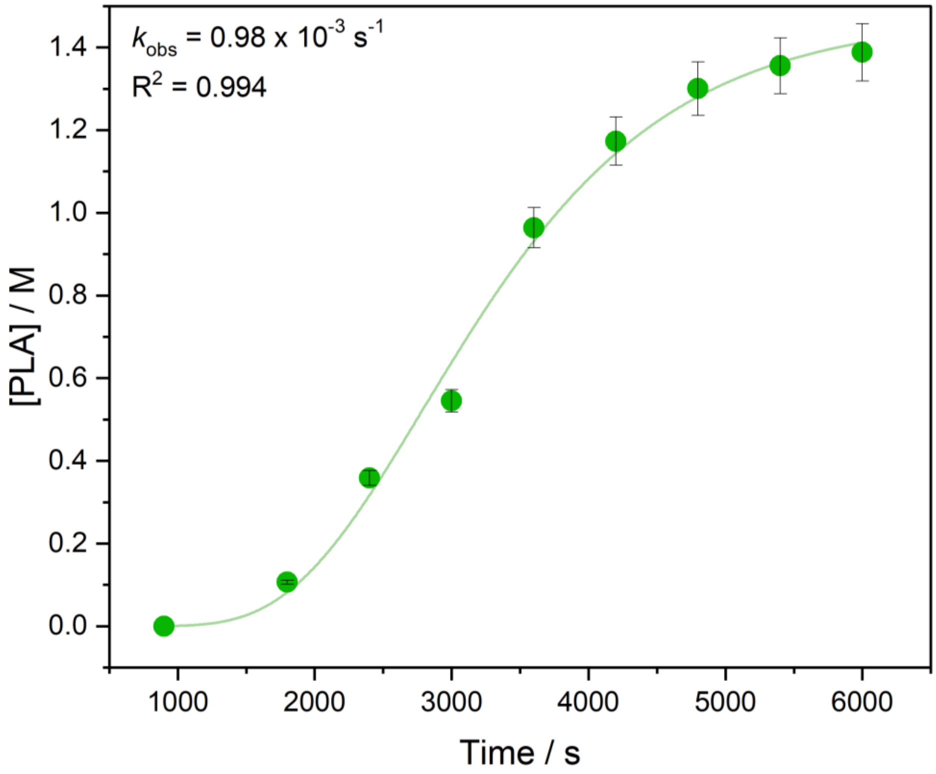

Supplementary Fig 59. Representative plot of [PLA] against time for *rac*-LA ROP using Co(III)Sr(II), conversion determined by ^{1}H NMR spectroscopy. k_{obs} is determined by fitting a sigmoidal function of the form [PLA] = A(1-exp(-k_{obs}t))^s, where s is the “sigmoidal factor”. [4]

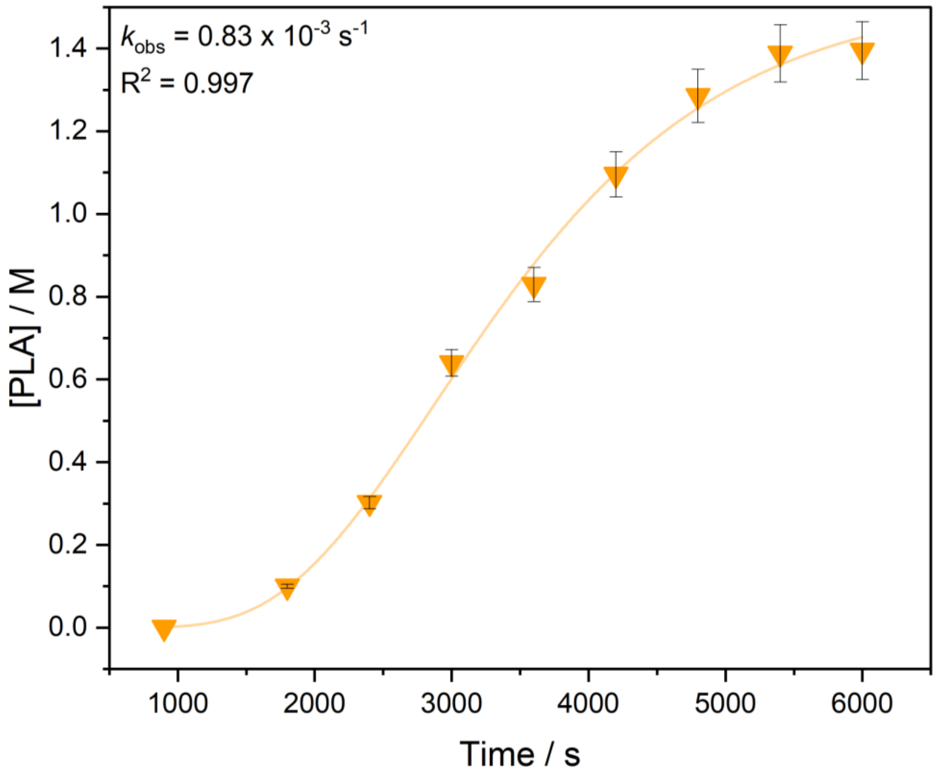

Supplementary Fig 60. Representative plot of [PLA] against time for *rac*-LA ROP using Co(III)Ba(II), conversion determined by ^{1}H NMR spectroscopy. k_{obs} is determined by fitting a sigmoidal function of the form [PLA] = A(1-exp(-k_{obs}t))^s, where s is the "sigmoidal factor". [4]

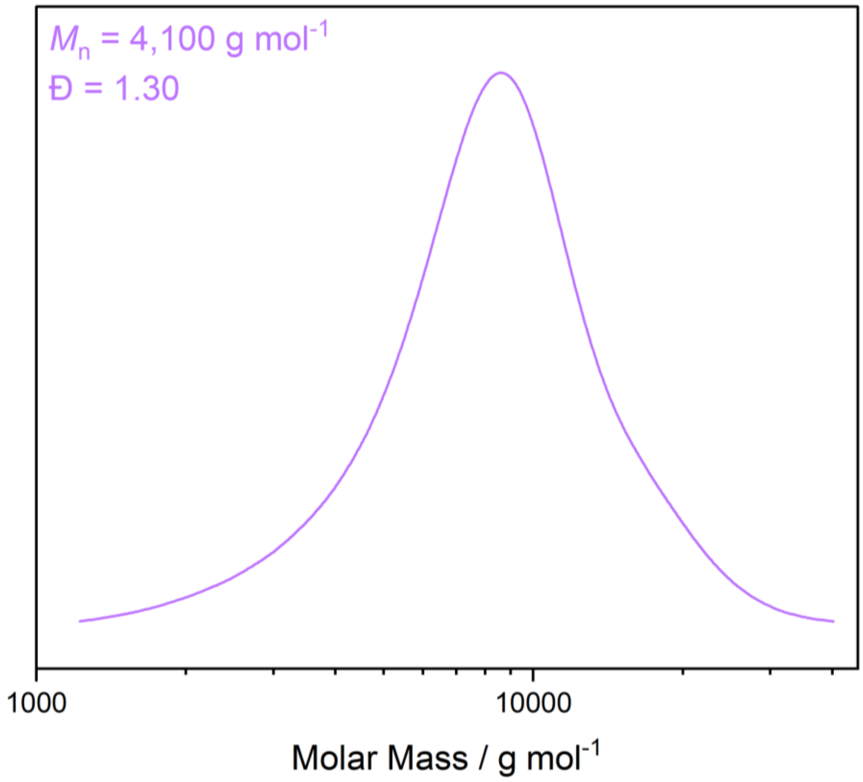

Supplementary Fig 61. GPC trace for *rac*-LA ROP catalysed by Co(III)Na(I) in THF, using narrow dispersity polystyrene standards.

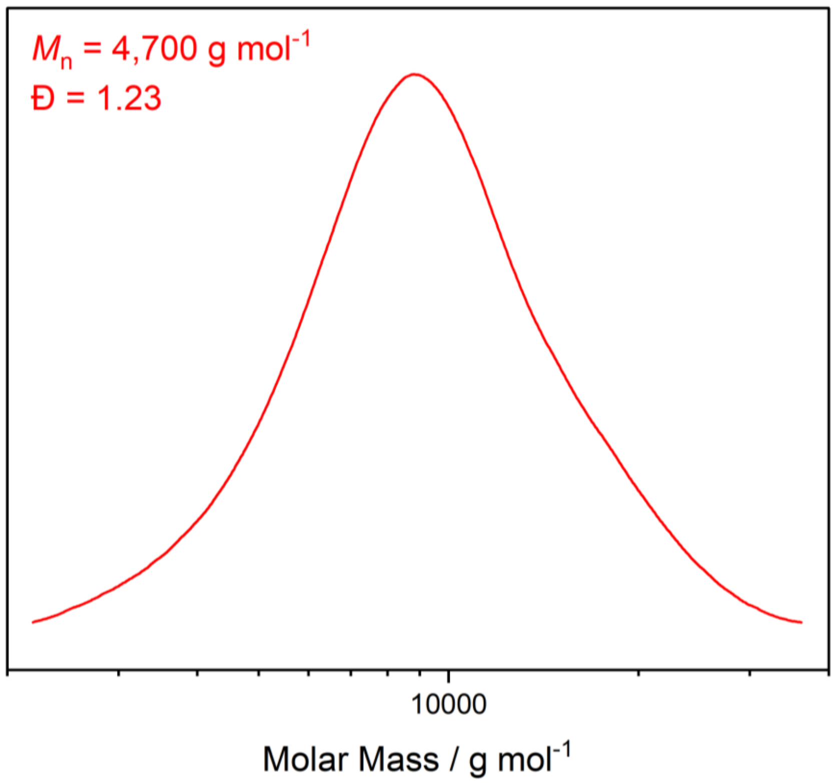

Supplementary Fig 62. GPC trace for *rac*-LA ROP catalysed by Co(III)K(I) in THF, using narrow dispersity polystyrene standards.

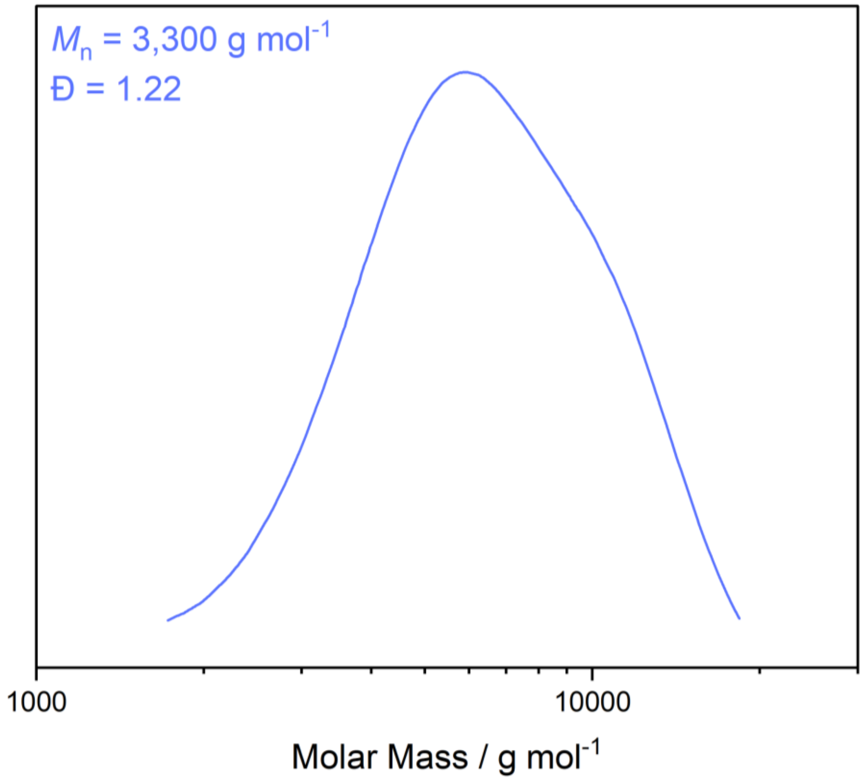

Supplementary Fig 63. GPC trace for *rac*-LA ROP catalysed by Co(III)Ca(II) in THF, using narrow dispersity polystyrene standards.

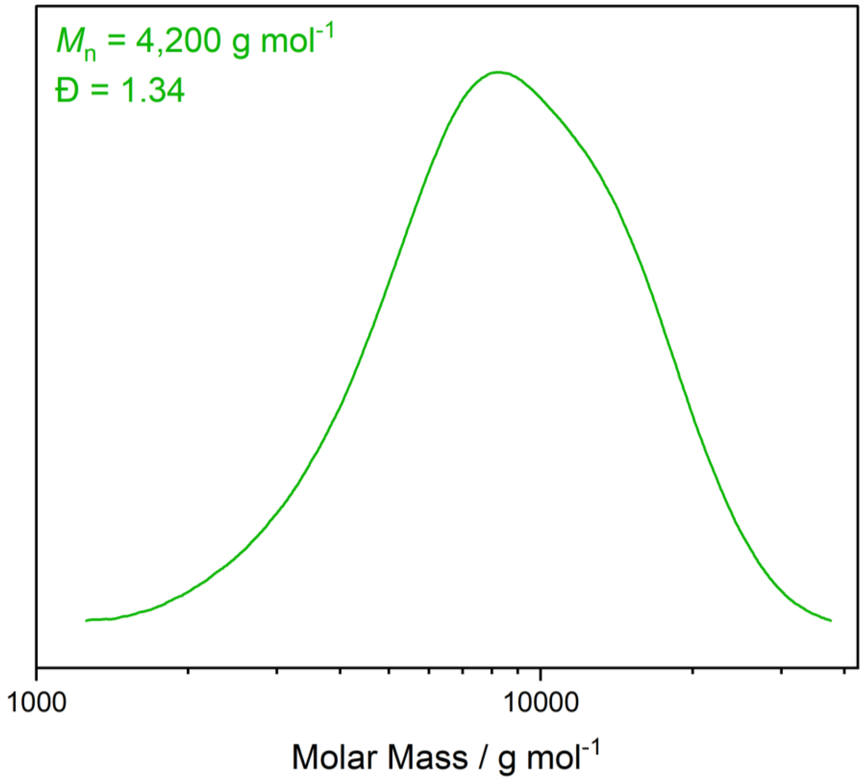

Supplementary Fig 64. GPC trace for *rac*-LA ROP catalysed by Co(III)Sr(II) in THF, using narrow dispersity polystyrene standards.

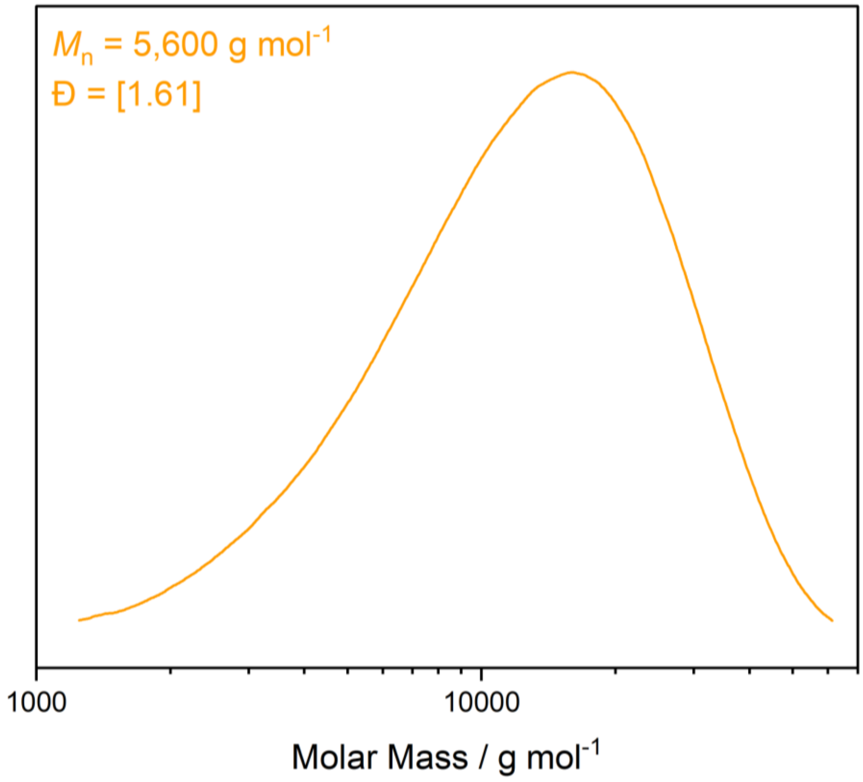

Supplementary Fig 65: GPC trace for *rac*-LA ROP catalysed by Co(III)Ba(II) in THF, using narrow dispersity polystyrene standards.

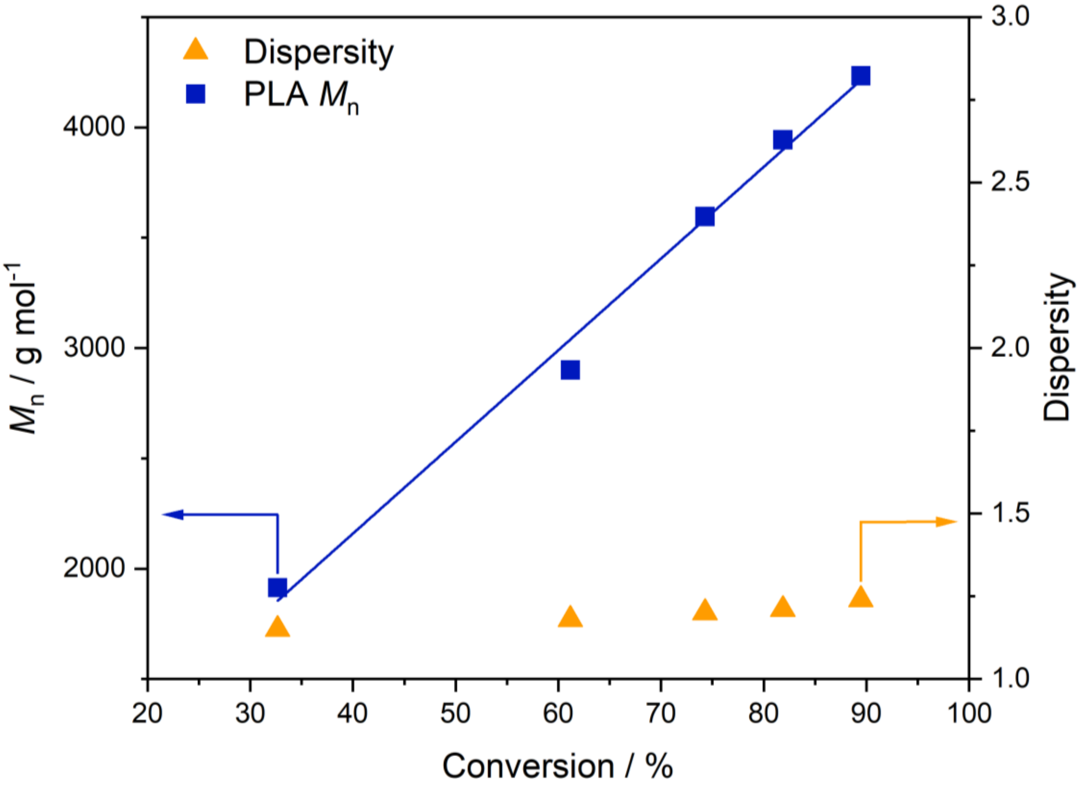

Supplementary Fig 66. Plots of molar mass (blue squares and line) and dispersity (orange triangles) of PLA against conversion for *rac*-LA ROP catalysed by Co(III)Na(I).

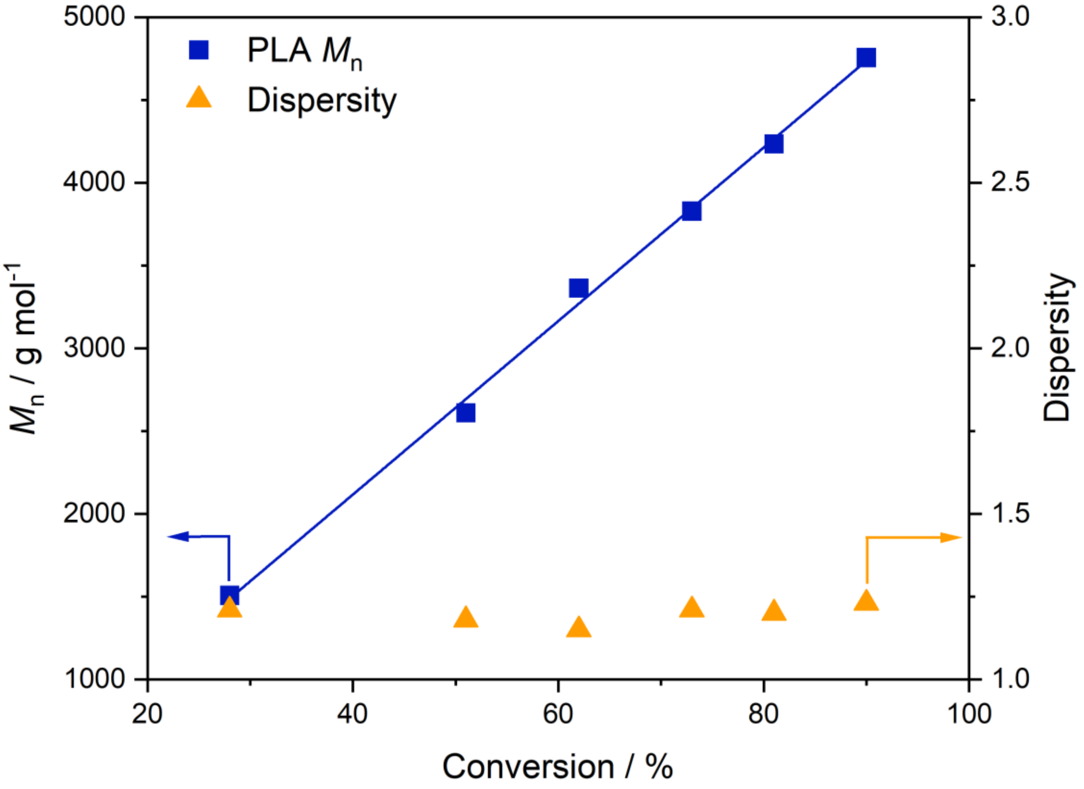

Supplementary Fig 67. Plots of molar mass (blue squares and line) and dispersity (orange triangles) of PLA against conversion for *rac*-LA ROP catalysed by Co(III)K(I).

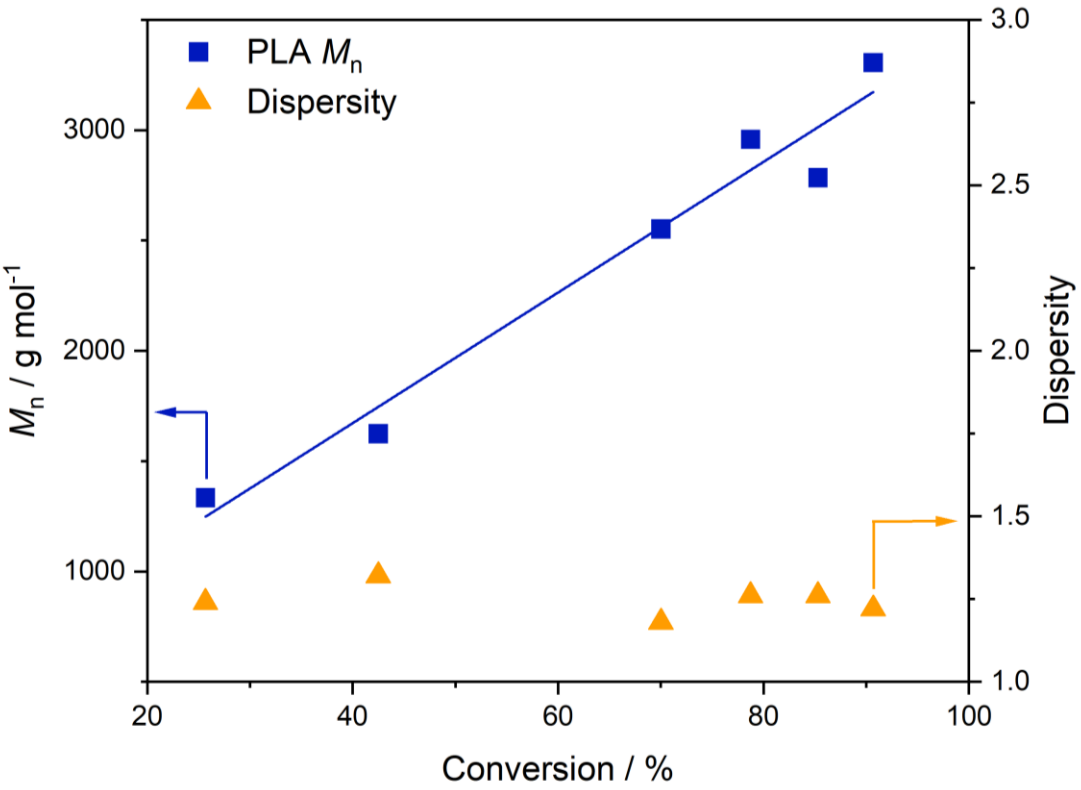

Supplementary Fig. Plots of molar mass (blue squares and line) and dispersity (orange triangles) of PLA against conversion for *rac*-LA ROP catalysed by Co(III)Ca(II).

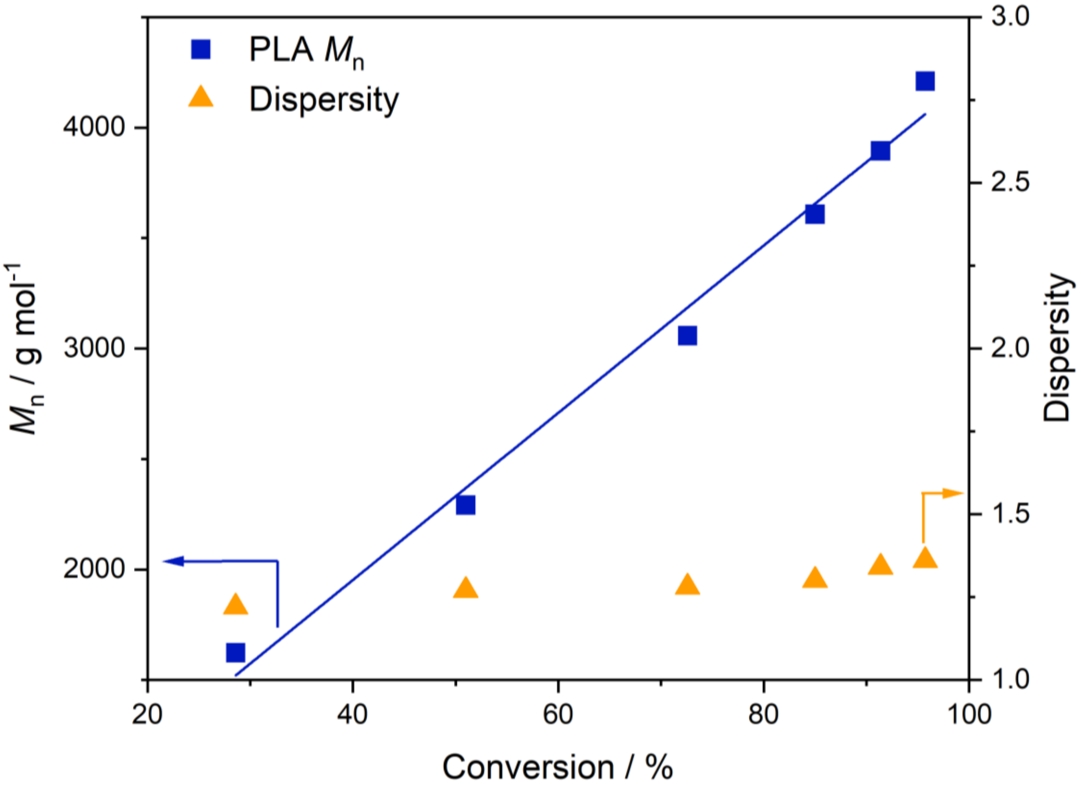

Supplementary Fig 68. Plots of molar mass (blue squares and line) and dispersity (orange triangles) of PLA against conversion for *rac*-LA ROP catalysed by Co(III)Sr(II).

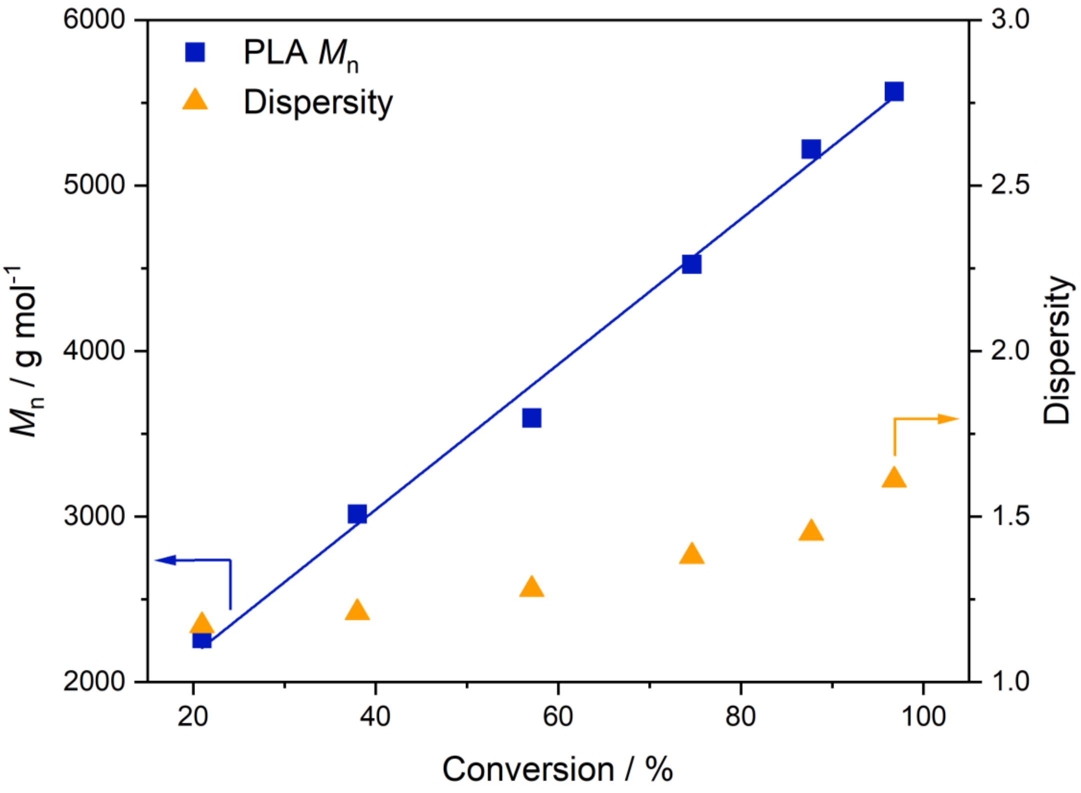

Supplementary Fig 69. Plots of molar mass (blue squares and line) and dispersity (orange triangles) of PLA against conversion for *rac*-LA ROP catalysed by Co(III)Ba(II).

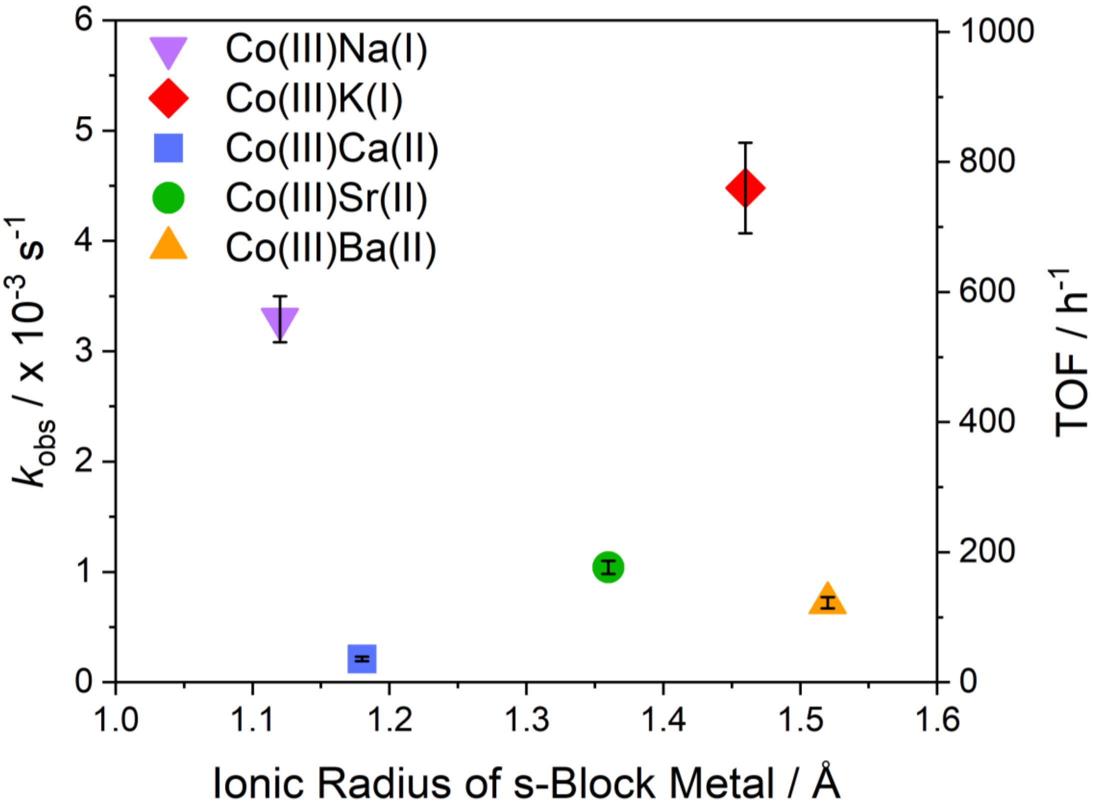

Supplementary Fig 70. Plot of k_{obs} against ionic radius of the s-block metal for *rac*-LA ROP catalysed by Co(III)M(I/II).[5]

Supplementary Tab 8: P_i values for Co(III)M(I/II) catalysts for *rac*-LA ROP.

Entry	Catalyst	P_i [b]
1	Co(III)Ca(II)	0.52
2	Co(III)Sr(II)	0.59
3	Co(III)Ba(II)	0.60
4	Co(III)Na(I)	0.63
5	Co(III)K(I)	0.72

[a] Reaction conditions: catalyst (0.1 mol%, 0.0143 mM), *rac*-LA (10 mol%, 1.43 mM), PO (1 mL, 14.3 M), 50 °C. [b] Determined by $^{1}H\{^{1}H\}$ NMR spectroscopy (supplementary fig 72, supplementary tab 9).

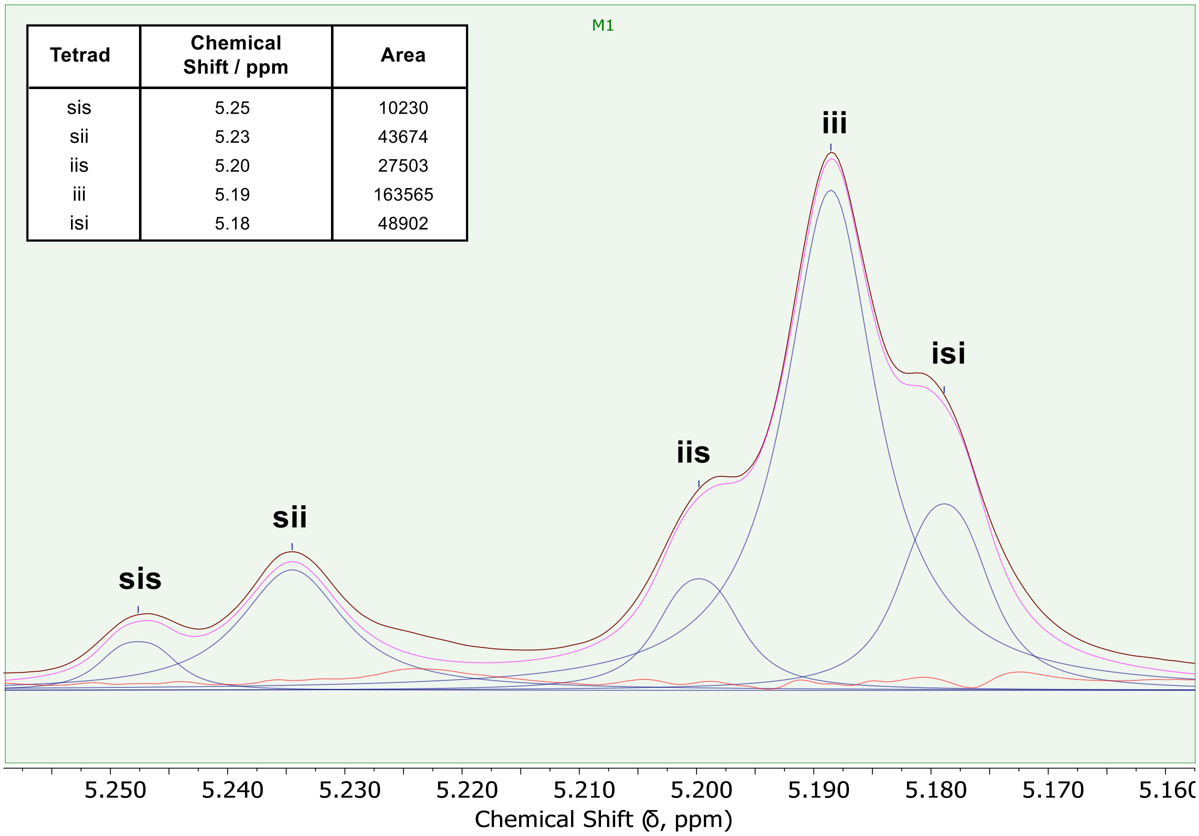

Supplementary Fig 71. Representative $^{1}H\{^{1}H\}$ NMR spectrum of PLA produced from *rac*-LA ROP by Co(III)K(I) for the determination of P_i (= 0.72).

Supplementary Tab 9. Bernoullian probabilities of the formation of each PLA tetrad for the calculation of P_i (the probability of isotactic enchainment of *rac*-LA).[6]

Tetrad	Probability
sis	$\frac{P_s^2}{2}$
sii	$\frac{P_s P_i}{2}$
iis	$\frac{P_s P_i}{2}$
iii	$P_i^2 + \frac{P_s P_i}{2}$
isi	$\frac{P_s^2 + P_s P_i}{2}$
$P_s + P_i = 1$	

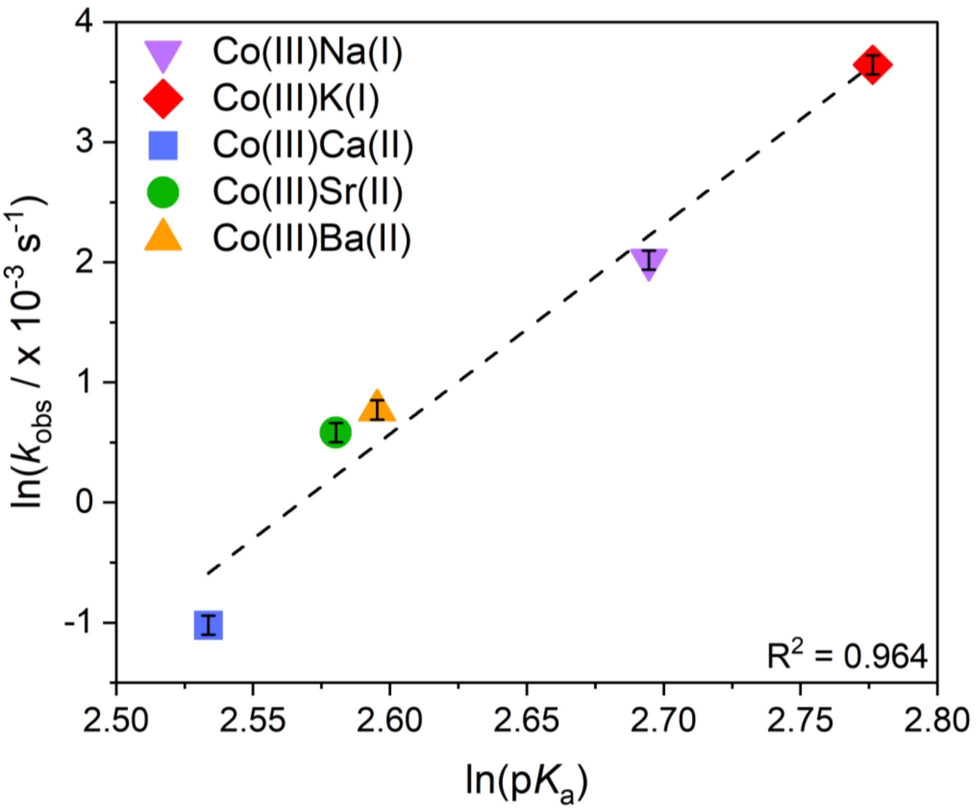

Supplementary Fig 72. Plot of ln(k_{obs}) vs. ln(pK_a) for PO/CO_2 ROCOP catalysed by Co(III)M(I/II) (M(I/II) = Na(I), K(I), Ca(II), Sr(II), Ba(II)). The linear fit shows that an exponential fit of k_{obs} vs. pK_a is appropriate.

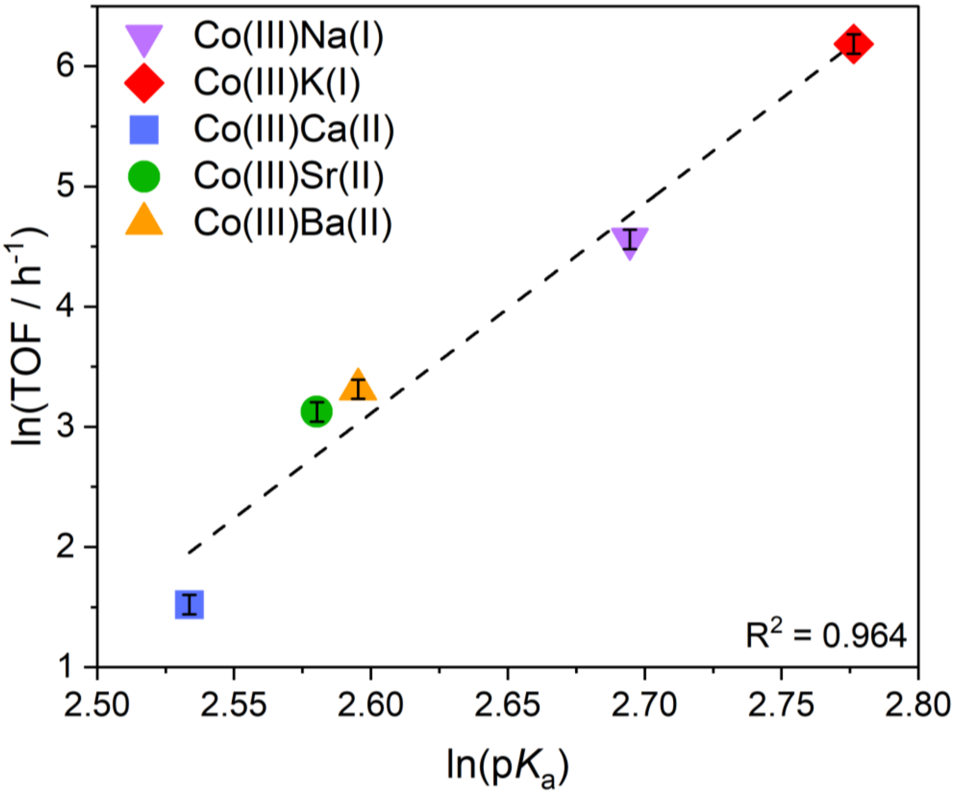

Supplementary Fig 73. Plot of ln(TOF) vs. ln(pK_a) for PO/CO_2 ROCOP catalysed by Co(III)M(I/II) (M(I/II) = Na(I), K(I), Ca(II), Sr(II), Ba(II)). The linear fit shows that an exponential fit of TOF vs. pK_a is appropriate.

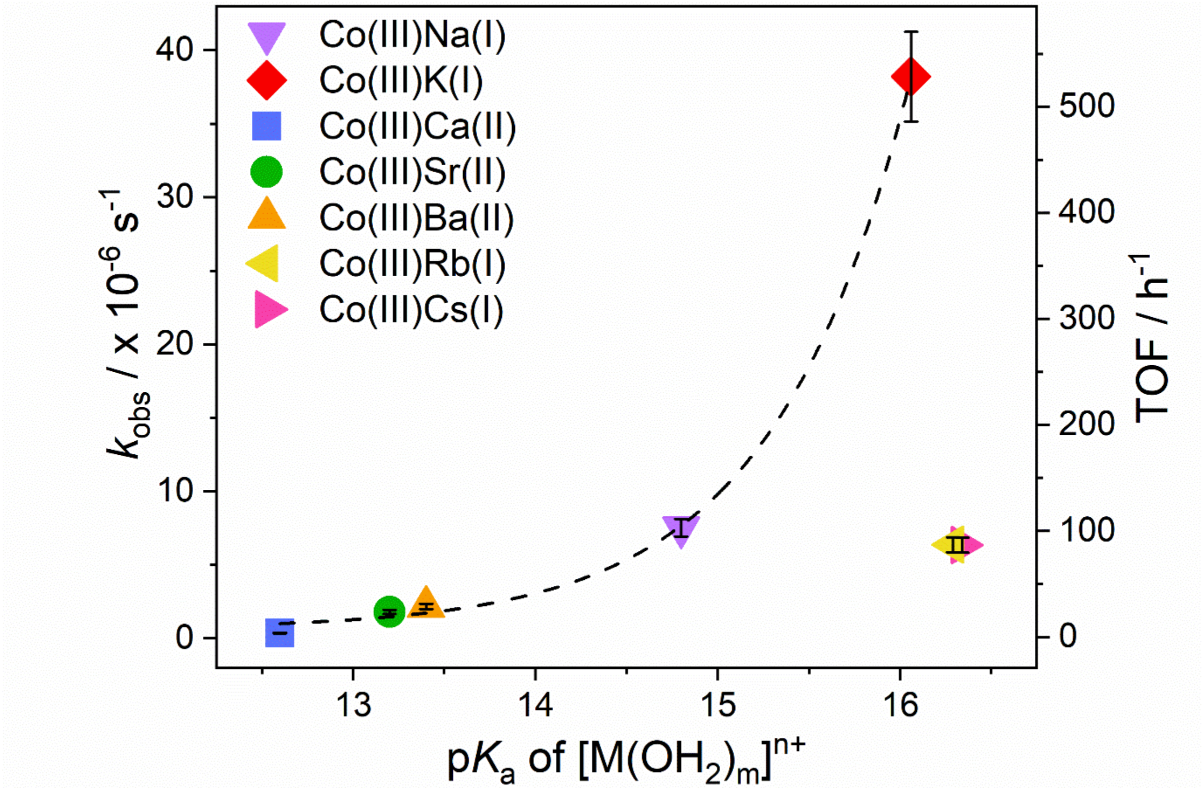

Supplementary Fig 74. Plot of TOF and k_{obs} against pK_a of the s-block metal for Co(III)M(I/II)-catalysed PO/CO_2 ROCOP, including values for Co(III)Cs(I) and Co(III)Rb(I).

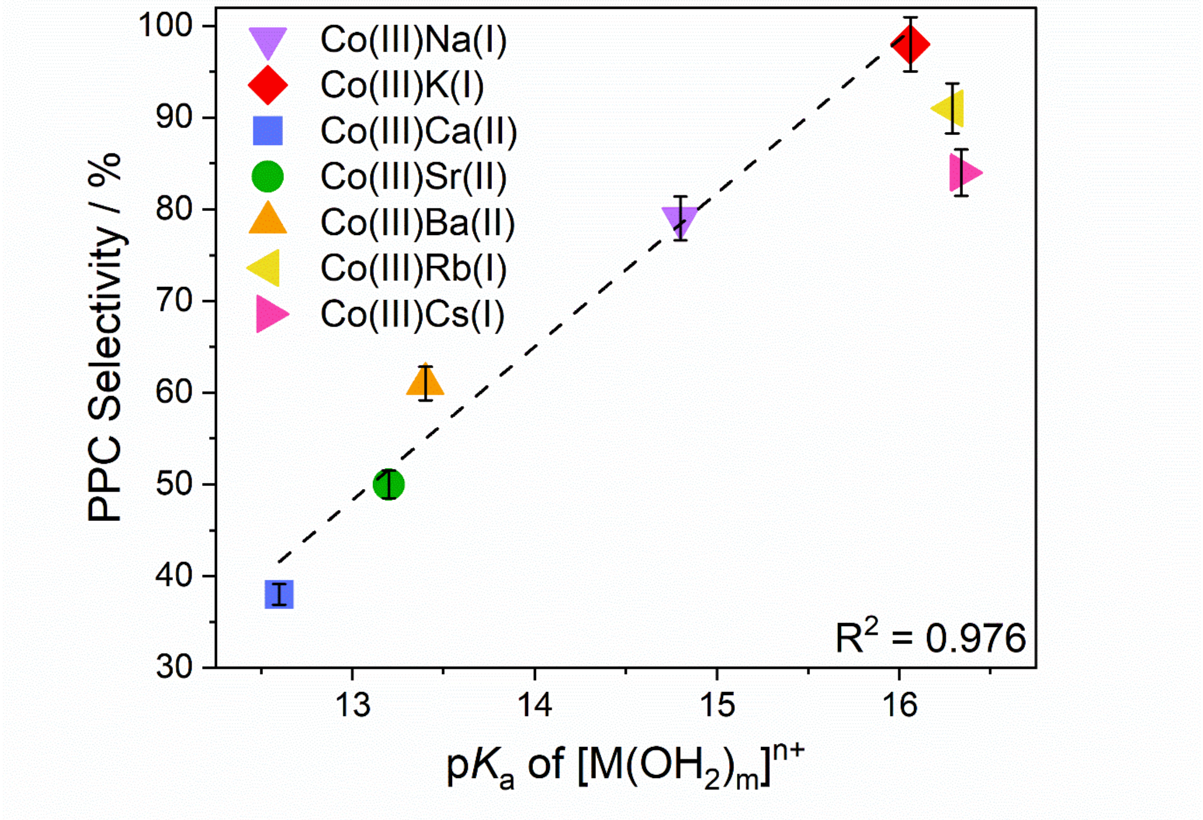

Supplementary Fig 75. Plot of selectivity against pK_a of the s-block metal for Co(III)M(I/II)-catalysed PO/CO_2 ROCOP, including values for Co(III)Cs(I) and Co(III)Rb(I).

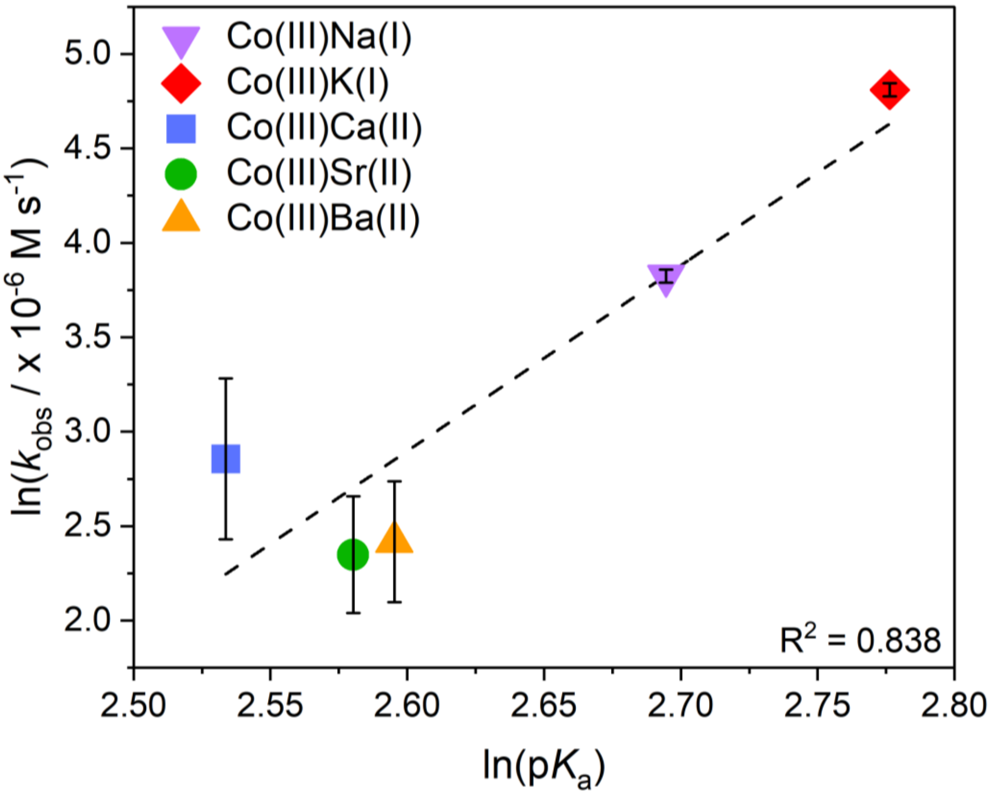

Supplementary Fig 76. Plot of ln(k_{obs}) vs. ln(pK_a) for PO/PA ROCOP catalysed by Co(III)M(I/II) (M(I/II) = Na(I), K(I), Ca(II), Sr(II), Ba(II)). The linear fit shows that an exponential fit of k_{obs} vs. pK_a is appropriate.

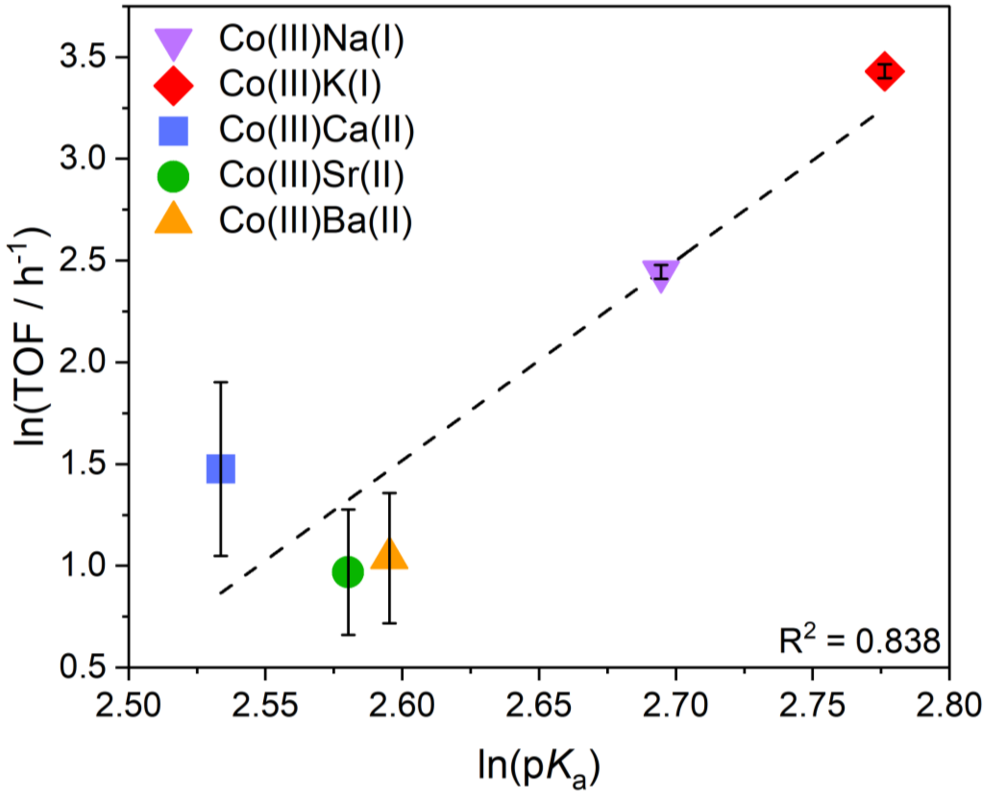

Supplementary Fig 77. Plot of ln(TOF) vs. ln(pK_a) for PO/PA ROCOP catalysed by Co(III)M(I/II) (M(I/II) = Na(I), K(I), Ca(II), Sr(II), Ba(II)). The linear fit shows that an exponential fit of TOF vs. pK_a is appropriate.

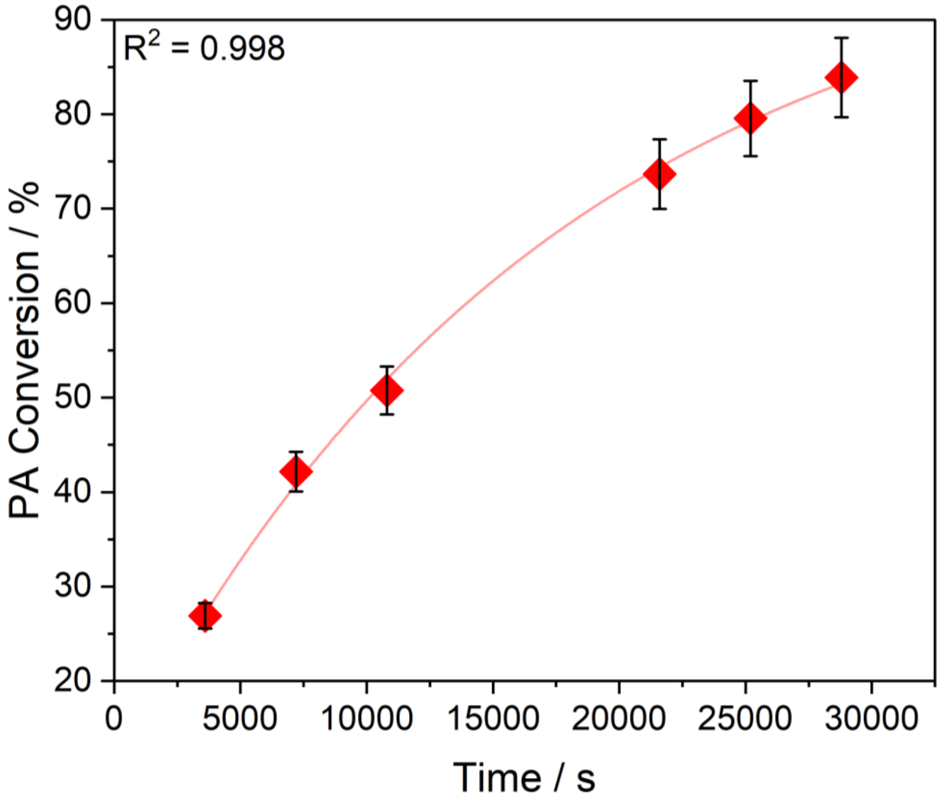

Supplementary Fig 78. Plot of [PA] against time for PO/PA ROCOP catalysed by Co(III)K(I) using 1:100:150 $[Cat]_0:[PA]_0:[PO]_0$ in THF.

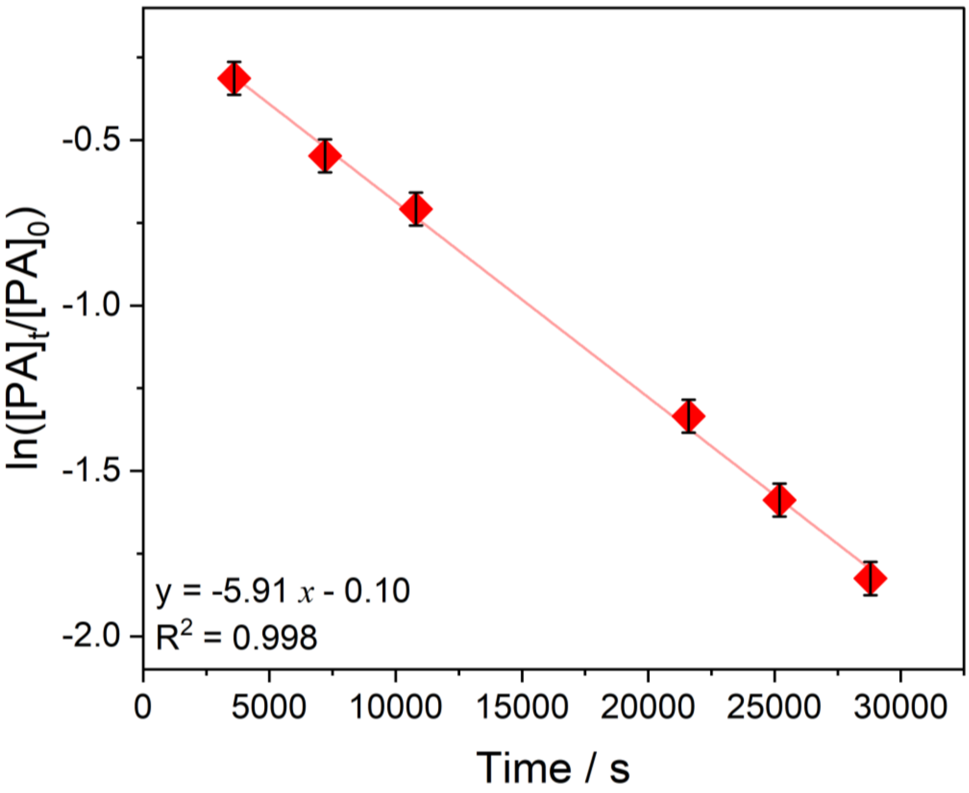

Supplementary Fig 79. Plot of $\ln([PA]_t/[PA]_0)$ against time for PO/PA ROCOP catalysed by Co(III)K(I) using 1:100:150 $[Cat]_0:[PA]_0:[PO]_0$ in THF.

When PO/PA ROCOP polymerisations are conducted with a large excess of PO (1:100:1000 $[cat]_0:[PA]_0:[PO]_0$), pseudo zero-order kinetics are observed, as can be seen by linear plots of PA conversion against time (Supplementary Fig 36). When the excess of PO is reduced to 1:200:250 $[cat]_0:[PA]_0:[PO]_0$, an exponential

conversion vs. time plot is observed, which gives a linear plot when ln([PA]$_t$/[PA]$_0$) is plotted against time (Supplementary Figs S79 and S80). This indicates that this catalytic system has a zero-order rate-dependence on concentration of PA, and a first-order rate dependence on concentration of PO.

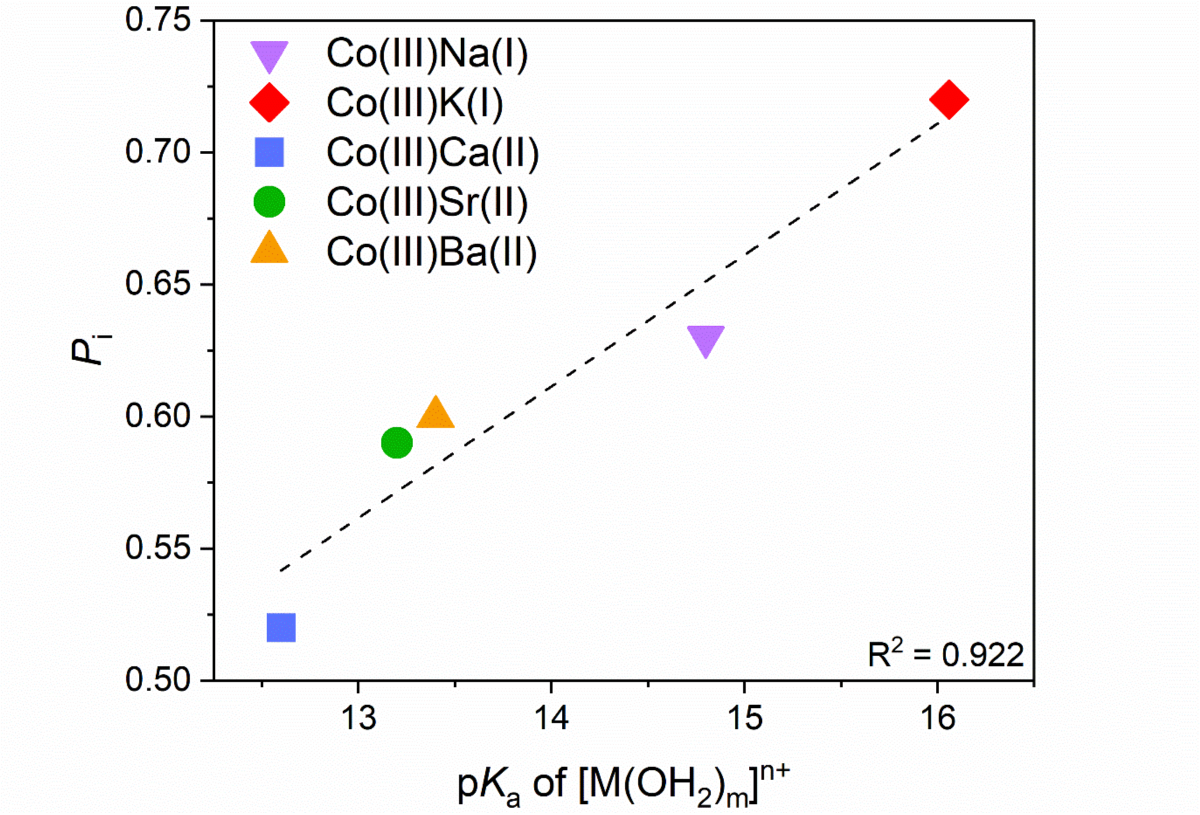

Supplementary Fig 80: Plot of P_i of PLA vs. the pK_a of the s-block metal for *rac*-LA ROP catalysed by Co(III)M(I/II) (M(I/II) = Na(I), K(I), Ca(II), Sr(II), Ba(II)).

Crystallographic Data for Co(III)M(II) (M(II) = Ca(II), Sr(II) and Ba(II)

Supplementary Tab 10: Selected bond lengths for Co(III)M(II) (M(II) = Ca(II), Sr(II), Ba(II)).

**Co(III)Ca(II) (022rwfk21)**		**Co(III)Sr(II) (006ff22_ff)**		**Co(III)Ba(II) (003ff21-ff)**	
**Bond/Separation**	**Length (°)**	**Bond/Separation**	**Length (°)**	**Bond/Separation**	**Length (°)**
Co1—Ca1	3.2744 (7)	Sr1—Co1	3.4742 (6)	Ba1—Co1	3.6198 (8)
Co1—O1	1.893 (2)	Sr1—O1	2.549 (2)	Ba1—O1	2.692 (3)
Co1—O7	1.915 (2)	Sr1—O6	2.529 (2)	Ba1—O6	2.663 (3)
Co1—O6	1.901 (2)	Sr1—O8	2.618 (2)	Ba1—O2	2.795 (3)
Co1—O9	1.917 (2)	Sr1—O5	2.764 (2)	Ba1—O8	2.718 (4)
Co1—N2	1.904 (3)	Sr1—O10	2.676 (2)	Ba1—O10	2.884 (4)
Co1—N1	1.902 (3)	Sr1—O2	2.704 (2)	Ba1—O5	2.819 (4)
Ca1—O1	2.348 (2)	Sr1—O4	2.772 (3)	Ba1—O4	2.839 (4)
Ca1—O6	2.404 (2)	Sr1—O3	2.751 (3)	Ba1—O3	2.832 (4)
Ca1—O5	2.743 (2)	Sr1—C27	3.111 (7)	Ba1—O12	2.830 (6)
Ca1—O8	2.578 (2)	Sr1—O12	2.698 (5)	Ba1—O11	2.892 (6)
Ca1—O2	2.689 (2)	Sr1—O11	2.793 (5)	Ba1—C27	3.212 (8)
Ca1—O10	2.378 (2)	Co1—O1	1.898 (2)	Co1—O1	1.902 (3)
Ca1—O3	2.707 (2)	Co1—O6	1.916 (2)	Co1—O6	1.914 (3)
Ca1—O4	2.652 (2)	Co1—O7	1.914 (2)	Co1—O9	1.910 (3)
Ca1—O11	2.280 (3)	Co1—O9	1.911 (2)	Co1—O7	1.924 (3)
O7—C23	1.297 (4)	Co1—N2	1.900 (3)	Co1—N1	1.900 (4)
O9—C25	1.287 (4)	Co1—N1	1.898 (3)	Co1—N2	1.896 (4)
O8—C23	1.239 (4)	O7—C23	1.293 (4)	O9—C25	1.285 (6)
O10—C25	1.238 (4)	O8—C23	1.238 (4)	O8—C23	1.235 (7)
N2—C3	1.276 (5)	O9—C25	1.285 (4)	O7—C23	1.289 (6)
N1—C22	1.275 (5)	O10—C25	1.243 (4)	O10—C25	1.247 (6)
O11—C27	1.221 (5)	N2—C3	1.274 (5)	N1—C22	1.276 (7)
		N1—C22	1.276 (5)	N2—C3	1.283 (7)
		C27—O12	1.160 (7)	O12—C27	1.211 (10)
		C27—O11	1.192 (7)	O11—C27	1.200 (10)

Supplementary Tab 11: Selected bond angles for Co(III)M(II) (M(II) = Ca(II), Sr(II), Ba(II)).

**Co(III)Ca(II) (022rwfk21)**		**Co(III)Sr(II) (006ff22_ff)**		**Co(III)Ba(II) (003ff21-ff)**	
**Bond**	**Angle (°)**	**Bond**	**Angle (°)**	**Bond**	**Angle (°)**
O1—Co1—N1	175.89 (11)	O1—Sr1—O4	144.94 (8)	O1—Ba1—O4	139.99 (11)
O7—Co1—O9	178.30 (9)	O2—Sr1—O5	175.17 (8)	O6—Ba1—O3	156.78 (12)
O6—Co1—N2	177.52 (10)	O6—Sr1—O3	157.38 (10)	O2—Ba1—O5	165.90 (11)
O1—Ca1—O4	142.29 (8)	O9—Co1—O7	177.06 (10)	O9—Co1—O7	175.84 (15)
O6—Ca1—O3	148.85 (7)	N1—Co1—O1	175.91 (13)	N1—Co1—O6	176.94 (17)
O2—Ca1—O5	169.91 (7)	N2—Co1—O6	177.45 (11)	N2—Co1—O1	175.49 (19)

Supplementary Tab 12. Summary of crystallographic refinement data for Co(III)M(II) (M(II) = Ca(II), Sr(II), Ba(II)).

**Complex**	**Co(III)Ca(II)**	**Co(III)Sr(II)**	**Co(III)Ba(II)**
**Local Code**	022rwfk21	006ff22_ff	003ff21-ff
**CCDC Deposition Number**	2250536	2250537	2250538
**Crystal data**			
**Chemical formula**	$C_{28}H_{33}CaCoN_2O_{12}\cdot H_2O$	$C_{28}H_{33}CoN_2O_{12}Sr$	$C_{28}H_{33}BaCoN_2O_{12}$
M_r	706.59	736.11	785.83
**Crystal system, space group**	Monoclinic, $P2_1/c$	Tetragonal, $P4/n$	Tetragonal, $P4/n$
***a*, *b*, *c* (Å)**	9.8086 (2), 18.7781 (3), 16.0772 (3)	27.1904 (1), 27.1904 (1), 9.8214 (1)	27.3281 (3), 27.3281 (3), 9.8247 (2)
**α, β, γ (°)**	90, 91.328 (2), 90	90, 90, 90	90, 90, 90
V (Å^3)	2960.41 (9)	7261.14 (9)	7337.3 (2)
**Z**	4	8	8
**μ (mm^{-1})**	6.69	6.00	12.31
**Crystal size (mm)**	0.29 × 0.16 × 0.09	0.27 × 0.20 × 0.14	0.12 × 0.07 × 0.06
**Data collection**			
**Absorption correction**	Gaussian *CrysAlis PRO* 1.171.40.53 (Rigaku Oxford Diffraction, 2019) Numerical absorption correction based on gaussian integration over a multifaceted crystal model Empirical absorption correction using spherical harmonics, implemented in SCALE3 ABSPACK scaling algorithm.	Multi-scan *CrysAlis PRO* 1.171.41.117a (Rigaku Oxford Diffraction, 2021) Empirical absorption correction using spherical harmonics, implemented in SCALE3 ABSPACK scaling algorithm.	Multi-scan *CrysAlis PRO* 1.171.41.117a (Rigaku Oxford Diffraction, 2021) Empirical absorption correction using spherical harmonics, implemented in SCALE3 ABSPACK scaling algorithm.
T_{min}, T_{max}	0.476, 1.000	0.541, 1.000	0.166, 1.000
**No. of measured, independent and observed [$I > 2\sigma(I)$] reflections**	17176, 6137, 5143	182489, 7602, 6671	80806, 7677, 5819
R_{int}	0.036	0.064	0.094
**(sin θ/λ)$_{max}$ (Å^{-1})**	0.630	0.630	0.630
**Refinement**			
$R[F^2 > 2\sigma(F^2)]$, $wR(F^2)$, S	0.048, 0.139, 1.02	0.047, 0.133, 1.04	0.049, 0.116, 1.03
**No. of reflections**	6137	7602	7677
**No. of parameters**	412	415	400
**No. of restraints**	0	18	18
	$w = 1/[\sigma^2(F_o^2) + (0.0766P)^2 + 2.3292P]$ where $P = (F_o^2 + 2F_c^2)/3$	$w = 1/[\sigma^2(F_o^2) + (0.0693P)^2 + 8.4614P]$ where $P = (F_o^2 + 2F_c^2)/3$	$w = 1/[\sigma^2(F_o^2) + (0.0408P)^2 + 17.2275P]$ where $P = (F_o^2 + 2F_c^2)/3$
$\Delta\rho_{max}$, $\Delta\rho_{min}$ (e Å^{-3})	0.61, −0.69	0.72, −0.56	0.83, −0.46

References

1 Kowalski, A., Duda, A. & Penczek, S. Polymerization of l,l-Lactide Initiated by Aluminum Isopropoxide Trimer or Tetramer. Macromolecules 31, 2114-2122 (1998).

2 Deacy, A. C., Moreby, E., Phanopoulos, A. & Williams, C. K. Co(III)/Alkali-Metal(I) Heterodinuclear Catalysts for the Ring-Opening Copolymerization of CO2 and Propylene Oxide. J. Am. Chem. Soc. 142, 19150-19160 (2020).

3 Izatt, R. M. et al. Calorimetric titration study of the interaction of several uni- and bivalent cations with 15-crown-5, 18-crown-6, and two isomers of dicyclohexo-18-crown-6 in aqueous solution at 25 °C and μ = 0.1. J. Am. Chem. Soc. 98, 7620-7626 (1976).

4 Wang, L. et al. Kinetic Analysis of the Living Ring-Opening Polymerisation of L-Lactide with Tin(II) Initiators. Eur. J. Inorg. Chem. 2013, 5896-5905 (2013).

5 Shannon, R. D. Revised effective ionic radii and systematic studies of interatomic distances in halides and chalcogenides. Acta Crystallogr. Sect. A 32, 751-767 (1976).

6 Schindler, A. & Harper, D. Poly(lactic acid). I. Stereosequence distribution in the polymerization of racemic dilactide. J. Polym. Sci., Polym. Let.. Ed. 14, 729-734 (1976).
